# Comparative analysis of the genetic variability within the Q-type C2H2 zinc-finger transcription factors in the economically important cabbage, canola and Chinese cabbage genomes

**DOI:** 10.1186/s41065-018-0065-5

**Published:** 2018-09-21

**Authors:** Susan D. Lawrence, Nicole G. Novak

**Affiliations:** Invasive Insect Biocontrol and Behavior Lab, USDA-ARS, 10300 Baltimore Ave., BARC-West Bldg 007, Rm 301, Beltsville, MD 20705 USA

**Keywords:** Brassica, Q-type C2H2 zinc finger transcription factors, Cabbage, Canola

## Abstract

**Background:**

*Brassica oleracea, B. rapa* and *B. napus* encompass many economically important vegetable and oil crops; such as cabbage, broccoli, canola and Chinese cabbage. The genome sequencing of these species allows for gene discovery with an eye towards discerning the natural variability available for future breeding. The Q-type C2H2 zinc-finger protein (ZFP) transcription factors contain zinc finger motifs with a conserved QALGGH as part of the motif and they may play a critical role in the plants response to stress. While they may contain from one to five ZF domains (ZFD) this work focuses on the ZFPs that contain two zinc-fingers, which bind to the promoter of genes, and negatively regulate transcription via the EAR motif. *B. oleracea* and *rapa* are diploid and evolved into distinct species about 3.7 million years ago. *B. napus* is polyploid and formed by fusion of the diploids about 7500 years ago.

**Results:**

This work identifies a total of 146 Q-type C2H2-ZFPs with 37 in *B. oleracea*, 35 in *B. rapa* and 74 in *B. napus*. The level of sequence similarity and arrangement of these genes on their chromosomes have mostly remained intact in *B. napus*, when compared to the chromosomes inherited from either *B. rapa* or *oleracea*. In contrast, the difference between the protein sequences of the orthologs of *B. rapa* and *oleracea* is greater and their organization on the chromosomes is much more divergent. In general, the 146 proteins are highly conserved especially within the known motifs. Differences within subgroups of ZFPs were identified. Considering that *B. napus* has twice the number of these proteins in its genome, RNA-Seq data was mined and the expression of 68 of the 74 genes was confirmed.

**Conclusion:**

Alignment of these proteins gives a snapshot of the variability that may be available naturally in *Brassica* species. The aim is to study how different ZFPs bind different genes or how dissimilar EAR motifs alter the negative regulation of the genes bound to the ZFP. Results from such studies could be used to enhance tolerance in future *Brassica* breeding programs.

**Electronic supplementary material:**

The online version of this article (10.1186/s41065-018-0065-5) contains supplementary material, which is available to authorized users.

## Background

Q-type C2H2 zinc finger proteins (ZFP) are transcription factors. “Q-type” refers to the invariant QALGGH sequence found in the zinc finger domains, and C2H2 characterizes the two cysteine and two histidine residues found in each finger. These residues bind a zinc ion that stabilizes the ZFP and allows binding specificity to a domain within the promoter of the gene it regulates. First discovered in petunia by Takatsuji et al. [1], a total of 21 Q-type C2H2 ZFPs have been described in that species [2]. Using in silico methods, Englbrecht et al. [3] described 3 groups of ZFPs in Arabidopsis; A, B and C, with the C family divided into three additional groups (C1, C2 and C3) depending on the number of spaces between the invariant histidines. There are 64 members in the C1 family that contain either a single or a cluster of two to five zinc finger domains (ZFDs). The C1 family has three amino acids between the histidines and contains many proteins responsive to environmental stress [4, 5]. In Arabidopsis, the 18 two fingered Q-type C2H2 ZFP proteins are members of the C1-2i and here will be referred to as ZFPs. The Arabidopsis proteins cluster into five groups named 2i-A-D with an outlier-X [3]. These ZFPs include a conserved domain containing the amino acids DLN. It is similar to the first active repression motif described in plants [6], which was named the **e**thylene-**r**esponsive element-binding **f**actor (ERF)-associated **a**mphiphilic **r**epression (or EAR) domain. A role for the EAR motif as an active repressor was also demonstrated in ZFPs of Arabidopsis [7]. Ectopic expression of ZFPs can lead to an increase in tolerance to specific stresses [5]. Subsequently additional studies identifying all forms of C2H2 ZFPs have been undertaken in for example rice, foxtail millet, poplar and crocus [8–11]. Several other studies focused specifically on the Q-type C2H2 TFs, for example in poplar, or bread wheat [12, 13]. Generally, these studies utilize the availability of a published genome sequence, however, studies in bread wheat (a hexaploid organism) and crocus relied on ESTs from public databases [11, 13]. The work described in this manuscript, catalogs the Q-type ZFPs in three Brassica species. Therefore, naturally occurring variants within these proteins can be utilized in subsequent studies to identify how the altered sequences could affect gene expression. ZFPs might be useful as tools for breeding increased tolerance to biotic or abiotic stresses encountered by cole crops.

In the current study, we identify ZFPs of economically important Brassica species, and analyze their structure and their expression using RNA-Seq data from previously published work. To cast a wide net for capturing genetically related ZFPs, three related species were examined and compared to Arabidopsis ZFPs. Arabidopsis is also a member of the Brassicaceae and evolved from the same ancestral progenitor as the Brassica species. The Arabidopsis lineage split from this ancestral progenitor approximately 20 million years ago (mya) with a whole genome duplication occurring in the Brassica lineage ~ 16 mya, which is reflected in a doubling in gene number when comparing Brassica to Arabidopsis [14]. Comparative mapping of Arabidopsis to several other similar species provides evidence for an ancestral Arabidopsis genome that has been divided into 24 blocks [15]. These have been reshuffled in present day Arabidopsis and these syntenic blocks have also been identified in modern Brassica species. Maps showing the reorganization of these blocks in *B. rapa* and *B. oleracea* have been published along with the genome sequences of these species [16, 17]. The blocks are not collinear in *B. oleracea* and *B. rapa* but have a different organizational pattern, probably due to chromosomal translocations during the time since these species split from their shared ancestor, about 3.7 mya [14]. These species are diploid, containing 9 and 10 chromosomes, respectively. Their genomes fused and duplicated to form *B. napus* ~ 7500 years ago [18]. Brassica species therefore are both diploid and polyploid. The triangle of U [19] represents their evolution with the diploid species *B. rapa, nigra* and *oleracea* at the points of the triangle containing genomes A, B and C respectively. Between the tips of the triangle are the polyploid species with *B. napus* along the side of the triangle between *B. rapa* and *B. oleracea* containing both A and C genomes, respectively. Since *B. rapa* contains 10 chromosomes and *B. oleracea* contains 9 chromosomes the *B. napus* genome still maintains 19 chromosomes. Indeed, the 19 *B. napus* chromosomes are labeled chromosome A1-A10 and C01-C09, reflecting the genomes from which they were derived. These species include many important oil and vegetable crops; such as broccoli, cabbage, Chinese cabbage and canola. Given the evolution of these crops, the comparison of the Q-type ZFPs in the three reference genomes should provide a picture of the genetic diversity available for testing the effects of different ZFPs on the biotic stress response in these species. Since this group of Q-type ZFPs have previously been associated with regulating environmental stress and can enhance tolerance to the stress [5], identification of these proteins in the commercially important Brassica species may lead to their use in breeding programs. This work identified 146 ZFPs in three Brassica species. Prior studies analyzing the ZFDs and EAR motifs mutated one protein at a time and demonstrated how this altered either DNA binding for the ZFDs or negative regulation with the modified EAR motif [2, 4, 5]. The study documented here begins to address how much variation in these motifs can occur naturally.

## Methods

Proteins with sequence similarity to the Arabidopsis Q-type two-fingered C2H2 TF, Zat11 were identified using BLASTX in Bolbase (http://www.ocri-genomics.org/bolbase/), a database accessing the annotated *Brassica oleracea* genome [20]. Proteins from *B. oleracea* containing two Q-type C2H2 zinc finger motifs were selected and their best Arabidopsis match was found using BLASTP in the Arabidopsis genome database TAIR (https://www.arabidopsis.org/Blast/index.jsp). Ensembl Plants was also used for identification of additional *B. oleracea* genes and genes within the *B. rapa* genomes http://plants.ensembl.org/index.html. Finally, Genescope was used to identify *B. napus* genes http://www.genoscope.cns.fr/brassicanapus/. Subsequently Ensembl Plants was also used for updating genes from *B. napus*.

For the phylogenetic analysis, proteins were aligned using CLUSTAL W and the phylogenetic tree was inferred using the Maximum likelihood method and the JTT matrix based model [21]. 1000 bootstrap replicates were performed [22]. Evolutionary analyses were conducted in MEGA6 [23]. Alignment of Q-type ZFPs was performed using CLUSTAL OMEGA from http://www.ebi.ac.uk/ Tools/msa/clustalo/. The proteins were analyzed using the program MEME (Multiple motifs for EM Elicitation) at (http://meme-suite.org/tools/meme) to identify conserved motifs. Nuclear localization domains were located using NLSMapper http://nls-mapper.iab.keio.ac.jp/cgi-bin/NLS_Mapper_form.cgi.

The NCBI (National Center for Biotechnology Information) SRA (Sequence Read Archive) was examined https://www.ncbi.nlm.nih.gov/sra for transcriptome studies of *B. oleracea* and *B. napus* to identify transcribed ZFPs*.* Ensembl Plants https://plants.ensembl.org/index.html and Genescope http://www.genoscope.cns.fr/brassicanapus/ were also used to catalog the location of ZFP genes on the genome.

## Results and discussion

### Identification and phylogenetic analysis of Brassica genes

The *B. rapa, oleracea* and *napus* genomes [16–18] were searched by BLASTX using the Arabidopsis ZFP, Zat11 and 146 unique genes were identified. Initially a search of Bolbase [20] identified 25 unique *B. oleracea* Q-type C2H2 TFs [24]. A subsequent examination of the *B. oleracea* genome from Ensembl Plants (https://plants.ensembl.org/index.html) led to identification of 12 more ZFP genes (see Additional file 1). All 146 ZFP genes mined in this study were renamed based on their species designation (Bo, Br, BnaA or BnaC) followed by a ZFP and numbered based on their positions on the chromosome map. For example, the ZFP named BrZFP1 is found at the top of chromosome A1 in the *B. rapa* genome. The original gene name and location on the genome is listed (see Additional file 1). Several protein sequences were modified from the original genome annotation, based on comparison with the other closest homologs. These comparisons revealed that several of the originally annotated *B. napus* gene sequences included an intron that interrupted the conceptual translation and resulted in loss of protein sequence present in either the *B. oleracea* or the *B rapa* orthologs. The intron sequences were added back to these transcripts and re-translated, which resulted in proteins highly identical to those predicted from the diploid genomes of *B. oleracea* and *B. rapa*. A dataset has been published examining the *Brassica napus* transcriptome [25], in which our protein sequences were confirmed in the A (*B. rapa*) derived genome. The altered genes were given the prefix My and the protein sequences are shown (see Additional file 1) and are also aligned (see Additional file 2) to pinpoint the genes altered from the databases.

**Fig. 1:**
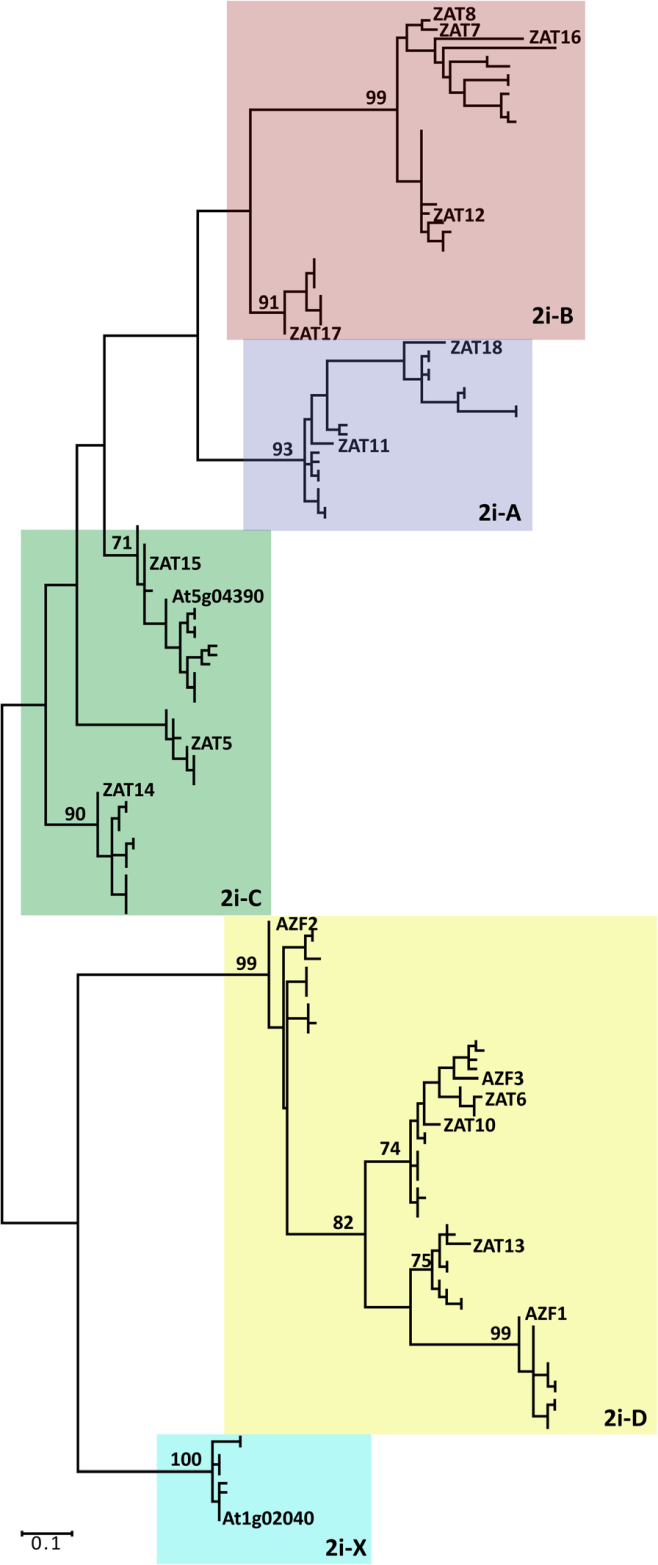
Skeletonized phylogenetic tree of *Brassica* and Arabidopsis (Zats) Q-type ZFPs form 5 groups. The tree shows the position of the 18 Zats and the major bootstrap values. Groups are delineated as 2i-B, 2i-A, 2i-C, 2i-D and 2i-X in pink blue, green, yellow and turquoise, respectively. The proteins were aligned with CLUSTALW and the phylogenetic tree was generated using the maximum likelihood method with 1000 bootstrap replicates. A complete tree with the names of all *Brassica* and Arabidopsis proteins are in (Additional file 3)

A phylogenetic map containing the 18 Arabidopsis proteins and 146 proteins from the three Brassica species (see Additional file 3) was generated using CLUSTALW and maximum likelihood analysis in MEGA6 software [23]. The Brassica proteins mapped into 5 groups clustering with the Arabidopsis proteins, which will also be referred to as groups 2i-A-D and X (Fig. 1). Alignments of the protein sequences using CLUSTAL OMEGA without Arabidopsis sequences confirmed the 5-subgroups (2i-A-D and X) found for the Arabidopsis proteins (see Additional file 2).

### Comparative mapping of *Brassica* ZFPs

**Fig. 2:**
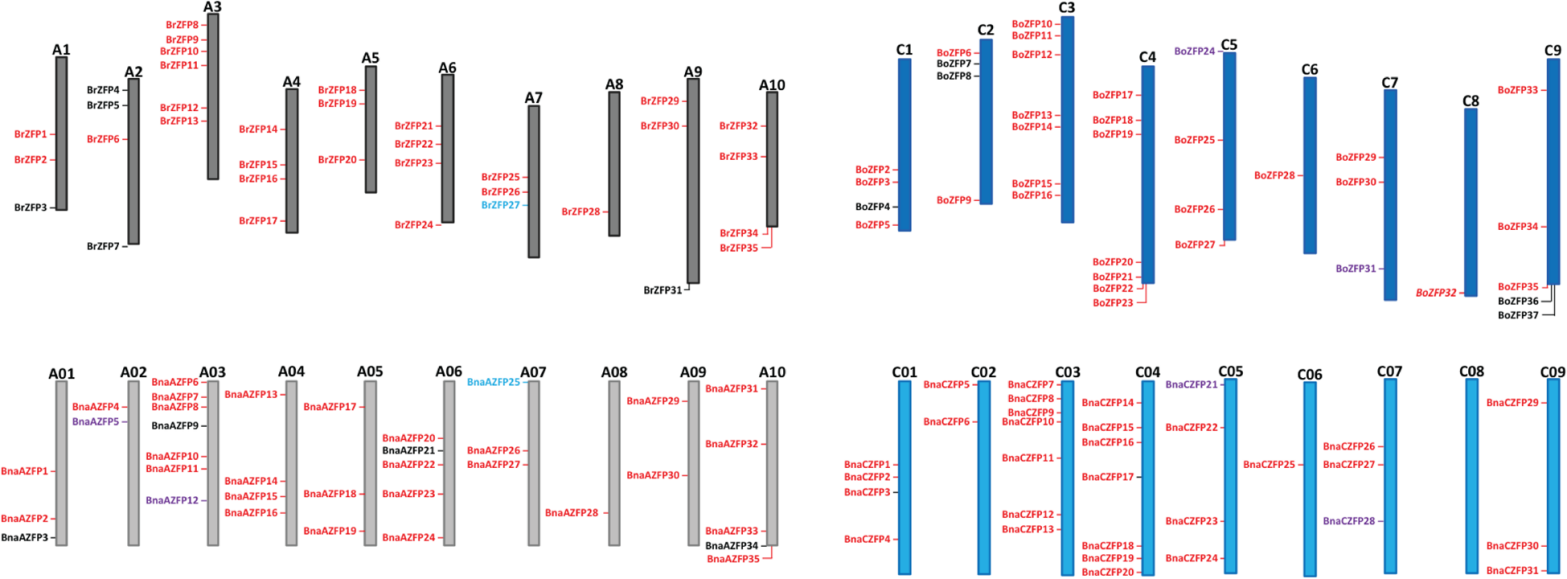
Comparison of ZFP proteins on diploid Brassica species and the polyploid *Brassica napus*. Chromosomes of *Brassica rapa* in dark gray (BrZFP) *Brassica oleracea* in dark blue (BoZFP) to *Brassica napus* with the A genome in light gray (BnaAZFP) and C genome in light blue (BnaCZFP). BnaAZFP36, 37 and 38 are unmapped. Within the 35 BnaAZFP mapped genes, 83% (29) map to similar positions as BrZFP genes. Of the 38 BnaAZFP proteins 84% (32) have 97–100% (red) identity and 3 have 93–96% (purple). The final 3 proteins (black) have 81% (BnaAZFP9), 56% (BnaAZFP21) and 85% (BnaAZFP34) identity to the Br homolog. Generally, the BoZFP and the BnaCZFP genes map to the same location on the chromosome with either 96–100% (red) or 93–95% (purple) identity. BoZFP20 and BoZFP32 have homologous proteins that are currently unmapped in the *B. napus* genome (BnaCZFP32 and BnaCZFP33), while BoZFP4 and BoZFP8 (black) have no homolog in the *B. napus* genome. BoZFP36 and BoZFP37 (black) are currently unmapped on the *B. oleracea* genome and they are homologous to BnaCZFP34 and BnaCZFP35, which are also unmapped. BoZFP7 (black) is a homolog and maps to a *B. napus* gene that contains only a portion of the ORF

A map of the chromosomes comparing the position of ZFP genes from *B. oleracea, rapa* to *napus* is shown (Fig. 2). Most ZFP genes map not only to the same chromosome between the species, but to a similar location within the chromosome. The orthologs also generally contain 97–100% identity in protein sequence. *Brassica rapa* contains 35 ZFPs, while the A genome (derived from *B. rapa*) of *B. napus* contains 38 ZFPs. Once again a comparison of the A genome (*B. rapa* derived) of *B. napus* (A01-A10) with that of *B. rapa* (A1-A10) shows the co-linearity between the ZFP genes along the same chromosome and within the same general location on the chromosome. The orthologs between these two species of the A genome also show between 97 and 100% identity in their protein sequences. Comparison of the C genome (*B. oleracea* derived) chromosomes of *B. napus* (C01-C09) with *B. oleracea* (C1-C9) shows that there are 37 ZFPs in the *B. oleracea* genome and 36 ZFPs in the *B. napus* C genome. Two *B. oleracea*, 5 BnaC and 3 BnaA ZFP genes remain to be mapped onto chromosomes (see Additional file 1). However mapped ZFPs show the same strong percent identity to their unmapped orthologs. For example, BoZFP20 and BnaCZFP32 protein sequences are 100% identical, but the *B. napus* gene is currently unmapped.

**Fig. 3:**
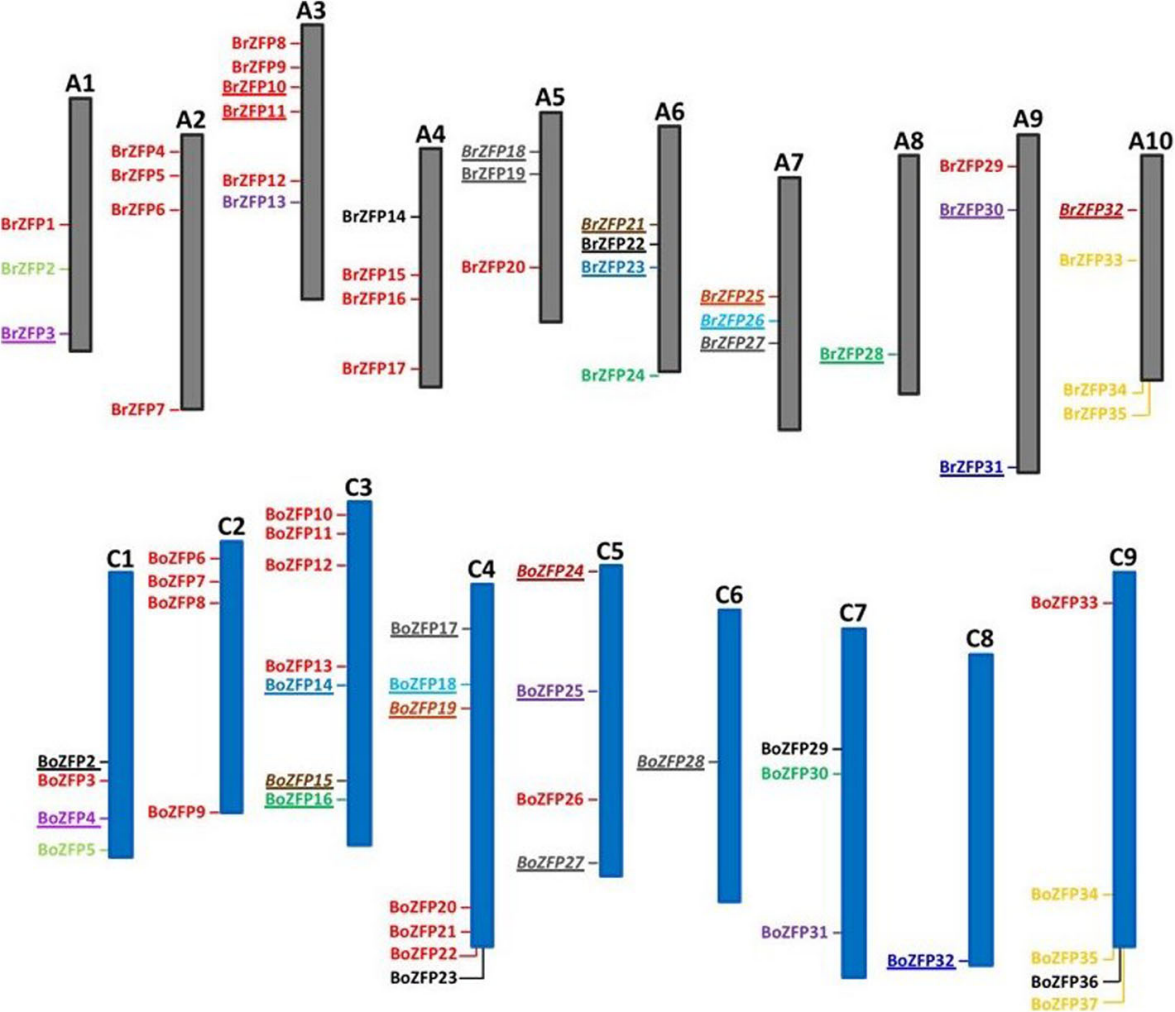
Comparison of map position and percent identity between the diploid Brassicas. *Brassica rapa* (top) and *Brassica oleracea* (bottom) chromosomes with red gene names containing protein sequences that are 92–98% identical, map to the same chromosome and are in identical order. BrZFP10 and BrZFP11 are tandem duplications. Genes in black are not present in the other species (BoZFP23, BoZFP29, BoZFP36). BrZFP14 is a homolog of BoZFP1, which is currently unmapped. Additional homologous pairs between the two species are identified by like colors and with or without underline or italics. These additional genes generally do not appear in the same genomic location. An exception is the 3 genes in orange on either chr 9 of *B. oleracea* or chr 10 of *B. rapa*

Comparisons of the map positions and protein sequence identities between ZFP genes of the diploid species, *B. rapa* and *B. oleracea* (Fig. 3) show more divergence in comparison to *B. napus*, as would be expected from species that appeared so much earlier than the formation of *B. napus*. Only 13 ZFP genes map to the same location and their gene products exhibit 92–98% protein sequence identity. Three genes in *B. oleracea* from chromosome 9 map to a similar location on chromosome 10 of *B. rapa*. This supports similar findings that the A and C genomes of *B. oleracea* and *B. rapa* have had more opportunity to diverge from one another than they have from the *B. napus* genome.

### Evolution of Arabidopsis and Brassica ZFPs

The number of orthologs of the eighteen Arabidopsis ZFPs in the *Brassica oleracea, rapa* and A and C genome of *B. napus* is shown in Tables 1 and 2. There are generally 2 or 3 homologs of most *Arabidopsis* ZFPs represented in the *B. oleracea* and *B. rapa* genomes, which is consistent with the genome duplication event that took place in the *Brassica* progenitor at about 16 mya after the split from *Arabidopsis*. While Zat12 has four homologs in *B. rapa*, one of these is a tandem duplication and probably arose after the evolution of *B. napus*. Only two of the Arabidopsis genes (Zat6 and AZF3) have a single homolog in *B. oleracea* or *B. rapa*, and there are no homologs in the *Brassica* genomes for Zat7 and Zat8. However, these are tandem duplications in the Arabidopsis genome of Zat16, suggesting that they may have arisen after the split of Arabidopsis from the *Brassica* species. Zat16 does have two homologs in both *B. oleracea* and *B. rapa*.

**Table 1:** Number of Arabidopsis ZFP homologs in the Brassica A and C genome

Gene Name	Arabidopsis Gene ID	# of Bo homologs	# of Br homologs	# of BnaA homologs	# of BnaC homologs
2i-X	At1g02040	3	2	1	3
Zat10 (2i-D)	At1g27730	3	2	2	3
ZAT5 (2i-C)	At2g28200	2	2	2	2
ZAT17 (2i-B)	At2g28710	2	2	2	2
ZAT11 (2i-A)	At2g37430	2	2	3	3
ZAT15 (2i-C)	At3g10470	2	2	2	1
AZF2 (2i-D)	At3g19580	3	3	3	3
ZAT16 (2i-B)	At3g46070	2	2	3	2
ZAT8 (2i-B)	At3g46080	0	0	0	0
ZAT7 (2i-B)	At3g46090	0	0	0	0
ZAT13 (2i-D)	At3g49930	2	2	2	3
ZAT18 (2i-A)	At3g53600	2	2	2	2
ZAT14 (2i-C)	At5g03510	3	3	4	3
ZAT6 (2i-D)	At5g04340	1	0	1	1
(2i-C)	At5g04390	3	3	3	2
AZF3 (2i-D)	At5g43170	1	1	1	1
ZAT12 (2i-B)	At5g59820	3	4	4	2
AZF1 (2i-D)	At5g67450	3	3	3	3

**Table 2:**
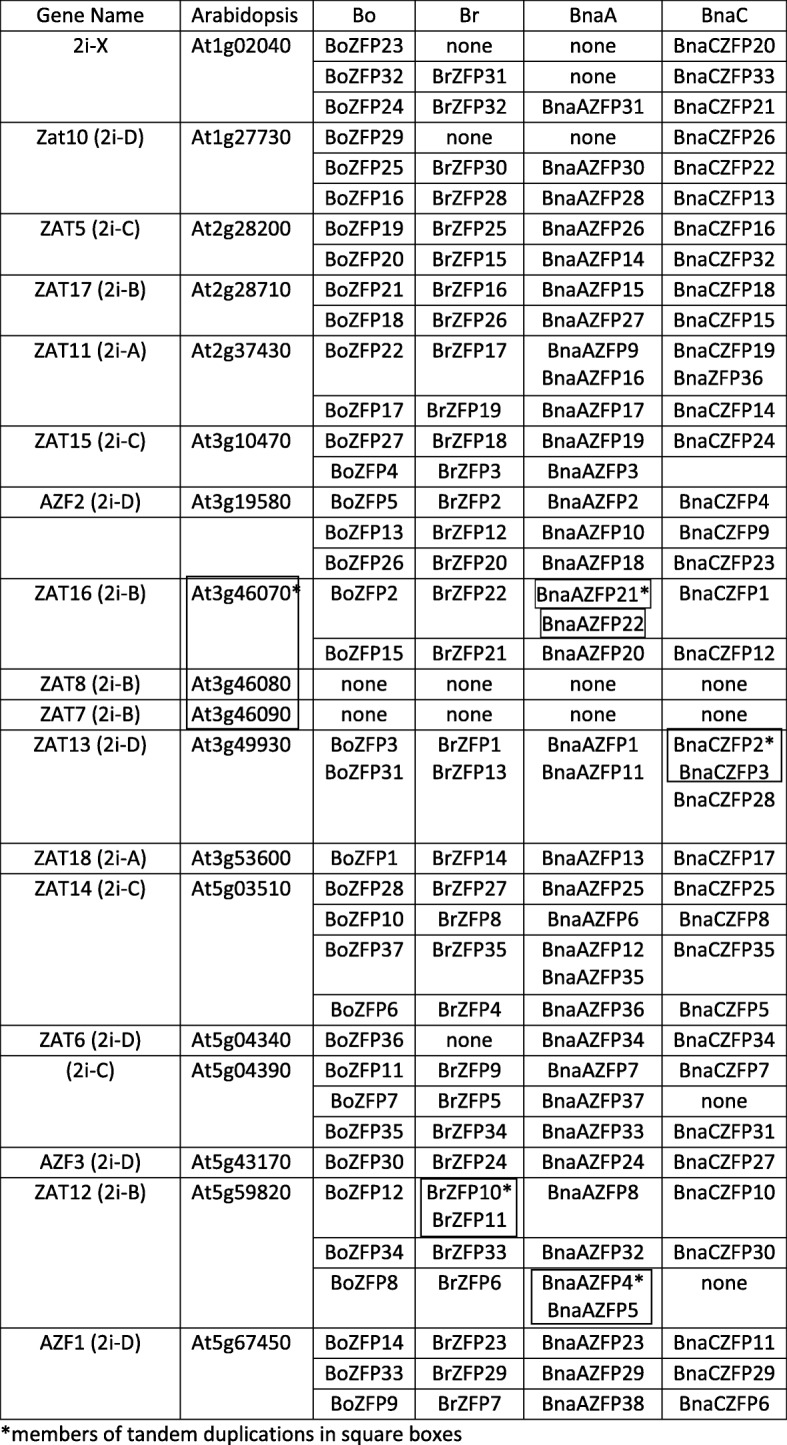
Homologs of ZFPs from Arabidopsis and Brassica A and C genome

*members of tandem duplications in square boxes

Generally, when two genomes combine and double their chromosomes, genes are lost over evolutionary time to compensate for the massive increase in genes. However, *B. napus* is a relatively new polyploid (forming ~ 7500 years ago) and may not have completely achieved this balance. The number of Q-type-ZFPs found in *B. napus* is 74, which is slightly more than double the amount in the diploid species. There have also been tandem duplications of genes in *B. rapa* and both the BnaA and BnaC genomes, which are not present in the other genomes, suggesting that they occurred after splitting (in the case of the diploids) or merging in the case of the tetraploid (Table 2). There were tandem duplications in the BnaA genome for Zat12 and Zat16 and Zat11. In *B. napus* there are a total of 3 Zat15 homologs and all three are 100% identical to their *B. oleracea* and *B rapa* homologs. Since the diploid genomes have two Zat15 homologs each, the *B. napus* genome has lost one (Table 2).

### Conserved protein motifs and zinc finger spacing in Brassica ZFPs

**Fig. 4:**
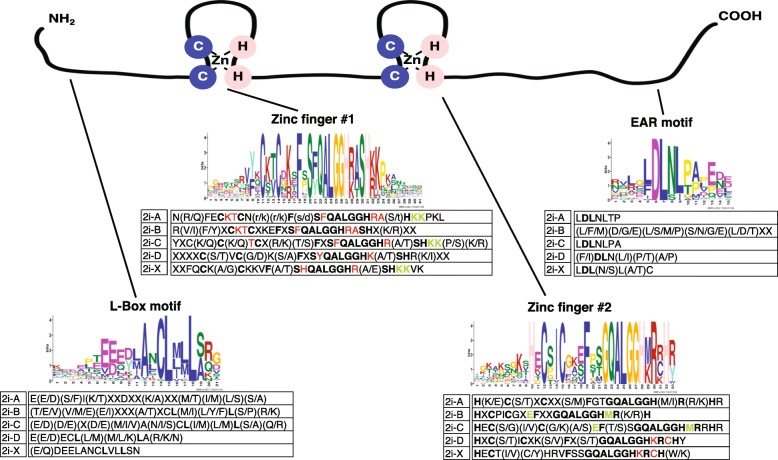
Conserved motifs identified in 146 Brassica ZFP proteins and in all five separate ZFP groups. A cartoon (top) shows the position of the conserved motifs on the protein sequence. Consensus motifs common to all 146 Q-type ZFPs from *Brassica oleracea, rapa* and *napus* include, 2 zinc finger motifs with invariant QALGGH (center) and an L-box (left) and EAR-motif (right). Amino acids in bold black are invariant within the composite motifs. Red and green amino acids are common to more than one of the subgroups within either the first or second zinc finger motifs. Alignment of all Brassica proteins in each subgroup confirms the presence of the motif variations (see Additional file 2)

To understand the variation in conserved protein motifs in the Brassica ZFPs, the MEME Suite (http://meme-suite.org/tools/meme) was used to identify common motifs in all 146 Brassica ZFPs and separately in each subgroup (2i-A-D and X). There are four conserved motifs in the Q-type two fingered ZFPs (Fig. 4). The first motif is the L-box, a leucine rich domain near the amino terminal end of the protein. The two zinc fingers are also conserved, with an invariant QALGGH in both zinc finger motifs and unique characteristics within these two motifs. The EAR motif was also examined for each group of Brassica ZFPs. A composite motif for each of the two zinc fingers, the L-box and EAR motif is presented (Fig. 4). Variations and similarities in amino acids within the motifs for each subgroup (2iA-D and X) are also shown (Fig. 4). The first zinc finger motif contains the following invariant amino acid sequence ‘CXXCXXXFXSXQALGGHXXXH’ with X being a placeholder representing variable residues within the protein sequence. The 2i-A and B groups have a KT between the two invariant Cs, and an F between the invariant S and Q. Finally, the first zinc finger motif of the 2i-A group ends with RAXHKKPKL while the 2i-B group ends with RASHXXXX. The 2i-C group starts with CXTC and has an F between the invariant S and QALGGH and ends with RXSHKKXX. The 2i-D subgroup starts with CXVC and ends with SYQALGGHKXSHRXXX. Finally, the 2i-X group starts with CKXC and ends with SHQALGGHRXSHKKVK. All the subgroups have an R after the QALGGH except for the 2i-D group. Similar specific intra-motif sequences can be observed in the second zinc finger motif (Fig. 4).

The spacing of the fingers is believed to be important to the DNA motifs they bind, and unlike ZFPs found in other organisms, is variable in plants [26]. For example, the 2iA group had ~ 19 amino acids between zinc fingers, 2i-B between 21 and 26, 2i-C 39–52, 2i-D 33–44 and 2i-X 39–43 amino acids between the zinc fingers (see Additional file 2). The 2i-X proteins all contained a string of “E”s in the spacer region between zinc fingers (see Additional file 2).

Nuclear localization sequences (NLS) were also predicted in the 146 ZFPs using NLSMapper http://nls-mapper.iab.keio.ac.jp/cgi-bin/NLS_Mapper_form.cgi (see Additional file 2). The NLS motifs were identified in different positions in the different groups of ZFPs. The 2i-A proteins had the NLS motif within the first zinc finger, 2i-B did not contain a NLS domain as defined by NLS mapper, 2i-C and 2i-D contained a NLS N terminal of the L box and 2i-X contained an NLS at the end of the zinc finger spacer (see Additional file 2).

The L-box is far less conserved, but contains 3–4 negatively charged amino acids D or E followed by amino acids with nonpolar neutral side chains such as ACLMI (hence the name L-box) as well as polar neutral side chains such as NST. The subgroups follow this general pattern, but there is little consensus within the groups (Fig. 4). Generally, the composite EAR motif contains DLNL. The 2i-A and 2i-C groups both contain the sequence LDLNL with a TP at the end for 2i-A and PA at the end for 2i-C. The 2i-B group has little consensus with variants at each amino acid. The 2i-D group contains DLN but none of the other amino acids are invariant. The 2i-X group contains LDLXLXC. Kagale and Rozwadowski [27] examined EAR motifs in plant species and defined LxLxL and DLNxxP as the most common types. The Brassica species in 2i-A and C match the LxLxL group while the 2i-D group conform to the DLN type EAR motif, but only a subgroup contains DLNxxP (see Additional file 2).

A manuscript published in 2015 outlines the Q-type ZFPs in *Brassica oleracea* [24] using the Bolbase genome, which was the only genome available at the time http://www.ocri-genomics.org/bolbase/ [20]. Only 25 Q-type ZFPs were identified and the protein sequences fell into two groups G1 and G2. About the same time, another manuscript was published by Parkin et al. [28] analyzing the transcriptome and methylome of *B. oleracea* and the data made available to Ensembl Plants (https://plants.ensembl.org/Brassica_oleracea/Info/Annotation/). Using this additional dataset 12 extra ZFPs were identified and used in this analysis of Brassica ZFPs. The G1 group contains mostly 2i-C type ZFPs with only one 2i-B and one 2i-A. The G2 group contains only 2i-D ZFPs. Therefore, the G1 group is biased towards 2i-C motifs. Therefore the additional dataset enhances the number of 2i-A and 2i-B type BoZFPs.

### Expression of Brassica *oleracea* and *napus* transcripts

The NCBI SRA (sequence read archive) database, https://www.ncbi.nlm.nih.gov/sra, was queried to confirm that the *B. oleracea* genes were indeed transcribed (Table 3). Examination of four BioProjects using either *B. oleracea var. capitata or italica* containing a total of 22 different samples provide evidence that transcript for 35 of 37 Q-type C2H2 ZFPs were present in at least one tissue type. Only BoZFP1 and BoZFP15 transcription could not be verified. Considering that these genes are often induced by stress, perhaps there is no experiment in the NCBI SRA database yet that uses the appropriate conditions to induce their expression.

**Table 3:** *B. oleracea* Q-type C2H2 TF transcript length and expression

BoZFP	Ensembl^a^	Bolbase^a^	Transcription Notes^b,c^
BoZFP1			cannot find transcript info for this gene
BoZFP2	534		found EAR motif in club root SRR but there is a gap between 112-132
BoZFP3		669	locus_18655, bud SRR630928 1–643 100% ID, seedling 102043,7–637, SNPs at 207, 537 and 541
BoZFP4		1170	bud and flower, missing 1–47 in bud with gap at 793–815, or 1–168 in flower up to 1100
BoZFP5		786	locus_27313, 2–746 with gap at 273–277
BoZFP6		807	locus_29791 in seedling 102043, 5–775
BoZFP7		1050	bud missing 1–66 bases, SNP at 372
BoZFP8	474		102043 flower has most of the transcript seedling is missing L-box
BoZFP9		708	locus_24740 and 40724, seedling 102043, 10 SNPs, 13–677
BoZFP10		831	locus_9351 stem also found in stem of 2, 1–824
BoZFP11		1092	flower with gap at 344–357
BoZFP12	477	420	flower
BoZFP13		807	107140 leaf 100% ID, flower of 2 is 1–763 and bud is 7–772 with a gap 672–683
BoZFP14		726	locus_2060, seedling 107140, 1–718 SNP at 492, in 102043 find gap at ~ 450–550
BoZFP15	615		cannot find this transcript
BoZFP16		702	seedling 102043, 2–670
BoZFP17	543		102043 seedling
BoZFP18	453		102043 complete coverage of entire transcript
BoZFP19		882	leaf of 107140 and flower of 2 covering 1–878
BoZFP20	864		102043 flower 2–841 includes the EAR motif
BoZFP21	447		102043 seedling 3–447 only 1 SNP found
BoZFP22	540		102043 seedling
BoZFP23		717	C1220 clubroot resistant, 1–717 with gap 327–352 and SNPs 173, 213, 471, 503, 653. 1–400 bases no gap or SNPs in 7 day cotyledon of broccoli
BoZFP24	792		bud of SRR630928
BoZFP25		690	seedling 102043, 14–684
BoZFP26	810	801	locus_47213, seedling 102043
BoZFP27	1155	936	clubroot resistant 27–862, gap 604–639
BoZFP28	507		107140 seedling
BoZFP29		696	seedling 102043
BoZFP30		570	flower
BoZFP31	639	468	flower M? Does not seem to coincide with map
BoZFP32	777		96–618, 635–750 of 777 in bud does have 2ZFPs and EAR motif maybe even L-box.
BoZFP33		696	locus_45812, stem 12–696 100% ID, leaf of 107140, 1–672
BoZFP34	486		102043 seedling great coverage 100% 3–480 of 486
BoZFP35		1038	locus_43474 in bud 6–971 100% ID SRR630928, 102043 seedling, 1–893, 17 SNPs and a 3 base gap
BoZFP36	708	720	locus_43800, leaf
BoZFP37		798	locus_ 26577, seedling 102043, 5–794

^a^Length of transcript in Ensembl Plants http://plants.ensembl.org/Brassica_oleracea/Info/Index

or Bolbase http://www.ocri-genomics.org/bolbase/genes.htm

^b^SRA (sequence read archive) data on the *B. oleracea* transcriptome 1) SRP032830; 9d old seedlings of the cabbage cultivars 102043 and 107140. Flowers were isolated from 102043 and leaf and root tissue were isolated from 107140. Loci were identified for 35,274 genes and presented in Kim et al. [34] Additional file 1: Table S1. 2) SRP017530; 7 week old plants isolated 7 different organs. 3) SRP029141; line C1220 clubroot disease resistant cabbage. 4) SRP034015; germinating broccoli

^c^locus # from Kim et al. [34] Additional file 1: Table S1, organ and/or cultivar and sequence ID = 100% unless noted otherwise

*B. napus* contains two times the number of Q-type ZFPs in comparison with *B. oleracea* and *B. rapa.* Considering the similarity to the genes in the diploid genomes, expression of these genes in *B. napus* was determined using previously published RNA-Seq data (see Additional file 4). *B. napus* genes were divided into groups based on the similarity to the18 Arabidopsis two fingered ZFPs. Given how similar the protein paralogs can be, the transcripts were aligned and the SRA results were mapped onto the alignments, so that differences between paralogs could be easily identified and the unique transcript verified. The reads for the SRA experiments were typically 101 nucleotides and generally abundant enough to identify unique differences between the different genes (see Additional file 4). The transcript alignments begin at the predicted start site and end at the predicted stop codon. Different groups of ZFPs had different size transcripts with 2i-A and B genes containing 447 to 615 nucleotides and 2i-C 798 to 1155 bases. The differences between paralogs varied from four to seven substitutions in Zat17-like transcripts to 31 to 130 in Zat5-like genes (see Additional file 4). Generally, SRA coverage could be found in the roots, leaves or flower buds (see Additional file 4 Table 1). Three genes BnaAZFP7, BnaCZFP12 and BnaCZFP17 did not have adequate SRA coverage and therefore it is unclear whether these genes are expressed. Only one of four 2i-X genes (BnaCZFP33) could be found in the SRA data examined. At this time, the expression of 2i-X *B. napus* genes BnaCZFP20, BnaCZFP21 and BnaAZFP31 could not be confirmed. Consequently, the SRA data confirmed that 68 unique *B. napus* ZFPs were expressed. The SRA data also confirmed that the “introns” predicted in many of the 2i-C genes were retained in the transcript leading to a larger protein than predicted in the database.

## Conclusions

While there are 18 2i ZFPs in Arabidopsis, there are 37 in *B. oleracea*, 35 in *rapa* and 74 in *napus*. Given that a whole genome duplication occurred in the Brassica genome ~ 4 million years after its split from the Arabidopsis progenitor, the twofold increase in these ZFPs in diploid Brassicas reflects the whole genome duplication [14]. The *B. oleracea* and *rapa* genomes also fused to form *B. napus* ~ 7500 years ago, and this resulted in doubling again the number of ZFPs [18]. Remarkably, most of these *B. napus* ZFPs are transcribed (see Additional file 4). Generally, this increase in genes creates an imbalance in gene expression and causes, over time, a reduction in expression of the excess genes. Whether these additional ZFPs have unique niches in expression or interactions with other proteins has yet to be determined.

The two fingered ZFPS of Arabidopsis fall into five groups based on their protein sequences (2i-A-D with outlier X) [3]. Aside from protein sequence similarity it is unclear whether these different phylogenetic groups play a different role in expression or interaction with different proteins. Most ZFPs have been studied one gene at a time and one group does not seem to fall into any specific expression category. ZFPs are often induced by a stress (biotic and/or abiotic) and due to the EAR motif, act to negatively regulate genes that they bind. ZFPs may modulate the stress response by binding activator transcription factors via the zinc fingers while the EAR motif binds to bridging proteins such as TPL or TPL-like proteins in Arabidopsis that subsequently interacts with HDAC proteins such as HDA19. This interaction results in tightening of the DNA region to which the zinc fingers bind [27, 29]. Ectopic expression of ZFPs can lead to an increase in tolerance to specific stresses [5]. In addition, three amino acid changes in the EAR domain of the Arabidopsis ZFP, ZAT7, results in plants with lower salinity tolerance [30]. Thus, the EAR motif is integral to the enhanced tolerance. How the over-expression of a negative regulator results in increased tolerance is also not apparent. OE-Zat18 a 2i-A member enhances drought tolerance [31]. Several other 2i groups of ZFPs enhance salt tolerance when over-expressed such as Zat7 (2i-B) Zat10 and Zat6 (2i-D) [30, 32, 33]. Thus the protein sequence phylogeny of the ZFP may not predict the effect it might have on stress tolerance.

This work identifies ZFPs from three genomes that are related but have diverged over evolutionary time. It is remarkable that the *B. napus* genome appears to maintain expression of most of the ZFPs from *B. oleracea* and *B. rapa*. Identifying the expression pattern of the homologs in the different species may identify niches unique to the different ZFPs. This work is only a beginning, outlining what variation in two fingered Q-type ZFPs exists in these species. Considering the evolution of these species, it seems remarkable that given the minor differences found within the ZFP proteins of the individual Brassica diploids, RNA-Seq data confirms at least 68 of the *B. napus* genes are expressed. Given the improvements in tolerance to stresses previously identified in ZFPs over-expressed in Arabidopsis [5], manipulation of the Brassica ZFPs may improve stress tolerance in these important agronomic crops.

## Additional files

Additional file 1: **Table** **S1-S4**, **Figure S1.** Brassica gene names, location on genome, comparative percent identity of protein sequences. Protein sequences used in this work that have been altered from the appropriate databases. (PDF 201 kb)

Additional file 2: Alignment of Q-type-ZFP proteins from *Brassica oleracea*, *B. rapa* and *B. napus* by group. (PDF 79 kb)

Additional file 3: Complete phylogenetic tree of 146 *Brassica* ZFPs and 18 Arabidopsis ZFPs form 5 groups. (PDF 692 kb)

Additional file 4: SRA data confirming expression of *B. napus* ZFPs (PDF 195 kb)

## Abbreviations

BnaA: *Brassica napus* A genome
BnaC: *Brassica napus* C genome
Bo: *Brassica oleracea*
Br: *Brassica rapa*
EAR: **e**thylene-**r**esponsive element-binding **f**actor (ERF)-associated **a**mphiphilic **r**epression
Mya: million years ago
NCBI: National Center for Biotechnology Information
SRA: Sequence read archive
ZFP: Q-type C2H2 zinc finger proteins
