# Supplementary material for: Comparative analysis of the genetic variability within the Q-type C2H2 zinc-finger transcription factors in the economically important cabbage, canola and Chinese cabbage genomes
Source: Hereditas. 2018 Sep 21;155:29. doi: 10.1186/s41065-018-0065-5 (PMC6150991; doi:10.1186/s41065-018-0065-5)
Supplement: Supplementary file 1 — Table S1-S4, Figure S1. Brassica gene names, location on genome, comparative percent identity of protein sequences. Protein sequences used in this work that have been altered from the appropriate databases. (PDF 201 kb) [file 41065_2018_65_MOESM1_ESM.pdf]

Additional file 1 Brassica gene names, location on genome, comparative percent identity of protein sequences. Protein sequences used in this work that have been altered from the appropriate databases

Title; Comparative analysis of the genetic variability within the Q-type C2H2 zinc-finger transcription factors in the economically important cabbage, canola and Chinese cabbage genomes

Table S1 *Brassica oleracea* Q-type C2H2 TF gene name and location p2
Table S2 Identity of BnaC genome genes, percent identity to *B. oleracea* proteins and map position p3
Table S3 Identity of BnaA genes, percent identity to *B. rapa* proteins and map position p4
Table S4 Percent Identity of protein sequences of *B. oleracea* and *B. rapa* ZFPs and *B. rapa* gene location p5
Fig S1 Protein sequences altered from the database and given the prefix My or suffix mod p6-9

Table S1 *Brassica oleracea* Q-type C2H2 TF gene name and location

BoZFP ID*	Bo (Ensembl) gene ID**	Map Location***	Length aa	Bolbase gene ID****	Length aa
**BoZFP1**	Bo00576s170.mod	Scaffold00576: 193,471-194,121	170		
**BoZFP2**	Bo1g067420.1	C1: 20,123,967-20,124,712	177		
**BoZFP3**	Bo1g081350.1	C1: 23,978,440-23,979,374	222	Bol021981	222
**BoZFP4**	Bo1g143860.1	C1: 40,822,619-40,824,277	394	Bol023133	389
**BoZFP5**	Bo1g113530.1	C1: 34,409,616-34,410,715	261	Bol031023	261
**BoZFP6**	Bo2g004110.1	C2: 687,466-688,594	268	Bol005441	268
**BoZFP7**	Bo2g006530.1	C2: 919,077-920,546	349	Bol024437	349
**BoZFP8**	Bo2g025350.1	C2: 7,603,600-7,604,261	157		
**BoZFP9**	Bo2g168030.1	C2: 52,684,903-52,685,892	235	Bol008081	235
**BoZFP10**	Bo3g002370.1	C3: 634,263-635,425	276	Bol015320	276
**BoZFP11**	Bo3g002780.1	C3: 857,341-858,868	363	Bol015360	363
**BoZFP12**	Bo3g017740.1	C3: 5,814,134-5,814,800	158	Bol025914	139
**BoZFP13**	Bo3g073320.1	C3: 28,302,837-28,303,965	268	Bol026699	268
**BoZFP14**	Bo3g096410.1	C3: 34,838,200-34,839,215	241	Bol004311	241
**BoZFP15**	Bo3g130310.1	C3: 47,777,238-47,778,098	204		
**BoZFP16**	Bo3g142840.1	C3: 51,274,351-51,275,332	233	Bol008452	233
**BoZFP17**	Bo4g030570.1mod	C4: 6,709,405-6,710,163	180		
**BoZFP18**	Bo4g069230.1	C4: 14,628,085-14,628,717	150		
**BoZFP19**	Bo4g071820.1	C4: 15,391,224-15,392,457	293	Bol013409	293
**BoZFP20**	Bo4g165160.1	C4: 44,311,690-44,312,897	287		
**BoZFP21**	Bo4g166690.1	C4: 44,764,101-44,764,725	148		
**BoZFP22**	Bo4g186920.1	C4: 49,744,052-49,744,807	179		
**BoZFP23**	Bo4g187280.1mod	C4: 49,997,612-50,002,507	239	Bol025266	238
**BoZFP24**	Bo5g001300.1	C5: 174,047-174,838	263		
**BoZFP25**	Bo5g052290.1	C5: 17,444,490-17,445,455	229	Bol002087	229
**BoZFP26**	Bo5g113850.1	C5: 34,947,274-34,948,407	269	Bol018113	266
**BoZFP27**	Bo5g137370.1	C5: 42,791,493-42,793,109	384	Bol005838	311
**BoZFP28**	Bo6g067580.1	C6: 18,954,011-18,954,719	168		
**BoZFP29**	Bo7g048650.1	C7: 18,072,481-18,073,454	231	Bol041794	231
**BoZFP30**	Bo7g065200.1	C7: 24,819,907-24,820,704	189	Bol027136	189
**BoZFP31**	Bo7g099140.1mod	C7: 38,865,282-38,866,174	212	Bol016976	155
**BoZFP32**	Bo8g118200.1	C8: 41,661,803-41,662,889	258		
**BoZFP33**	Bo9g019120.1	C9: 6,116,920-6,117,893	231	Bol005710	231
**BoZFP34**	Bo9g140820.1	C9: 42,772,874-42,773,553	161		
**BoZFP35**	Bo9g180060.1	C9: 53,365,852-53,367,308	346	Bol002432	345
**BoZFP36**	Bo9g181170.1	C9: 53,439,957-53,440,946	235	Bol002425	239
**BoZFP37**	Bo9g181940.1	C9: 53,886,586-53,887,701	265	Bol010048	265

*BoZFP# this manuscript; **Gene name in http://plants.ensembl.org/Brassica_oleracea/Info/Index, mod refers to change in annotation in this work; ***Map location from Ensembl with protein length in amino acids; **** http://www.ocri-genomics.org/bolbase/gene name and length in amino acids.

Table S2 Identity of BnaC genome genes, percent identity to *B. oleracea* proteins and map position

**BoZFP#**	**BnaC**	**BnaC ID***	**map position**
**BoZFP2**	BnaCZFP1	BnaC01g24440D (99%)	chrC01:19,230,575..19231108
**BoZFP3**	BnaCZFP2	BnaC01g26360D(99%)	chrC01:23,231,072..23,231,964
**BoZFP3**	BnaCZFP3	BnaC01g26370D(96%)	chrC01:23,238,238..23,238,915
**BoZFP5**	BnaCZFP4	BnaC01g33270D(100%)	C01:32,451,265..32,452,050
**BoZFP6**	BnaCZFP5	BnaC02g03060D(99%)	chrC02:1,427,204..1,428,076
**BoZFP9**	BnaCZFP6	MyBnaC02g16730D(100%)**	chrC02:12,257,930..12,258,637
**BoZFP11**	BnaCZFP7	MyBnaC03g71210D(99%)**	chrC03_random:30,762..31,850
**BoZFP10**	BnaCZFP8	MyBnaC03g01240D(99%)**	chrC03:560,518..561,348
**BoZFP13**	BnaCZFP9	BnaC03g73930D(100%)	chrC03_random:1,920,760..1,921,566
**BoZFP12**	BnaCZFP10	BnaC03g11570D (99%)	chrC03:5,629,713..5,630,189
**BoZFP14**	BnaCZFP11	BnaC03g48520D(100%)	chrC03:33,505,767..33,506,826
**BoZFP15**	BnaCZFP12	BnaC03g55590D (99%)	chrC03:44,620,169..44,620,783
**BoZFP16**	BnaCZFP13	BnaC03g58080D(99%)	chrC03:47,491,421..47,492,339
**BoZFP17**	BnaCZFP14	BnaC04g07880D(99%)	chrC04:5,930,766..5,931,308
**BoZFP18**	BnaCZFP15	BnaC04g15490D (100%)	chrC04:13,278,951..13,279,403
**BoZFP19**	BnaCZFP16	MyBnaC04g16100D(98%)**	chrC04:14,096,933..14,098,086
**BoZFP1**	BnaCZFP17	BnaC04g27120D(98%)	chrC04:28,353,782..28,354,285
**BoZFP21**	BnaCZFP18	BnaC04g39930D (100%)	chrC04:40,955,101..40,955,547
**BoZFP22**	BnaCZFP19	BnaC04g45160D(98%)	chrC04:44,926,045..44,926,584
**BoZFP23**	BnaCZFP20	MyBnaC04g45560D(98%) 244/239	chrC04:45,198,908..45,202,908
**BoZFP24**	BnaCZFP21	BnaC05g00100D(95%) 263/260	chrC05:30,570..31,371
**BoZFP25**	BnaCZFP22	BnaC05g21480D(100%)	chrC05:15,206,540..15,207,473
**BoZFP26**	BnaCZFP23	BnaC05g33170D(98%) 266/269	chrC05:32,662,677..32,663,477
**BoZFP27**	BnaCZFP24	MyBnaC05g42550D(100%)**	chrC05:39,854,843..39,855,997
**BoZFP28**	BnaCZFP25	BnaC06g14860D(99%)	chrC06:17,597,230..17,597,736
**BoZFP29**	BnaCZFP26	BnaC07g11700D(100%)	chrC07:17,224,872..17,225,567
**BoZFP30**	BnaCZFP27	BnaC07g16940D(99%)	chrC07:22,965,116..22,965,685
**BoZFP31**	BnaCZFP28	MyBnaC07g31600D(93%) 202/212**	chrC07:35,616,985..35,617,748
**BoZFP33**	BnaCZFP29	MyBnaC09g07240D(99%)**	chrC09:4,487,403..4,488,429
**BoZFP34**	BnaCZFP30	BnaC09g35160D (100%)	chrC09:38,588,256..38,588,781
**BoZFP35**	BnaCZFP31	MyBnaC09g50520D(99%)**	chrC09:48,326,153..48,327,193
**BoZFP20**	BnaCZFP32	MyBnaCnng66450D(100%)**	
**BoZFP32**	BnaCZFP33	BnaCnng09030D(99%)	
**BoZFP36**	BnaCZFP34	MyBnaCnng20570D(97%)**	
**BoZFP37**	BnaCZFP35	BnaCnng02770D(97%)	
**BoZFP22**	BnaCZFP36	BnaCnng48130D(80%)	

*BnaC ID from http://www.genoscope.cns.fr/index.html, with percent identity of protein sequences **Genes with a My suffix were modified to take out introns that when present resulted in shorter protein sequences.

Table S3 Identity of BnaA genes, percent identity to *B. rapa* proteins and map position

BrZFP ID	BnaA ID	BnaA*(%ID)	gene location
**BrZFP1**	BnaAZFP1	BnaA01g21030D((98%)	chrA01:12,934,674..12,935,342
**BrZFP2**	BnaAZFP2	BnaA01g26030D(98%)	chrA01:18,144,395..18,145,189
**BrZFP3**	BnaAZFP3	BnaA01g31690D(100%)	chrA01:21,499,602..21,500,438
**BrZFP6**	BnaAZFP4	BnaA02g06780D(100%)	chrA02:3,227,932..3,228,405
**BrZFP6**	BnaAZFP5	BnaA02g06790D(94%)	chrA02:3,233,537..3,234,019
**BrZFP8**	BnaAZFP6	MyBnaA03g00910D(100%)**	chrA03:392,772..393,602
**BrZFP9**	BnaAZFP7	MyBnaA03g01240D(100%)**	chrA03:587,232..588,311
**BrZFP10+BrZFP11**	BnaAZFP8	BnaA03g09250D(100%)	chrA03:4,154,835..4,155,314
**BrZFP17**	BnaAZFP9	BnaA03g17210D(81%)	chrA03:8,070,393..8,070,938
**BrZFP12**	BnaAZFP10	BnaA03g35310D(97%)	chrA03:17,244,857..17,245,790
**BrZFP13**	BnaAZFP11	MyBnaA03g40650D(98%)**	A03:20,315,618..20,316,444
**BrZFP35**	BnaAZFP12	MyBnaA03g46860D(96%)**	chrA03:24,040,169..24,040,966
**BrZFP14**	BnaAZFP13	BnaA04g04630D9100)	chrA04:3,403,309..3,403,833
**BrZFP15**	BnaAZFP14	MyBnaA04g16320D(97%)**	chrA04:13,313,188..13,314,051
**BrZFP16**	BnaAZFP15	BnaA04g16610D(100%)	chrA04:13,585,217..13,585,663
**BrZFP17**	BnaAZFP16	BnaA04g21410D(100%)	chrA04:16,317,949..16,318,491
**BrZFP19**	BnaAZFP17	BnaA05g07070D(100%)	chrA05:3,826,091..3,827,426
**BrZFP20**	BnaAZFP18	BnaA05g20490D(97%)	chrA05:15,859,357..15,860,209
**BrZFP18**	BnaAZFP19	MyBnaA05g28380D(100%)**	chrA05:20,160,901..20,162,055
**BrZFP21**	BnaAZFP20	BnaA06g18210D(96%)	chrA06:10,460,380..10,460,958
**BrZFP22**	BnaAZFP21	BnaA06g19880D(56%)	chrA06:12,495,829..12,496,338
**BrZFP22**	BnaAZFP22	BnaA06g19890D(98%)	chrA06:12,499,744..12,500,256
**BrZFP23**	BnaAZFP23	BnaA06g25060D(97%)	chrA06:17,317,153..17,318,213
**BrZFP24**	BnaAZFP24	BnaA06g36830D(97%)	chrA06:24,067,503..24,068,060
**BrZFP27**	BnaAZFP25	BnaA07g37620D(100%)	chrA07_random:1,206,391..1,206,897
**BrZFP25**	BnaAZFP26	MyBnaA07g13700D(100%)**	chrA07:12,076,971..12,077,837
**BrZFP26**	BnaAZFP27	BnaA07g14080D(99%)	chrA07:12,518,689..12,519,141
**BrZFP28**	BnaAZFP28	BnaA08g18790D(100%)	chrA08:14,612,308..14,613,009
**BrZFP29**	BnaAZFP29	MyBnaA09g07340D(99%)**	chrA09:3,628,571..3,629,597
**BrZFP30**	BnaAZFP30	BnaA09g27780D(99%)	chrA09:20,844,403..20,845,092
**BrZFP32**	BnaAZFP31	MyBnaA10g00100D(99%)**	chrA10:41,463..45,039
**BrZFP33**	BnaAZFP32	BnaA10g12780D(99%)	chrA10:10,438,137..10,438,619
**BrZFP34**	BnaAZFP33	MyBnaA10g25770D(100%)**	chrA10:16,525,647..16,526,684
**BoZFP36***	BnaAZFP34	Bna10g25850D(85%) 228/235	chrA10:16,573,008..16,573,694
**BrZFP35**	BnaAZFP35	BnaA10g26510D(97%)	chrA10:16,897,739..16,898,536
**BrZFP4**	BnaAZFP36	BnaAnng00680D(99%)	
**BrZFP5**	BnaAZFP37	MyBnaAnng17660D(97%)**	
**BrZFP7**	BnaAZFP38	MyBnaAnng05120D(100%)**	

*BnaA ID from http://www.genoscope.cns.fr/index.html percent identity of protein sequences **Genes with a My suffix were modified to take out introns that when present resulted in shorter protein sequences.

Table S4 Percent Identity of protein sequences of *B. oleracea* and *B. rapa* ZFPs and *B. rapa* gene location

BoZFP ID	BrZFP ID	Br (%ID to Bo)*	gene location
**BoZFP3**	BrZFP1	Bra029985 (95%)	A01:15491197-15491862
**BoZFP5**	BrZFP2	Bra038219 (95%)	A01:21530778-21531572
**BoZFP4**	BrZFP3	MyBra034116 (92%)**	A01:27374140-27374976
**BoZFP6**	BrZFP4	Bra028831 (94%)	A02:1842245-1843051
**BoZFP7**	BrZFP5	Bra028791 (93%)	A02:2036456-2037514
**BoZFP8**	BrZFP6	MyBra020284 (96%)**	A02: 6021571-6,021,898
**BoZFP9**	BrZFP7	Bra031834 (96%)	A02:27629364-27630071
**BoZFP10**	BrZFP8	Bra005766 (92%)	A03:493,275-494105
**BoZFP11**	BrZFP9	Bra005803 (96%)	A03:721802-722881
**BoZFP12**	BrZFP10<br>BrZFP11	Bra006691 +Bra006692 (97%)	A03:4,655,037-4655516 (6692)/<br>A03:4651954-4652433 (6691)
**BoZFP13**	BrZFP12	Bra001752 (94%)	A03:18,247,425-18248228
**BoZFP31**	BrZFP13	Bra012914 (95%)199 of 213	A03:21,533,367-21534008
**BoZFP1**	BrZFP14	Bra039806 (85%)	A04:3,756,644-3757168
**BoZFP20**	BrZFP15	Bra034396 (98%)	A04:12,327,159-12328037
**BoZFP21**	BrZFP16	Bra035678 (98%)	A04:12,897,626-12898072
**BoZFP22**	BrZFP17	Bra017188 (96%)	A04:16,121,469-16122011
**BoZFP17**	BrZFP19	MyBra005197 (94%)**	A05:2,020,403-22021483
**BoZFP26**	BrZFP20	Bra022436 (92%)	A05:3,959,262-3959765
**BoZFP27**	BrZFP18	MyBra029843 (96%)**	A05:17,704,957-17,705,964
**BoZFP15**	BrZFP21	Bra018225 (94%)	A06: 11,253,932-11254740
**BoZFP2**	BrZFP22	Bra019477 (75%)	A06:13,158,164-13158676
**BoZFP14**	BrZFP23	Bra024448 (93%)	A06:15,810,122-15810847
**BoZFP30**	BrZFP24	Bra033666 (93%)	A06:25,732,392-25732949
**BoZFP19**	BrZFP25	Bra011970 (95%)	A07:10,863,331-10864197
**BoZFP18**	BrZFP26	Bra011931(99%)	A07:11,243,542-11243994
**BoZFP28**	BrZFP27	Bra003148 (96%)	A07:11,582,403-11582909
**BoZFP16**	BrZFP28	Bra010922 (97%)	A08:17126059-17126760
**BoZFP33**	BrZFP29	Bra037845(94%)	A09:3,856,521-3857216
**BoZFP25**	BrZFP30	Bra032845 (97%)	A09:12,448,171-12448860
**BoZFP32**	BrZFP31	Bra032661 (98%)	A09:37,061,324-37062100
**BoZFP24**	BrZFP32	My Bra033220 (89%)**	A10:4,837,253-4841141
**BoZFP34**	BrZFP33	Bra002528 (94%)	A10:9,046,551-9047033
**BoZFP35**	BrZFP34	Bra009457 (96%)	A10:16,726,584-16727621
**BoZFP37**	BrZFP35	Bra009525 (96%)	A10:17,085,202-17085999

* Ensembl Plants gene ID. http://plants.ensembl.org/Brassica_rapa/Info/Index
** Suffix My refers to reannotated protein sequences
No Br orthologs to BoZFP23, 29 or 36

Fig S1
Brassica oleracea modified proteins
>BoZFP17(Bo4g030570.1mod)
MKRERSEFEESIKMSDDIARCLMILSQTSMVKQVDVNQYTDRDTSNRFECKTCNKRFSSF
QALGGHRASHKKPKLSVDQKEVKHVTNNYNGTHMHECSICGQSFGTGQALGGHMRRHRSS
MKVEPSQLISPVISNVPVLKRCSSSKRVLSLDLNLTPLENDLETIFGKTFFPNIDMKFVV
>BoZFP23(Bo4g187280.1mod)
MDSSYTDFVMFNSQGQHDEGNMSRLPWKRVRDEDEELANCLVLLSNSGNAYNNNEHGRIK
DKNVKKRKTGHVFQCKGCKKVFASHQALGGHRASHKKVKGCFASQDKKAEEEEEEEYKEE
EEEDEEEEEEEEPHITTRKRSNAHECTICHRVFSSGQALGGHKRCHWLTPSTNYLRMKPL
NDSSTHHHHSQPLDQPSLDLSLACVDPTVMTIGRDGGGNNHNATTSSNSWLKLASGDWS
>BoZFP31(Bo7g099140.1mod)
MALDTLNSPTSTTAPPPFLTKPENLESWTKRKRTKRHRTVDQSHPPSEEEYLAICLLMLA
RGSSSTNDGNDHHSPPAPPSDHDHRDYKCSVCGKSFPSYQALGGHKTSHRKPVSNINCQD
TINSGNGSVTNNGNISNGLIGQSGKTHKCSICFKSFPSGQALGGHKRCHYDGGNGNGSNN
HGFDLNLPADHDETLVRVNSPVKNQSRCHDYY

Brassica rapa modified proteins
> BrZFP3(MyBra034116)
METAEEAISAAKAQALII
KGKRTKRQRPQSPIPFSIVPPMSSQEPDTQEESTSLVAKEKSLNDEINYNNNKNDNNILS
IGVTSSSSTSSSFNNATLKAAADEEDQDMANCLILLAQGHSLPHQQPQTRQLMVSYQESG
NNNNNAYRSSSRRFLETSSSNGTTTSGGRAGYYVYQCKTCDRTFPSFQALGGHRASHKKP
KAAPGLHDLKKSIYNDAVSHHLNNVLTTTPNNNNNHRSLVVHGKANNNKVHECGICGAEF
TSGQALGGHMRRHRGAGVAATATPTATLALPASAATANTVLSLSPMSFDQLSDGPVYPVQ
APVKRARSAVVSLDLDLNLPAPEDENRVNGLSVASKQEHGHEQTQGREEQKSIVLSSAPT
LVDCYY
> BrZFP18(MyBra029843)
METAEEAISAAKEQALILKGKRTKRQRLQSPIPFSIVPPMSSQEPDVEEESTSL
VSKEKSLNDDINTNKNDNNVLINGVTSPASSSSNNNATLKTAADEEDQDMANCLILLAQG
HYTPQQQPQQTRQFMMSYQESGNNNNNAYRSSSRRFLETSSPNGTT
SGGRAGYYVYQCKTCDRTFPSFQALGGHRASHKKPKAAAGLHSDHDLKKSIYNDAVSLHL
NNVPTATPNNNSSHRSLVVYGKAGNNKVHECGICGAEFTSGQALGGHMRRHRGAVVASAA
SASTATVRVAATAGTANTALSLSPMSFDHMSVHPVQGPVKRARSAVVSLDLDLNLPAPED
VNRVNGLSFASKQEQEHEHEQTQQREEQKSIVLSSAPTLVDCHY
> BrZFP19(MyBra005197)
MKRERSEFEESIKMSDDIARCLMILSQTSMVKQVDVNQYTERDTSNRFECKTCNKRFSSFQALGGHRASHK
KPKLSVDQKEVKHVTNNYNGTHIHECSICSQSFGTGQALGGHMRRHRSSVTVEPLQTISPVNSTVPVLKRC
SSSKRVLSLDLNLTPLENDLETIFGKTFFPNIDMKFVV
> BrZFP32(MyBra033220)
MNLSCIDFVMFSSRGQHDEGNMSRPSWKRERSNNLINLSPNEDEELANCLVLLSNSGDHY
NSGGHHNKHGHGKGKSIKKQKTSQAFQCKACKKVFTSHQALGGHRESHKKVKGCFATQNK
EEEEDEDEYKEEDEEEEEEEEEDKATAADYNNIITRKRSNAHECTICHRVFSSGQALGGH
KRCHWLTPSSYFHMTPLHDSSSVARSQMLEQPSLDLNLTCQEYSVDPTVMSVWRDDGGNN
HNATSPDSWLKLASGDWS

Brassica napus modified proteins
> BnaCZFP6(MyBnaC02g16730D)
MALETLNSPSSATASARPLLRYREEMEPENLEQWAKRKRTKRQRFDQSRLDQETAPSEEE
YLALCLLMLARGSAVKSPLRPSPSSDHRGYKCTVCGKSFSSYQALGGHKTSHRKPASNVN
VPINQEPSNNSHSNSNGGSVVINGNGFSQSGKIHTCSICFKSFSSGQALGGHKRCHYDGG
NNGNGNGSSSNSVEVVGGSDGSYVDDERSSEQSATGDNRGFDLNLPADQVAVVIS
> BnaCZFP7(MyBnaC03g71210D)
MEAFEEAIAASKEQALILKGKRTKRQRPQSPIPFSVSPPIVEEEVSNVLDSKENDVANRK
KDGVVTSSSSSASWSSNNNPTLKGEEDEEDQDVANCLILLSQGHSFPQHNQQLKIPHQEI
NNNNTYRFSSRRFLETSSSNGGGKSGYYVYQCKTCDRTFPSFQALGGHRASHKKPRATSF

YSNLDLKKSIYENDAASLVTTTNIYNNNKNNNRSLVAYGKANNNKVHECGICGAEFTSGQ
ALGGHMRRHRGAVVVAAAPAPIVTVAAAAANTELSLSSMSYDQISDGQDHLVMPEAKKAK
KMVVSLDLDLNLPAPEDENRVNGLSLALKQKHEQEQEHQETKQKEEPVSLVLSAPTLVDC
YY
> BnaCZFP8(MyBnaC03g01240D)
MTSVHEETRLVLLIKGKRTKRQRSASPHIKAEVMSSVCNEERSLEAREEGAGEIEFRGAT
DEDQDMANCLMLLSQGHKSNTSGDPLLTQKIGFLSNKKPVASLGLGINGVYQCKTCDKSF
HSFQALGGHRTSHKKPKLGAIVLKCDEKKSASAVKTVEAARVVGSFLSLQVTSSDGSKKQ
EKTHECSICKAEFSSGQALGGHMRRHRGLTLNANANSTIRTATSSSHNQESIRENNFMEL
DLNLPAPEDEPKFVFASKDQIILFTAASNSLIDCHH
> BnaCZFP16(MyBnaC04g16100D)
MMGQDHEVGSDQTQIIKGKRTKRQRSSSSTFLVAAAATTITSTSSSADGERTASDEYNSVV
SSPVTTTDCTEEEEDMAICLIMLARGAAPSPPLPDLKNSTKTDKNLHLKTSSSENSSFYV
YECKTCNRTFPSFQALGGHRASHKKPRASIDEKAKVPLTQLKSSASEEGQKSHFKVSGPA
LASKASNIIISKANKVHECSICGSEFTSGQALGGHMRRHRTVTNVVSSPVSTAEVSRNST
EEETENLSRSMEQRKYLPLDLNLPAPEDDLRESKFQRIVFSATTPALIDCHY
> BnaCZFP20(MyBnaC04g45560D)
MDSSYTDFVMFNSQGQHDEGNMSRLPWKRVRDEDEELANCLVLLSNSGNAYNNNEHGHIK
DKNVKKRKTGHVFQCKGCKKVFASHQALGGHRASHKKVKGCFASQDKKAEEEEEEEYEEE
EEEEEEEEEDKPHITTRKRSNAHECTICHRVFSSGQALGGHKRCHWLTPSTNYLRMKPLN
DSSTHHHHSQPLDQPSLDLSLACVDPTVMTIGRDGGGNNHNATTSSNSWLKLASGDWS
> BnaCZFP24(MyBnaC05g42550D)
METAEEAISAAKEQALILKGKRTKRQRLQSPIPFSIVPPMSSQEPDVEDESTSLVSKEKS
LNDEINTNKNDNNMLSNGVTSPASSSSNNNATLKTAADEEDQDMANCLILLAQGHYTPQQ
QPQQTRQFMMSYQESGNNNNNAYRSSSRRFLETSNGTTSGGRAGYYVYQCKTCDRTFPSF
QALGGHRASHKKPKAAAGLHSDHDLKKSIYNDAVSLHLNNVPAATPNNNSSHRSLVVYGK
ANNNKVHECGICGAEFTSGQALGGHMRRHRGAVVASAASASTATVRVAATAGTANTALSL
SPMSFDQMSVHPVQAPVKRARSAVVSLDLDLNLPASEDVNRVNGLSFASKQEQEQEHEHE
QTHQREEQKTLVLSSAPTLVDCHY
> BnaCZFP28(MyBnaC07g31600D)
MALDTLNSPTSTTAPPPFLTKPENLESWTKRKRTKRHRTVDQSHPPSEEEYLAICLLMLA
RGSSSTNDGNDHPSPPAPPSDHHHRDYKCSVCGKSFPSYQALGGHKTSHRKPVSNTNCQD
TINTGNGSVTNNGNITHKCSICFKSFPSGQALGGHKRCHYDGGNGNGSNNHGFDLNLPAD
HDETLVRVNSPVKNQSRCRDYY
> BnaCZFP29(MyBnaC09g07240D)
MALETLNSPTSATAAARPFLRYREEMEPENLEQWAKRKRTKRQRFDHNQEKTPSEEEYLA
LCLLMLARGSTVKSPPPPSLPSSDHRGYKCTVCGKSFSSYQALGGHKTSHRKPVNTNNGP
SAQEPSNKTHGNSNGGSIVINGNGVSQSGKTHTCSICFKSFSSGQALGGHKRCHYDGGNN
GNGSSSNSVEVIGGSDVSDVDDERSSEQSAIGGHRGFDLNLPADQVTVVIS
> BnaCZFP31(MyBnaC09g50520D)
MEAFEEAIAASKEQSLIFKGKRTKRQRPQSPIPFSIISPPIVSSHAHDIQEESKKDGVIT
SSSSSASWSSNNNATLKAEEDEEEQEIANCLILLSQGHSLPLPNHEANNNNTYRFSSRRF
LETSSSNGGDKAGYYVYQCKTCDRTFPSFQALGGHRASHKKPKATLSSYSNIDVKKNIYE
SNAVSLVTTSTIYKNNNNRSLAVYGKAGSNKVHECGVCGAEFTSGQALGGHMRRHRGAVV
IAAAPVTTVTVATAAANTELSLSSMSFDQISDGQDHLVMPATKRAKKTVVSLDLDLNLPA
PEDENRVNGFTFALKQKQEQEHQPTMQREEPKCLHMSAPTLVDCHY
> BnaCZFP32(MyBnaCnng66450D)
MMSQDHVGSDQTQIIKGKRTKRQRSSSSTFLVAAAAATTNTSSN
SSAGDGGGGRAVSDEYNSAVSSPVTTTDCTEEEEDMAICLIMLARGAALSPDLKNSRKAD
KTLSSAENSSFFVYECKTCSRTFSSFQALGGHRASHKKPRVSIEEKTKLPLMQAKSSGSE
EGQKYNFKVFGSSLASLSSNIIISKANKVHECSICGSEFTSGQALGGHMRRHRTATTAVI
PVATTEVSRNSTEEETENLSSYIEQRKYLPLDLNLPAPEDDLRESKFQGIVFSTTPALID
CHY
> BnaCZFP34(MyBnaCnng20570D)
MALEALSSPRLASPVPTLFQDYAVGFHGSKGKRSKRSRSEFDRSLTEDEYIALCLMLLAR
DGNRTRDLPSCSSLPPLLPTPTSTHTHKCSVCDKAFSSYQALGGHKASHRKKSSQTQSSG

GDEKSTSSAITIVRHGGGSVKPHVCSICNKSFATGQALGGHKRCHYEGKNGGGGSSSVSI
SEGVGSTSHVSSGSHHHHHHHHRGFDLNIPPIPAFWTVNGEEEVMSPMPTKKLRLE

>BnaAZFP3(MyBnaA01g31690D)
METAEEAISAAKAQALIIKGKRTKRQRPQSPIP
FSIVPPMSSQEPDTQEESTSLVAKEKSLNDEINYNNNKNDNNILSIGVTSSSSTSSSFNN
ATLKAAADEEDQDMANCLILLAQGHSLPHQQPQTRQLMVSYQESGNNNNNAYRSSSRRFL
ETSSSNGTTTSGGRAGYYVYQCKTCDRTFPSFQALGGHRASHKKPKAAPGLHDLKKSIYN
DAVSHHLNNVLTTTPNNNNNHRSLVVHGKANNNKVHECGICGAEFTSGQALGGHMRRHRG
AGVAATATPTATLALPASAATANTVLSLSPMSFDQLSDGPVYPVQAPVKRARSAVVSLDL
DLNLPAPEDENRVNGLSVASKQEHGHEQTQGREEQKSIVLSSAPTLVDCYY
> BnaAZFP6(MyBnaA03g00910D)
MTSVHEETRLVLLIKGKRTKRQRSVSPHMNAEAMSSVCKEERSLEAREGVGEIEFRGATD
EDQDMANCLMLLSQGHKSNTSGDPLLTQKIGFLSNKKPVASLGLGLNGVYQCKTCDKSFH
SFQALGGHRTSHKKPKLGAIILKCGEKKSASAVKTVEAARVVGSFLSLQVTSSEGTKKQE
KTHECSICKAEFSSGQALGGHMRRHRGLTLNANANSTTRTEISSSHHQESIREKNFIELD
LNLPAPEDEPKFVFASKDQILLFAAAASNSLIDCHH
> BnaAZFP7(MyBnaA03g01240D)
MEAFEEAIAASKEQALILKGKRTKRQRPQSPIPFSVSPPIVEEEVSNVLDSKENDVANRK
KDGVITSSSSSASWSSNNNPTLKAEEDEEDQDIANCLILLSQGHSFPQHNQQLKIPHQEI
NNNNTYRFSSRRFLETSSSNGGGKSGYYVYQCKTCDRTFPSFQALGGHRASHKKPRATSF
YSNLDVKKSIYENDAASLTNIYNNKNNNNRSLVAYGKAGNNKVHECGICGAEFTSGQALG
GHMRRHRGAVVVAAAPAPIVTVAAAAANTELSLSSMSYDQISEGQDHLVMPEAKKAKKMV
VSLDLDLNLPAPEDENRVNGLSLTLKQKHEQEQEHQQTKQREEQVSLVLSAPTLVDCYY
> BnaAZFP11(MyBnaA03g40650D)
MALDTLNSPTSTTAPPPFLTEPENLESWTKRKRTKRHRTVDQSHPPSEEEYLAICLLMLA
RGSSSSSSNDGNDHHSPPAPPSDHHHRDYKCSVCGKSFPSYQALGGHKTSHRKPVSNTNC
QDTNNSANGSVTNNGNISNGLISQSGKTHKCSICFKSFPSGQALGGHKRCHYDGGNGNGS
NNHGFDLNLPADHDETLVKC
> BnaAZFP12(MyBnaA03g46860D)
MTSVHEETRLVLLVKGKRTKRQRSASPHMNAEAVSGVCSEEPSLEAREEGAGEVEFQGAT
EEDQDMANCLMLLSQGHKANGSGDHSSTHKIDFLCKKKPVASLGLGLEGVYQCQTCDKSF
HSFQALGGHRASHKKPKLGENVLKCHEKKSSSAFAVETAKGSFLSLQVTSSDGNKKPEKT
HECSICKAEFSSGQALGGHMRRHRGLIVNANATSSHHQESIRPKNFLELDLNLPAPEDES
KFVFASKDQIILFTTASNSLIDCHH
> BnaAZFP14(MyBnaA04g16320D)
MMSQDHVGSDQTQIIKGKRTKRQRSLSSTFLVAAAAATTNTSSSSSAGDGGGGRAVSDEY
NSAVSSPVTTTDCTEEEEDMAICLIMLARGAALSPDLKNSRKADKTFSPAENSSFFVYEC
KTCSRTFSSFQALGGHRASHKKPRVSIEEKTKLPLMQAKSSGSEEGQKNNFKVFGSSLAL
LSSNIIISKANKVHECSICGSEFTSGQALGGHMRRHRTATTAVIPVATTEVSRNSTEEET
ENLSSYIEQRKYLPLDLNLPAPEDDLRESKFQGIVFSTTPALIDCHY
> BnaAZFP19(MyBnaA05g28380D)
METAEEAISAAKEQALILKGKRTKRQRLQSPIPFSIVPPMSSQEPDVEEESTSLVSKEKS
LNDDINTNKNDNNVLINGVTSPASSSSNNNATLKTAADEEDQDMANCLILLAQGHYTPQQ
QPQQTRQFMMSYQESGNNNNNAYRSSSRRFLETSSPNGTTSGGRAGYYVYQCKTCDRTFP
SFQALGGHRASHKKPKAAAGLHSDHDLKKSIYNDAVSLHLNNVPTATPNNNSSHRSLVVY
GKAGNNKVHECGICGAEFTSGQALGGHMRRHRGAVVASAASASTATVRVAATAGTANTAL
SLSPMSFDHMSVHPVQGPVKRARSAVVSLDLDLNLPAPEDVNRVNGLSFASKQEQEHEHE
QTQQREEQKSIVLSSAPTLVDCHY
> BnaAZFP26(MyBnaA07g13700D)
MMGQDHEVGSDQTQIIKGKRTKRQRSSSSTFLVAAAAPTITSTSSSAGGERTASEEYNSV
VSSPVTTTDCTEEEEDMAICLIMLARGAAPSPPLPDLKNSTKTDKNLYQKNSSFYVYECK
TCNRTFPSFQALGGHRASHKKPRASIDEKAKVPLTQLKSSASEEGQKGHFKVSGPALASK
ASNIIISKANKVHECSICGSEFTSGQALGGHMRRHRTVTNVVSSPVTAAEVSRNSTDEET
ENLSRSMEQRKYLPLDLNLPAPEDDLRESKFQGIVFSATTPALIDCHY
> BnaAZFP29(MyBnaA09g07340D)

MALETLNSPTSATATARPFLRYRKEMEPENLEQWAKRKRTKRQRFDHNQEKTPSEEEYLA
LCLLMLARGSTVQSLPPPSLPSSDHRGYKCTVCGKSFSSYQALGGHKTSHRKPVNNTDVP
SNQEPFNNTHRNSNGGSVVINGNGVSQSGKTHTCSICFKSFSSGQALGGHKRCHYDGGNN
GNGSSSNSVEVIGGSDVSDVDDERSSEQSGIGGHRGFDLNLPADQVTVVIS
> BnaAZFP31(MyBnaA10g00100D)
MNLSCIDFVMFSSRGQHDEGNMSRPSWKRERSNNLINLSPNEDEELANCLVLLSNSGD
HYNSGGHHNKHGHGKGKTIKKQKTSQAFQCKACKKVFTSHQALGGHRASHKKVKGCFATQ
NKEEEEDEDEYKEEDEEEEEEEEEDKATAADYNNIITRKRSNAHECTICHRVFSSGQALG
GHKRCHWLTPSSYFHMTPLHDSSSVARSQMLEQPSLDLNLTCQEYSVDPTVMSVWRDDGG
NNHNATSPDSWLKLASGDWS
> BnaAZFP33(MyBnaA10g25770D)
MEAFVEAIAASKEQSLIFKGKRTKRQRPQSPIPFSIAPPIVSSHARDILEESKKDGVITS
SSSSASWFSNNNATLKAEEDEEEQDIANCLILLSQGHSLPIPNHEANNNNTFRFSSRRFL
ETSSSNGGGKAGYYVYQCKTCDRTFPSFQALGGHRASHKKPKATLSLYSNIDVKKNIYES
DAVSLVTTSTIYKNNNNRSLAVYGKAGSNKVHECGVCGAEFTSGQALGGHMRRHRGAVVI
AAAPVTTVTVATAAANTELSLSSMSFDQISDGQDHLVMPATKRAKKTVVSLDLDLNLPAS
EDENRVNGFTFALKQKQEQEHQPTMQREEPKCLLMSAPTLVDCRY
> BnaAZFP37(MyBnaAnng17660D)
MEAFEEAIAASKEQSLILKGKRTKRQRPQSPVPFSSSPPIVSCHAHDIEEEYTDLDSKEN
ALGNNVENHNKDGVITSSSSSASWSSNNNPTLKAEEDEEDLDIASCLILLSRGHSLPQLK
IPNHETTYNNKTYKFCSRRFLETSSSNGGGKAGYYVYQCKSCDRTFSSFQALGGHRASHK
KPKATSFYSNLDHLKKNIYENDSLSTTTTIYNNNKNRSLVVYGKAGNNKVHECGICGAEF
TSGQALGGHMRRHRGAVVVAPTVTVALAAANTELSLSSMSFDQISTKRAKKMVVSLDLDL
NLPAPEDENRVNGFSLGFKQKHEQEHQQTKQRDEPKCLVLSPPTLVDCHY
> BnaAZFP38(MyBnaAnng05120D)
MALETLNSPTSATASARPLLRYREEMEPENLEQWAKRKRTKRQRFDQSRLNQETAPSEEE
YLALCLLMLARGSAVQSPLPPSSSSDHRGYKCTVCGKSFSSYQALGGHKTSHRKPASNVN
VPINQEQSNNSHSNSNGGSVAINGNGVSQSGKIHTCSICFKSFSSGQALGGHKRCHYDAG
NNGNGNGSSSNSVEVVGGSDGSYVDDERSSEQSATGDNRGFDLNLPADQVAVVIS
