# Supplementary material for: Comparative analysis of the genetic variability within the Q-type C2H2 zinc-finger transcription factors in the economically important cabbage, canola and Chinese cabbage genomes
Source: Hereditas. 2018 Sep 21;155:29. doi: 10.1186/s41065-018-0065-5 (PMC6150991; doi:10.1186/s41065-018-0065-5)
Supplement: Supplementary file 2 — Alignment of Q-type-ZFP proteins from Brassica oleracea, B. rapa and B. napus by group. (PDF 79 kb) [file 41065_2018_65_MOESM2_ESM.pdf]

Additional File 2: Alignment of Q-type-ZFP proteins from *Brassica oleracea*, *B. rapa* and *B. napus* by group
Title; Comparative analysis of the genetic variability within the Q-type C2H2 zinc-finger transcription factors in the economically important cabbage, canola and Chinese cabbage genomes

Additional File 2 *Brassica oleracea, rapa and napus* ZFP proteins were aligned by subgroups.

Alignment of 2i-A proteins p 2-3
Alignment of 2i-B proteins p 4-5
Alignment of 2i-C proteins p 6-12
Alignment of 2i-D proteins p13-18
Alignment of 2i-X proteins p19

```
2i-A Brassica ZFPs         ----L-box------                       -ZF#1----
BnaAZFP16      MMKRERSEFEESIKNL-DISKCLMILSQTSSMVKQIGV--NQYTETNTSNRFECKTCNKR 57
BrZFP17        MMKRERSEFEESIKNL-DISKCLMILSQTSSMVKQIGV--NQYTETNTSNRFECKTCNKR 57
BoZFP22        -MKRERSEFEESIKNL-DIAKCLMILAQTSSMVKQIGV--NQYAESNSSNRFECKTCNKR 56
BnaCZFP19      -MKRERSEFEESIKNL-DIAKCLMILSQTSSMVKQIGV--NQYAESNSSNRFECKTCNKR 56
BnaAZFP17      -MKRERSEFEESIKMSDDIARCLMILSQTS-MVKQVDV--NQYTERDTSNRFECKTCNKR 56
BrZFP19        -MKRERSEFEESIKMSDDIARCLMILSQTS-MVKQVDV--NQYTERDTSNRFECKTCNKR 56
BoZFP17        -MKRERSEFEESIKMSDDIARCLMILSQTS-MVKQVDV--NQYTDRDTSNRFECKTCNKR 56
BnaCZFP14      -MKRERSEFEESIKMSDDIARCLMILSQTS-MVKQVDV--NQYTDRDTSNRFECKTCNKR 56
BnaAZFP9       -MKRERSEYEESIKNL-DIVKCLMILSRTS-IAKQIGVIHNQFTERNTSNRFECKTCNRR 57
BnaCZFP36      -MKRERSEYEESIKNL-DIVKCLMILSRTS-IVKQIGV--NQFTERNTSNRFECKTCNRR 55
BnaAZFP25      -MKRDRSDYEESITDL-DIVKSLMMLSQSF-VVKQIEVQQ-STRSRTNDNQFECKTCNRK 56
BrZFP27        -MKRDRSDYEESITDL-DIVKSLMMLSQSF-VVKQIEVQQ-STRSRTNDNQFECKTCNRK 56
BoZFP28        -MKRDRSDYEESITDL-DIVKSLMMLSQSF-VVKQIEVKQ-STRTRTNDNQFECKTCNRK 56
BnaCZFP25      -MKRDRSDYEESITDL-DIVKSLMMLSQSF-VVKQIEVKQ-STRTRTNDNQFECKTCNRK 56
BnaAZFP13      -MKRDRSDYEDFITHI-DMIKTLTMSSHSF-VVKQIES----TGSKINGNRFECKTCNRK 53
BrZFP14        -MKRDRSDYEDFITHI-DMIKTLTMSSHSF-VVKQIES----TGSKINGNRFECKTCNRK 53
BoZFP1         -MKRDRSDYEDFITHI-DMVKTITMLSHSC-VVKQIES----TGSKTKGNRFECKTCNRK 53
BnaCZFP17      -MKRDRSDYEDFITHI-DMVKTITMLSHSC-VVKQIES----TGSKTKGNRFECKTCNRK 53
                ***:**::*: *.   *: : : : :::  :.**:           . *:*******::
               -----------NLS-------                 -------ZF#2----------
BnaAZFP16      FSSFQALGGHRASHKKPKL-TVDQKVVKQYL-TKDGTQAHECTICGQSFGTGQALGGHMR 115
BrZFP17        FSSFQALGGHRASHKKPKL-TVDQKVVKQYL-TKDGTQAHECTICGQSFGTGQALGGHMR 115
BoZFP22        FSSFQALGGHRASHKKPKL-TVDQKVVKQYL-TKEGTQAHECTICGQSFGTGQALGGHMR 114
BnaCZFP19      FSSFQALGGHRASHKKPKL-TVDQKVVKQYL-TKEGTQAHECTICGKSFGTGQALGGHMR 114
BnaAZFP17      FSSFQALGGHRASHKKPKL-SVDQKEVKHVTNNYNGTHIHECSICSQSFGTGQALGGHMR 115
BrZFP19        FSSFQALGGHRASHKKPKL-SVDQKEVKHVTNNYNGTHIHECSICSQSFGTGQALGGHMR 115
BoZFP17        FSSFQALGGHRASHKKPKL-SVDQKEVKHVTNNYNGTHMHECSICGQSFGTGQALGGHMR 115
BnaCZFP14      FSSFQALGGHRASHKKPKL-SVDQKEVKHVTNNYNGTHMHECSICGQSFGTGQALGGHMR 115
BnaAZFP9       FSSFQALGGHRASHKKPKL-AVDEKDVKHLTKNYKGTHMHKCSRCDQSFGTGQALGGHMR 116
BnaCZFP36      FSSFQALGGHRATHKKPKL-TVDEKDVKHFTKNYKGTHMHKCSRCGQSFGTGQALGGHMR 114
BnaAZFP25      FDSFQALGGHRASHKKPKL-SIDQEQVKH---RYNENAAHKCSICGQMFGTGQALGGHMR 112
BrZFP27        FDSFQALGGHRASHKKPKL-SIDQEQVKH---RYNENAAHKCSICGQMFGTGQALGGHMR 112
BoZFP28        FDSFQALGGHRASHKKPKL-SIDQEQVKH---RNNENAAHKCSTCGQMFGTGQALGGHMR 112
BnaCZFP25      FDSFQALGGHRASHKKPKL-SIDQEQVKH---RNNENAAHKCSTCGQMFGTGQALGGHMR 112
BnaAZFP13      FDSFQALGGHRASHKKPKLMSVEQEQAKH---RNNENDVHKCSICSQMFGTGQALGGHIR 110
BrZFP14        FDSFQALGGHRASHKKPKLMSVEQEQAKH---RNNENDVHKCSICSQMFGTGQALGGHIR 110
BoZFP1         FDSFQALGGHRASHKKPKLVSVEQEQAKH--------TVHKCSICGQMFGTGQALGGHMR 105
BnaCZFP17      FDSFQALGGHRASHKKPKLVSVEQEQAKH--------TVHKCSICGQMFGTGQALGGHMR 105
               *.*********:******  ::::: .*:           *:*: * : **********:*
               ---                                --EAR----
BnaAZFP16      RHRSSMTVEPSELISPVIHNMPVLKRCSSSKRVLSLDLNLTPLENDLEILFGKTFFPNID 175
BrZFP17        RHRSSMTVEPSELISPVIHNMPVLKRCSSSKRVLSLDLNLTPLENDLEILFGKTFFPNID 175
BoZFP22        RHRSSMMVEPSELISPVIHDMPVLKRCSSSKRVLSLDLNLTPLENDLEILFGKTFFPNID 174
BnaCZFP19      RHRSSMMVEPSELISPVIHNMPVLKRCSSSKRVLSLDLNLTPLENDLEILFGKTFFPNID 174
BnaAZFP17      RHRSSVTVEPLQTISPVNSTVPVLKRCSSSKRVLSLDLNLTPLENDLETIFGKTFFPNID 175
BrZFP19        RHRSSVTVEPLQTISPVNSTVPVLKRCSSSKRVLSLDLNLTPLENDLETIFGKTFFPNID 175
BoZFP17        RHRSSMKVEPSQLISPVISNVPVLKRCSSSKRVLSLDLNLTPLENDLETIFGKTFFPNID 175
BnaCZFP14      RHRSSMKVEPSQLISPVISNVPVLKRCSSSKRVLSLDLNLTPLENDLETIFGKTFFPNID 175
BnaAZFP9       RHRSSMTLEPSQRISPRIPTMPVMKRCSSSKRVLSLDLNLTPLENDLEIIFGKKFFPNID 176
BnaCZFP36      RHRSSMTLEPSQRISPVIPTMPVMKRCSSSKRVLSLDLNLTPLENDLEIIFGKKFFPNID 174
BnaAZFP25      KHRESMRTEQPVVSSTVY---PVLNRCNTSK---CLDLNLTPLENDLVYVFGKNLAD--- 163
BrZFP27        KHRESMRTEQPVVSSTVY---PVLNRCNTSK---CLDLNLTPLENDLVYVFGKNLAD--- 163
BoZFP28        KHRESMRTEQPVVSSTVY---PVLNRCNSSK---CLDLNLTPLENDLVYAFGKNLAD--- 163
BnaCZFP25      KHRESMRTEQPVDSSTVY---PVLNRCNSSK---CLDLNLTPLENDLVYAFGKNLAD--- 163
BnaAZFP13      RHRASMINEQSVISSKVYT-SPVNDQCQSSKRVMRLDLNLTPLENDLLNIFAKNLAPHID 169
BrZFP14        RHRASMINEQSVISSKVYT-SPVNDQCQSSKRVMRLDLNLTPLENDLLNIFAKNLAPHID 169
BoZFP1         KHRESMINEQSVISSMVYSTSPVMNRCNSSKRVMRLDLNLTPLENDLVYIFAKNLVPHID 165
BnaCZFP17      KHRESMINEQSVISSMVCSTSPVMN---SSKRVMRLDLNLTPLENDLVYIFAKNLVPHID 162
               :** *:  *     *      ** .   :**    ************   *.*.:
```

```
BnaAZFP16      MKFVV         180
BrZFP17        MKFVV         180
BoZFP22        MKFVV         179
BnaCZFP19      MKFVV         179
BnaAZFP17      MKFVV         180
BrZFP19        MKFVV         180
BoZFP17        MKFVV         180
BnaCZFP14      MKFAV         180
BnaAZFP9       MKFVV         181
BnaCZFP36      MKLVV         179
BnaAZFP25      FKFVN         168
BrZFP27        FKFVN         168
BoZFP28        LKFVN         168
BnaCZFP25      LKFVN         168
BnaAZFP13      LKFVN         174
BrZFP14        LKFVN         174
BoZFP1         LKFVN         170
BnaCZFP17      LKFVN         167
               :*:.
```

```
2i-B Brassica ZFP                                      -----L-box------
BoZFP21         -------------------------------MEKEREIEMINKMASCLIYLSKAHQHD--27
BnaCZFP18       -------------------------------MEKEREIEMINKMASCLIYLSKAHQHD--27
BrZFP16         -------------------------------MEKEREIEMINKMASCLIYLSKAHQHD--27
BnaAZFP15       -------------------------------MEKEREIEMINKMASCLIYLSKAHQHD--27
BoZFP18         -------------------------------MEKEREMEMINKMASCLIFLSKAHQHD--27
BnaCZFP15       -------------------------------MEKEREMEMINKMASCLIFLSKAHQHD--27
BrZFP26         -------------------------------MEKEREMEMINKMASCLIFLSKAHQHD--27
BnaAZFP27       -------------------------------MEKEREMEIINKMASCLIFLSKAHQHD--27
BoZFP34         ----------------------------MVAISEIKLTVETTAAANCLMLLSRVGQENVD32
BnaC0ZFP30      ----------------------------MVAISEIKLTVETTAAANCLMLLSRVGQENVD32
BrZFP33         ----------------------------MVAISEIKSTVETT-AANCLMLLSRVGQENVD31
BnaAZFP32       ----------------------------MVAISEIKSTVETT-AANCLMLLSRVGQENVD31
BrZFP10         ----------------------------MVAISEINPTVEAK-AANCLMLLSRVGQKGG-30
BrZFP11         ----------------------------MVAISEINPTVEAK-AANCLMLLSRVGQKGG-30
BnaAZFP8        ----------------------------MVAISEINPTVEAK-AANCLMLLSRVGQKGG-30
BoZFP12         ----------------------------MVAISEINPTVEAT-AANCLMLLSRVGQNGA-30
BnaCZFP10       ----------------------------MVAISEINPTVEAT-AANCLMLLSRVGQKGA-30
BnaAZFP5        ----------------------------MVAVSENKSTVEAT-AANCLMLLSRVGQENG-30
BoZFP8          ------------------------------MVAEIKSTVEAT-AANCLMLLSRVGQENG-28
BrZFP6          ------------------------------MVAEIKSTVEAT-AANCLMLLSRVRQENG-28
BnaAZFP4        ------------------------------MVAEIKSTVEAT-AANCLMLLSRVRQENG-28
BnaAZFP21       ----------------------------MVSRSESFDRTVEDTTTNCLMLLSRVGECGG-31
BoZFP15         MVSRSENLNRTVENRAQDSEKLDRTVEGTAANNEKLERTVEDKAANCLMLLSRIGENGG-59
BnaCZFP12       MVARSENLNRTVENRAQDSEKLDRTVEGTAANNEKLERTVEDKAANCLMLLSRIGENGG-59
BrZFP21         --------------MVARSENLDRTVEGTAGNNEKLERTVEDKAANCLMLLSRIGENGG-45
BnaAZFP20       --------------MVARSEKLDRTVEGTAGNNEKLERTVEDKAANCLMLLSRIGENGG-45
BoZFP2          --------------MVA--RSENRSVKDTPAENENFDRTVEDTATSCLMLLPRVGKCI--42
BnaCZFP1        --------------MVA--RSENRSVKDTPAENENFDRTVEDTATSCLMLLPRVGKCI--42
BrZFP22         ----------------------------MVARSENLDQTVEDTEATCLMLLSRVGECGR-31
BnaAZFP22       ----------------------------MVVRSENLDQTVEDTEATCLMLLSKVGECGR-31
                                                 :          :.**: * :  :
                     ------ZF#1----------------
BoZFP21         --TKSRVFACKTCNKEFPSFQALGGHRASHRRSAALEGHATP------------SPKRVK73
BnaCZFP18       --TKSRVFACKTCNKEFPSFQALGGHRASHRRSAALEGHATP------------SPKRVK73
BrZFP16         --TKSRVFACKTCNKEFPSFQALGGHRASHRRSAALEGHAPP------------SPKRVK73
BnaAZFP15       --TKSRVFACKTCNKEFPSFQALGGHRASHRRSAALEGHAPP------------SPKRVK73
BoZFP18         --TKGRVFACKTCNKEFSSFQALGGHRASHRRSAALEGHAPP------------SPKRVK73
BnaCZFP15       --TKGRVFACKTCNKEFSSFQALGGHRASHRRSAALEGHAPP------------SPKRVK73
BrZFP26         --TKGRVFACKTCNKEFSSFQALGGHRASHRRSAALEGHAPP------------SPKRVK73
BnaAZFP27       --TKGRVFACKTCNKEFSSFQALGGHRASHRRSAALEGHAPP------------SPKRVK73
BoZFP34         GGSAKRVFTCKTCLKEFHSFQALGGHRASHKKPNN-ESLS-------GL-----IKKSKA79
BnaC0ZFP30      GGSAKRVFTCKTCLKEFHSFQALGGHRASHKKPNN-ESLS-------GL-----IKKSKA79
BrZFP33         GGSAKRVFTCKTCLKEFLSFQAWGGHRASHKKPNN-ENLS-------SL-----MKKTKA78
BnaAZFP32       GGSAKRVFTCKTCLKEFHSFQALGGHRASHKKPNN-ENLS-------SL-----MKKTKA78
BrZFP10         D--QKRVFTCKTCLKEFHSFQALGGHRASHKKPNNNESLS-------GL-----VKKAKA76
BrZFP11         D--QKRVFTCKTCLKEFHSFQALGGHRASHKKPNNNESLS-------GL-----VKKAKA76
BnaAZFP8        D--QKRVFTCKTCLKEFHSFQALGGHRASHKKPNNNESLS-------GL-----VKKAKA76
BoZFP12         D--QKRVFTCKTCLKEFHSFQALGGHRASHKKPN-NESLS-------GL-----VKKAKA75
BnaCZFP10       D--QKRVFTCKTCLKEFHSFQALGGHRASHKKPN-NESLS-------GL-----VKKAKA75
BnaAZFP5        GDYKKRVFTCKTCLKEFQSFQALGGHRASHNKPNN-ENLS-------GL-----IKKAKT77
BoZFP8          GDA-KRVFTCKTCLKEFHSFQALGGHRASHRKPNN-ENLS-------GL-----IKKAKT74
BrZFP6          GDT-KRVFTCKTCLKEFHSFQALGGHRASHKKPNN-ENLS-------GL-----IKKAKT74
BnaAZFP4        GDT-KRVFTCKTCLKEFHSFQALGGHRASHKKPNN-ENLS-------GL-----IKKAKT74
BnaAZFP21       GDVEGRVFRCKTCMKEFSSFHALGVHRVSHKKPVSNSNGEEQSGIFIK------KKTKKT85
BoZFP15         GGRESRVYRCKTCMKEFSSFQSLGGHRASHNKHVNNNSSDEQSSLSSGSIAK--RKTTNT117
BnaCZFP12       GGRESRVYRCKTCMKEFSSFQSLGGHRASHNKHVNNNSSDEQSSLSSGSIAK--RKTTNT117
BrZFP21         GGRESRVYRCKTCMKEFSSFQSLGGHRASHNKHVDNSSSDEQSSLFSGSIAKTKRKTTNT105
BnaAZFP20       GGRESRVYRCKTCMKEFSSFQSLGGHRASHNKHVNNSSSDEQSSLSSGSIAKPKRKTTNT105
BoZFP2          -RGESRIYRCKTCMKEFSSFQALGGHRASHKKPINNSSDDCS---SSGSVTKNT----KK94
BnaCZFP1        -RGESRIYRCKTCMKEFSSFQALGGHRASHKKPISNSSDDCS---SSGSVTKNT----KK94
BrZFP22         ERGETRVFRCKTCMKEFSSFQALGGHRASHKKPVNNSSDEQSSSLSSGSVTKKT----KK87
BnaAZFP22       ERGETRVFRCKTCTKEFSSFQALGGHRASHKKPVNNSSDEQSSSLSSGSVTKKT----KK87
                     *:: **** *** **:: * **.**.:    .
```

```
                   --------ZF#2-----------
BoZFP21         PVKHECPICGAEFAVGQALGGHMRKHRGGGG------SRSLAPAPV---TMKKSGGGNGK 124
BnaCZFP18       PVKHECPICGAEFAVGQALGGHMRKHRGGGG------SRSLAPAPV---TMKKSGGGNGK 124
BrZFP16         PVKHECPICGAEFAVGQALGGHMRKHRGGGG------SRSLAPAPV---TMKKSGGGNGK 124
BnaAZFP15       PVKHECPICGAEFAVGQALGGHMRKHRGGGG------SRSLAPAPV---TMKKSGGGNGK 124
BoZFP18         PVKHECPICGAEFAVGQALGGHMRKHRGGGA------SRSLAPAPV---TMKKTGGGNAK 124
BnaCZFP15       PVKHECPICGAEFAVGQALGGHMRKHRGGGA------SRSLAPAPV---TMKKTGGGNAK 124
BrZFP26         PVKHECPICGAEFAVGQALGGHMRKHRGGGS------SRSLAPAPV---TMKKTGGGNAK 124
BnaAZFP27       PVKHECPICGAEFAVGQALGGHMRKHRGGGS------SRSLAPAPV---TMKKTGGGNAK 124
BoZFP34         ASSHPCPICGVEFPMGQALGGHMRRHRNESGGAGALVTRELLSEAA---LTTLKKSSSG- 135
BnaC0ZFP30      ASSHPCPICGVEFPMGQALGGHMRRHRNESGGAGALVTRELLSEAA---LTTLKKSSSG- 135
BrZFP33         SSSHPCPICGVEFPMGQALGGHMRRHRNESGGAGALVTRELLSEAA---LTTLKKSSSG- 134
BnaAZFP32       SSSHPCPICGVEFPMGQALGGHMRRHRNESGGAGALVTRELLSEAA---LTTLKKSSSG- 134
BrZFP10         PSSHPCPICGVEFPMGQALGGHMRRHRNEIGGGAALVTRALLPEPT---MTTLKKSSSGK 133
BrZFP11         PSSHPCPICGVEFPMGQALGGHMRRHRNEIGGGAALVTRALLPEPT---MTTLKKSSSGK 133
BnaAZFP8        PSSHPCPICGVEFPMGQALGGHMRRHRNEIGGGAALVTRALLPEPT---MTTLKKSSSGK 133
BoZFP12         PSSHPCPICGVEFPMGQALGGHMRRHRNESGGGAALVTRALLPEPT---MTTLKKSSSGK 132
BnaCZFP10       PSSHPCPICGVEFPMGQALGGHMRRHRNESGGGAALVTRALLPEPT---MTTLKKSSSGK 132
BnaAZFP5        PSSHPCPICGVEFPMGQALGGHMRKHRNENGGGVTLVTRALLPEPT---MTTLKKSSSGK 134
BoZFP8          PSSHPCPICGVEFPMGQALGGHMRKHRNENGGGVTLVTRALLPEPT---VTTLKKSSSGK 131
BrZFP6          PSSHPCPICGVEFPMGQALGGHMRKHRNENGGGVALVTRALLPEPT---VTTLKKTSSGK 131
BnaAZFP4        PSSHPCPICGVEFPMGQALGGHMRKHRNENGGGVALVTRALLPEPT---VTTLKKTSSGK 131
BnaAZFP21       GWHHRCSICGLEFEMGQALGGHMRKHWNKEDSGGALITRSFFPEAA--MTRTLKETSKEK 143
BoZFP15         TRSHRCPICGVEFPMGQALGGHMRKHRNEEEASGALVTRSFFPEAAASMITTLKKSSSGK 177
BnaCZFP12       TRSHRCPICGVEFPMGQALGGHMRKHRNEEEASGALVTRSFFPEAATSMITTLKKSSSGK 177
BrZFP21         TRSHRCPICGVEFPIGQALGGHMRKHRNEEEASGALVTRSFFPEAATSMITTLKKSSSGK 165
BnaAZFP20       TRSHPCPICGVEFPIGQALGGHMRKHRNEEEASGALVARPFFPEAATSMITTLKKSSSGK 165
BoZFP2          QSSHTCPICGLEFPMGQALGGHMRKHRNDKE-SLALVTRSFLPEVL---TTTLKKSSSGK 150
BnaCZFP1        QSSHTCPICGLEFPMGQALGGHMRKHRNDKE-SLALVTRSFLPEVL---TTTLKKSSSGK 150
BrZFP22         PLSHTCPICGLEFPMGQALGGHMRKHRNEKE-RGALVTRSFLPEAK---FTTLKKSSSGK 143
BnaAZFP22       PLSHTCPICGLEFPMGQALGGHMRKHRNEKE-RGALVTRSFLPEAK---FTTLKKSSSGK 143
                   * * *** ** :*********:*           :* :          . .  ..
                                 -EAR--
BoZFP21         RVLCMDLNLTPGEN-DDLKLELGRL--     148
BnaCZFP18       RVLCMDLNLTPGEN-DDLKLELGRL--     148
BrZFP16         RVLCMDLNLTPGEN-DDLKLELGML--     148
BnaAZFP15       RVLCMDLNLTPGEN-DDLKLELGML--     148
BoZFP18         RVLCLDLNLTPVEN-EDLKLELGRLIF     150
BnaCZFP15       RVLCLDLNLTPVEN-EDLKLELGRLIF     150
BrZFP26         RVLCLDLNLTPVEN-EDLKLELGRLIL     150
BnaAZFP27       RVLCLDLNLTPVEN-EDLKLELGRLIL     150
BoZFP34         RLACLDLSLGMVEN-LNLKLELGRPVC     161
BnaC0ZFP30      RLACLDLSLGMVEN-LNLKLELGRPVC     161
BrZFP33         RLACLDLSLGMVEN-LNLKLELGRPVC     160
BnaAZFP32       RLACLDLSLGMVEN-LNLKLELGRPVC     160
BrZFP10         RVACLDLSLGMVEN-LNLKLELGRTVC     159
BrZFP11         RVACLDLSLGMVEN-LNLKLELGRTVC     159
BnaAZFP8        RVACLDLSLGMVEN-LNLKLELGRTVC     159
BoZFP12         RVACLDLSLGMVEN-LNLKLELGRTVC     158
BnaCZFP10       RVACLDLSLGMVEN-LNLKLELGRTVC     158
BnaAZFP5        RVACLDLSLGMVEN-LNLKLELGRTVY     160
BoZFP8          RAACLDLSLGMVEN-LNLKLELGRTVY     157
BrZFP6          RVACLDLSLGMVEN-LNLKLELGRTVY     157
BnaAZFP4        RVACLDLSLGMVEN-LNLKLELGRTVY     157
BnaAZFP21       GVACLEPGSDSIEG-INLKLELGRKMY     169
BoZFP15         RVECFDLGPDSVESSINLNLELGRSMY     204
BnaCZFP12       RVECFDLGPDSAESSINLNLELGRSMY     204
BrZFP21         RVACFDLGPDSVESSINLNLELGSSMY     192
BnaAZFP20       RVACFDLGPDSVESSINLNLELGSSMY     192
BoZFP2          RVACFDSELDSMEGIINLNLELGISIY     177
BnaCZFP1        RVACFDSELDSMEGIINLNLELGISIY     177
BrZFP22         RVACFGMGLDPMERVINLKLELGRTIY     170
BnaAZFP22       RVACFGMGLDPMERVINLKLELGRTIY     170
                   *:       *   :*:****
```

```
2i-C Brassica proteins            ------NLS------
BoZFP27        METAEEAISAAKEQALILKGKRTKRQRLQSPIPFSIVPPM--SSQEPDVEDESTSLVSKE 58
BnaCZFP24      METAEEAISAAKEQALILKGKRTKRQRLQSPIPFSIVPPM--SSQEPDVEDESTSLVSKE 58
BrZFP18        METAEEAISAAKEQALILKGKRTKRQRLQSPIPFSIVPPM--SSQEPDVEEESTSLVSKE 58
BnaAZFP19      METAEEAISAAKEQALILKGKRTKRQRLQSPIPFSIVPPM--SSQEPDVEEESTSLVSKE 58
BoZFP4         METAEEAISAAKAQALIIKGKRTKRQRPQSPISFSIVAPM--SSQEPDAQEESTSLVAKE 58
BrZFP3         METAEEAISAAKAQALIIKGKRTKRQRPQSPIPFSIVPPM--SSQEPDTQEESTSLVAKE 58
BnaAZFP3       METAEEAISAAKAQALIIKGKRTKRQRPQSPIPFSIVPPM--SSQEPDTQEESTSLVAKE 58
BrZFP9         MEAFEEAIAASKEQALILKGKRTKRQRPQSPIPFSV-SPPIVEEEVSNVL------DSK- 52
BnaAZFP7       MEAFEEAIAASKEQALILKGKRTKRQRPQSPIPFSV-SPPIVEEEVSNVL------DSK- 52
BoZFP11        MEAFEEAIAASKEQALILRGKRTKRQRPQSPIPFSV-SPPIVEEEVSNVL------DSK- 52
BnaCZFP7       MEAFEEAIAASKEQALILKGKRTKRQRPQSPIPFSV-SPPIVEEEVSNVL------DSK- 52
BrZFP34        MEAFVEAIAASKEQSLIFKGKRTKRQRPQSPIPFSI-APPIVSSHARDILE--------- 50
BnaAZFP33      MEAFVEAIAASKEQSLIFKGKRTKRQRPQSPIPFSI-APPIVSSHARDILE--------- 50
BoZFP35        MEAFEEAIAASKEQSLIFKGKRTKRQRPQSPIPFSIISPPIVSSHAHDIQE--------- 51
BnaCZFP31      MEAFEEAIAASKEQSLIFKGKRTKRQRPQSPIPFSIISPPIVSSHAHDIQE--------- 51
BoZFP7         MEAFEEAIAASKEQSLILKGKREKRQRSQSPVPFSI-SPPIVSCPSRDVEEEYTNLDSKE 59
BrZFP5         MEAFEEAIAASKEQSLILKGKRTKRQRPQSPVPFSS-SPPIVSCHAHDIEEEYTDLDSKE 59
BnaAZFP37      MEAFEEAIAASKEQSLILKGKRTKRQRPQSPVPFSS-SPPIVSCHAHDIEEEYTDLDSKE 59
BrZFP4         ---MT-SFHEETRLVLLTKGKRTKRPRSSSPHMNAEAVSGVC--------SEDRSLEAK- 47
BnaAZFP36      ---MT-SFHEETRLVLLTKGKRTKRPRSSSPHMNAEAVSGVC--------SEDRSLEAK- 47
BoZFP6         ---MT-SFHEETRLVLLTKGKRTKRPRSESPHMNAEAVSGVC--------SDRSLGAKE- 47
BnaCZFP5       ---MT-SFHEETRLVLLTKGKRTKRPRSESPHMNAEAVSGVC--------SDRSLGAKE- 47
BoZFP37        ---MT-SVHEETRLVLLVKGKRTKRQRSASPHMNAEAVSGIC--------SEEPSLEARE 48
BnaAZFP12      ---MT-SVHEETRLVLLVKGKRTKRQRSASPHMNAEAVSGVC--------SEEPSLEARE 48
BnaCZFP35      ---MT-SVHEETRLVLLVKGKRTKRQRSASPHMNAEAVSGVC--------SEEPSLEARE 48
BrZFP35        ---MT-SVHEEMRHVLLIKGKRTKRQRSASPHMNAEAVSGVC--------SEEPSLEARE 48
BnaAZFP35      ---MT-SVHEEMRHVLLIKGKRTKRQRSASPHMNAEAVSGVC--------SEERSLEARE 48
BoZFP10        ---MT-SVHEETRLVLLIKGKRTKRQRSASPHIKAEVMSSVC--------NEERSLEARE 48
BnaCZFP8       ---MT-SVHEETRLVLLIKGKRTKRQRSASPHIKAEVMSSVC--------NEERSLEARE 48
BrZFP8         ---MT-SVHEETRLVLLIKGKRTKRQRSVSPHMNAEAMSSVC--------KEERSLEAR- 47
BnaAZFP6       ---MT-SVHEETRLVLLIKGKRTKRQRSVSPHMNAEAMSSVC--------KEERSLEAR- 47
BrZFP15        --MM-SQDHVGSDQTQIIKGKRTKRQRSSSSTFLVAAAAATT--------NTSSSSSAGD 49
BoZFP20        --MM-SQDHVGSDQTQIIKGKRTKRQRSSSSTFLVAAAAATT--------NTSSNSSAGD 49
BnaCZFP32      --MM-SQDHVGSDQTQIIKGKRTKRQRSSSSTFLVAAAAATT--------NTSSNSSAGD 49
BnaAZFP14      --MM-SQDHVGSDQTQIIKGKRTKRQRSLSSTFLVAAAAATT--------NTSSSSSAGD 49
BoZFP19        --MMGQDHEVGSDQTQIIKGKRTKRQRSSSSTFLVAAAATTI--------TST----SSS 46
BnaCZFP16      --MMGQDHEVGSDQTQIIKGKRTKRQRSSSSTFLVAAAATTI--------TST----SSS 46
BrZFP25        --MMGQDHEVGSDQTQIIKGKRTKRQRSSSSTFLVAAAAPTI--------TST----SSS 46
BnaAZFP26      --MMGQDHEVGSDQTQIIKGKRTKRQRSSSSTFLVAAAAPTI--------TST----SSS 46
                               : :*** ** *  *
```

```
                                                                ------L-box-----
BoZFP27         KSLNDEINT--NKNDNNMLSNGVTSPASS-SSNNNATLKTAADEEDQDMANCLILLAQGH  115
BnaCZFP24       KSLNDEINT--NKNDNNMLSNGVTSPASS-SSNNNATLKTAADEEDQDMANCLILLAQGH  115
BrZFP18         KSLNDDINT--NKNDNNVLINGVTSPASS-SSNNNATLKTAADEEDQDMANCLILLAQGH  115
BnaAZFP19       KSLNDDINT--NKNDNNVLINGVTSPASS-SSNNNATLKTAADEEDQDMANCLILLAQGH  115
BoZFP4          KSLNDEINYNNNKNDNNILSNGVTSSSSTSSCFNNATLKAAADEEDQDMANCLILLAKGH  118
BrZFP3          KSLNDEINYNNNKNDNNILSIGVTSSSSTSSSFNNATLKAAADEEDQDMANCLILLAQGH  118
BnaAZFP3        KSLNDEINYNNNKNDNNILSIGVTSSSSTSSSFNNATLKAAADEEDQDMANCLILLAQGH  118
BrZFP9          ---ENDVAN--RKKDGVITS---SSSSASWSSNNNPTLKAEEDEEDQDIANCLILLSQGH  104
BnaAZFP7        ---ENDVAN--RKKDGVITS---SSSSASWSSNNNPTLKAEEDEEDQDIANCLILLSQGH  104
BoZFP11         ---ENDVAN--RKKDGVVTS---SSSSASWSSNNNPTLKGEEDEEDQDVANCLILLSQGH  104
BnaCZFP7        ---ENDVAN--RKKDGVVTS---SSSSASWSSNNNPTLKGEEDEEDQDVANCLILLSQGH  104
BrZFP34         --------E--SKKDGVITS---SSSSASWFSNNNATLKAEEDEEEQDIANCLILLSQGH  97
BnaAZFP33       --------E--SKKDGVITS---SSSSASWFSNNNATLKAEEDEEEQDIANCLILLSQGH  97
BoZFP35         --------E--SKKDGVITS---SSSSASWSSNNNATLKAEEDEEEQEIANCLILLSQGH  98
BnaCZFP31       --------E--SKKDGVITS---SSSSASWSSNNNATLKAEEDEEEQEIANCLILLSQGH  98
BoZFP7          NALANNVGN--HKKDGVITS---SSSSASWSSNNNPTLKAEEDEEDLDIASCLILLSQGH  114
BrZFP5          NALGNNVEN--HKKDGVITS---SSSSASWSSNNNPTLKAEEDEEDLDIASCLILLSRGH  114
BnaAZFP37       NALGNNVEN--HNKDGVITS---SSSSASWSSNNNPTLKAEEDEEDLDIASCLILLSRGH  114
BrZFP4          EG-------------------------------AGEVEFQGATDEDQDMANCLMLLSQGH  76
BnaAZFP36       EG-------------------------------AGEVEFQGATDEDQDMANCLMLLSQGH  76
BoZFP6          EE-------------------------------AGEVEFQGATDEDQDMANCLMLLSQGH  76
BnaCZFP5        EE-------------------------------ADEVEFQGATDEDQDMANCLMLLSQGH  76
BoZFP37         EG-------------------------------AGEVEFQGATEEDQDMANCLMLLSQGH  77
BnaAZFP12       EG-------------------------------AGEVEFQGATEEDQDMANCLMLLSQGH  77
BnaCZFP35       EG-------------------------------AGEVEFQGATEEDQDMANCLMLLSQGH  77
BrZFP35         EG-------------------------------AGEVEFQGATEEDQDMANCLMLLSQGH  77
BnaAZFP35       DG-------------------------------AGEIEFQGATEEDQDMANCLMLLSQGH  77
BoZFP10         EG-------------------------------AGEIEFRGATDEDQDMANCLMLLSQGH  77
BnaCZFP8        EG-------------------------------AGEIEFRGATDEDQDMANCLMLLSQGH  77
BrZFP8          EG-------------------------------VGEIEFRGATDEDQDMANCLMLLSQGH  76
BnaAZFP6        EG-------------------------------VGEIEFRGATDEDQDMANCLMLLSQGH  76
BrZFP15         GGGGRAV------------S------DEYNSAVSSPVTTTDCTEEEEDMAICLIMLARGA  91
BoZFP20         GGGGRAV------------S------DEYNSAVSSPVTTTDCTEEEEDMAICLIMLARGA  91
BnaCZFP32       GGGGRAV------------S------DEYNSAVSSPVTTTDCTEEEEDMAICLIMLARGA  91
BnaAZFP14       GGGGRAV------------S------DEYNSAVSSPVTTTDCTEEEEDMAICLIMLARGA  91
BoZFP19         AGGERTA------------S------DEYNSVVSSPVTTTDCTEEEEAMAICLIMLARGA  88
BnaCZFP16       ADGERTA------------S------DEYNSVVSSPVTTTDCTEEEEDMAICLIMLARGA  88
BrZFP25         AGGERTA------------S------EEYNSVVSSPVTTTDCTEEEEDMAICLIMLARGA  88
BnaAZFP26       AGGERTA------------S------EEYNSVVSSPVTTTDCTEEEEDMAICLIMLARGA  88
                                                           :*:  :* **::*::*
```

```
BoZFP27        YTPQQ------QPQQTRQFMMSYQESGNNN-NNAYRSSSRRFLETS--NGT-TSGGRAGY 165
BnaCZFP24      YTPQQ------QPQQTRQFMMSYQESGNNN-NNAYRSSSRRFLETS--NGT-TSGGRAGY 165
BrZFP18        YTPQQ------QPQQTRQFMMSYQESGNNN-NNAYRSSSRRFLETSSPNGT-TSGGRAGY 167
BnaAZFP19      YTPQQ------QPQQTRQFMMSYQESGNNN-NNAYRSSSRRFLETSSPNGT-TSGGRAGY 167
BoZFP4         SLPHHNHNHNHQQPQTRQLMVSYQESGNNNSSNAYRSSSRRFLETSSSNGTTTSGGRAGY 178
BrZFP3         SLPH-------QQPQTRQLMVSYQESGNNN-NNAYRSSSRRFLETSSSNGTTTSGGRAGY 170
BnaAZFP3       SLPH-------QQPQTRQLMVSYQESGNNN-NNAYRSSSRRFLETSSSNGTTTSGGRAGY 170
BrZFP9         SFPQHNQQLKI----------PH--QEIN-NNNTYRFSSRRFLETSSSNG----GGKSGY 147
BnaAZFP7       SFPQHNQQLKI----------PH--QEIN-NNNTYRFSSRRFLETSSSNG----GGKSGY 147
BoZFP11        SFPQHNQQLKI----------PH--QEIN-NNNTYRFSSRRFLETSSSNG----GGKSGY 147
BnaCZFP7       SFPQHNQQLKI----------PH--QEIN-NNNTYRFSSRRFLETSSSNG----GGKSGY 147
BrZFP34        SLP-------I----------PN--HEAN-NNNTFRFSSRRFLETSSSNG----GGKAGY 133
BnaAZFP33      SLP-------I----------PN--HEAN-NNNTFRFSSRRFLETSSSNG----GGKAGY 133
BoZFP35        SLP-------L----------PN--HEAN-NNNTYRFSSRRFLETSSSNG----GDKAGY 134
BnaCZFP31      SLP-------L----------PN--HEAN-NNNTYRFSSRRFLETSSSNG----GDKAGY 134
BoZFP7         SLPQ----LKI----------PN--HETN-NNNTYKFSSRRFLETSSSNG----GGKAGH 153
BrZFP5         SLPQ----LKI----------PN--HETTYNNKTYKFSSRRFLETSSSNG----GGKAGY 154
BnaAZFP37      SLPQ----LKI----------PN--HETTYNNKTYKFCSRRFLETSSSNG----GGKAGY 154
BrZFP4         KEKSSGDHSSTL---------------------KI-----GFLT---DKKPVASLGLGLD 107
BnaAZFP36      KEKSSGDHSSTL---------------------KI-----GFLT---DKKPVASLGLGLD 107
BoZFP6         NAKSSGDHLSTQ---------------------KT-----GFLS---DKKPVGSLGLGLD 107
BnaCZFP5       KAKSSGDHLSTQ---------------------KT-----GFLS---DKKPVGSLGLGLD 107
BoZFP37        KENGSGDHSSKH---------------------KL-----DFLS---YKKPAASLGLGLE 108
BnaAZFP12      KANGSGDHSSTH---------------------KI-----DFLC---KKKPVASLGLGLE 108
BnaCZFP35      KANGSGDHSSTH---------------------KI-----DFLC---KKKPVASLGLGLE 108
BrZFP35        KANGSGDHSSTH---------------------KL-----DFLC---NKKPVASLGLGLE 108
BnaAZFP35      KANGSGDHSSTH---------------------KL-----DFLS---YKNPAASLGLGLE 108
BoZFP10        KSNTSGDPLLTH---------------------KI-----GFLS---NKKPVASLVLGLN 108
BnaCZFP8       KSNTSGDPLLTQ---------------------KI-----GFLS---NKKPVASLGLGIN 108
BrZFP8         KSNTSGDPLLTQ---------------------KI-----GFLS---NKKPVASLGLGLN 107
BnaAZFP6       KSNTSGDPLLTQ---------------------KI-----GFLS---NKKPVASLGLGLN 107
BrZFP15        ALSPDL---KNS---------------------RK-----ADKT-------LSSAENSSF 115
BoZFP20        ALSPDL---KNS---------------------RK-----ADKT-------LSSAENSSF 115
BnaCZFP32      ALSPDL---KNS---------------------RK-----ADKT-------LSSAENSSF 115
BnaAZFP14      ALSPDL---KNS---------------------RK-----ADKT-------FSPAENSSF 115
BoZFP19        APSPPLPDLKNS---------------------TK-----TDKN---LHLKTSSTENSSF 119
BnaCZFP16      APSPPLPDLKNS---------------------TK-----TDKN---LHLKTSSSENSSF 119
BrZFP25        APSPPLPDLKNS---------------------TK-----TDKN---LYQ-----KNSSF 114
BnaAZFP26      APSPPLPDLKNS---------------------TK-----TDKN---LYQ-----KNSSF 114
.
```

```
                ------------ZF#1-----------
BoZFP27        YVVYQCKTCDRTFPSFQALGGHRASHKKPKAAAGLHSDH---DLKKSIYNDAVSLHLNNVP 222
BnaCZFP24      YVVYQCKTCDRTFPSFQALGGHRASHKKPKAAAGLHSDH---DLKKSIYNDAVSLHLNNVP 222
BrZFP18        YVVYQCKTCDRTFPSFQALGGHRASHKKPKAAAGLHSDH---DLKKSIYNDAVSLHLNNVP 224
BnaAZFP19      YVVYQCKTCDRTFPSFQALGGHRASHKKPKAAAGLHSDH---DLKKSIYNDAVSLHLNNVP 224
BoZFP4         YVVYQCKTCDRTFPSFQALGGHRASHKKPKAAPGLH------ELKKSIYNDAVSLHLNDVL 232
BrZFP3         YVVYQCKTCDRTFPSFQALGGHRASHKKPKAAPGLH------DLKKSIYNDAVSHHLNNVL 224
BnaAZFP3       YVVYQCKTCDRTFPSFQALGGHRASHKKPKAAPGLH------DLKKSIYNDAVSHHLNNVL 224
BrZFP9         YVVYQCKTCDRTFPSFQALGGHRASHKKPRATSF-YSNL---DVKKSIYENDAASLT---N 200
BnaAZFP7       YVVYQCKTCDRTFPSFQALGGHRASHKKPRATSF-YSNL---DVKKSIYENDAASLT---N 200
BoZFP11        YVVYQCKTCDRTFPSFQALGGHRASHKKPRATSF-YSNL---DLKKSIYENDAASLVTTTN 203
BnaCZFP7       YVVYQCKTCDRTFPSFQALGGHRASHKKPRATSF-YSNL---DLKKSIYENDAASLVTTTN 203
BrZFP34        YVVYQCKTCDRTFPSFQALGGHRASHKKPKATLSLYSNI---DVKKNIYESDAVSLVTTST 190
BnaAZFP33      YVVYQCKTCDRTFPSFQALGGHRASHKKPKATLSLYSNI---DVKKNIYESDAVSLVTTST 190
BoZFP35        YVVYQCKTCDRTFPSFQALGGHRASHKKPKATLSSYSNI---DVKKNIYESNAVSLVTTST 191
BnaCZFP31      YVVYQCKTCDRTFPSFQALGGHRASHKKPKATLSSYSNI---DVKKNIYESNAVSLVTTST 191
BoZFP7         YVVYQCKTCDRTFSSFQALGGHRASHKKPKATSFYSNLD---HLKKNIYENDS--LATTTT 208
BrZFP5         YVVYQCKTCDRTFSSFQALGGHRASHKKPKATSFYSNLE---HLKKNIYENDS--LATTTT 209
BnaAZFP37      YVVYQCKSCDRTFSSFQALGGHRASHKKPKATSFYSNLD---HLKKNIYENDS--LSTTTT 209
BrZFP4         GVYQCKTCDKSFHSFQALGGHRASHKKPKR-----------DEKNSASAVETVESAEAVG 156
BnaAZFP36      GVYQCKTCDKSFHSFQALGGHRASHKKPKR-----------DEKNSASAVETVESAEAVG 156
BoZFP6         GVYPCKTCDKSFHSFQALGGHRASHKKSKR-----------DEKNSASAVETVESAEAVG 156
BnaCZFP5       GVYPCKTCDKSFHSFQALGGHRASHKKSKR-----------DEKNSASAVETVESAETVG 156
BoZFP37        GVYQCQTCDKSFHSFQALGGHRASHKKPKLGAIVLKC----HEKKSSSASAV---ETAKG 161
BnaAZFP12      GVYQCQTCDKSFHSFQALGGHRASHKKPKLGENVLKC----HEKKSSSAFAV---ETAKG 161
BnaCZFP35      GVYQCQTCDKSFHSFQALGGHRASHKKPKLGENVLKC----HEKKSSSASAV---ETAKG 161
BrZFP35        GVYQCQTCDKSFHSFQALGGHRASHKKPKLGAIVLKC----HEKKSSSASAV---ETAKG 161
BnaAZFP35      GVYQCQTCDKSFHSFQALGGHRASHKKPKLGAIVLKC----HEKKSSSAFAV---ETAKG 161
BoZFP10        GVYQCKTCDKSFHSFQALGGHRTSHKKPKLGAIVLKC----DEKKSASAVKTVEAARVVG 164
BnaCZFP8       GVYQCKTCDKSFHSFQALGGHRTSHKKPKLGAIVLKC----DEKKSASAVKTVEAARVVG 164
BrZFP8         GVYQCKTCDKSFHSFQALGGHRTSHKKPKLGAIILKC----GEKKSASAVKTVEAARVVG 163
BnaAZFP6       GVYQCKTCDKSFHSFQALGGHRTSHKKPKLGAIILKC----GEKKSASAVKTVEAARVVG 163
BrZFP15        FVYECKTCSRTFSSFQALGGHRASHKKPRVSIEAKTKLPLMQAKSSGSEEGQKSNFKVFG 175
BoZFP20        FVYECKTCSRTFSSFQALGGHRASHKKPRVSIEEKTKLPLMQAKSSGSEEGQKYNFKVFG 175
BnaCZFP32      FVYECKTCSRTFSSFQALGGHRASHKKPRVSIEEKTKLPLMQAKSSGSEEGQKYNFKVFG 175
BnaAZFP14      FVYECKTCSRTFSSFQALGGHRASHKKPRVSIEEKTKLPLMQAKSSGSEEGQKNNFKVFG 175
BoZFP19        YVYECKTCNRTFPSFQALGGHRASHKKPRASIDEKAKVPLTQLKSSASEEGQKSHFKVSG 179
BnaCZFP16      YVYECKTCNRTFPSFQALGGHRASHKKPRASIDEKAKVPLTQLKSSASEEGQKSHFKVSG 179
BrZFP25        YVYECKTCNRTFPSFQALGGHRASHKKPRASIDEKAKVPLTQLKSSASEEGQKGHFKVSG 174
BnaAZFP26      YVYECKTCNRTFPSFQALGGHRASHKKPRASIDEKAKVPLTQLKSSASEEGQKGHFKVSG 174
                ** *::*.::* *********:**** :              *..
```

```
                                       --------ZF#2------------
BoZFP27        AATPNNNSSHRSLVVYGKANNNKVHECGICGAEFTSGQALGGHMRRHRGAVVASAASAST 282
BnaCZFP24      AATPNNNSSHRSLVVYGKANNNKVHECGICGAEFTSGQALGGHMRRHRGAVVASAASAST 282
BrZFP18        TATPNNNSSHRSLVVYGKAGNNKVHECGICGAEFTSGQALGGHMRRHRGAVVASAASAST 284
BnaAZFP19      TATPNNNSSHRSLVVYGKAGNNKVHECGICGAEFTSGQALGGHMRRHRGAVVASAASAST 284
BoZFP4         TTTPNNSSNHRSLVVYGKANNNKVHECGICGAEFTSGQALGGHMRRHRGAGVAATAT-PT 291
BrZFP3         TTTPNNNNNHRSLVVHGKANNNKVHECGICGAEFTSGQALGGHMRRHRGAGVAATAT-PT 283
BnaAZFP3       TTTPNNNNNHRSLVVHGKANNNKVHECGICGAEFTSGQALGGHMRRHRGAGVAATAT-PT 283
BrZFP9         IY-NNKNNNNRSLVAYGKAGNNKVHECGICGAEFTSGQALGGHMRRHRGAVVVAAAPAPI 259
BnaAZFP7       IY-NNKNNNNRSLVAYGKAGNNKVHECGICGAEFTSGQALGGHMRRHRGAVVVAAAPAPI 259
BoZFP11        IYNNNKNNNNRSLVAYGKANNNKVHECGICGAEFTSGQALGGHMRRHRGAVVVAAAPAPI 263
BnaCZFP7       IYNNNKN-NNRSLVAYGKANNNKVHECGICGAEFTSGQALGGHMRRHRGAVVVAAAPAPI 262
BrZFP34        IYKN---NNNRSLAVYGKAGSNKVHECGVCGAEFTSGQALGGHMRRHRGAVVIAAAPVTT 247
BnaAZFP33      IYKN---NNNRSLAVYGKAGSNKVHECGVCGAEFTSGQALGGHMRRHRGAVVIAAAPVTT 247
BoZFP35        IYKN---NNNRSLAVYGKAGSNKVHECGVCGAEFTSGQALGGHMRRHRGAVVIAAAPVTT 248
BnaCZFP31      IYKN---NNNRSLAVYGKAGSNKVHECGVCGAEFTSGQALGGHMRRHRGAVVIAAAPVTT 248
BoZFP7         IYNN---NKNRSLVVYGKAGNNKVHECGICGEEFTSGQALGGHMRRHRGAVVVAP----T 261
BrZFP5         IYNN---NKNRSLVVYGKAGNNKVHKCGICGAEFTSGQALGGHMRRHRGAVVVAP----T 262
BnaAZFP37      IYNN---NKNRSLVVYGKAGNNKVHECGICGAEFTSGQALGGHMRRHRGAVVVAP----T 262
BrZFP4         SFL---SL--QVTSSDGSKKPEKTHECSICKAEFSSGQALGGHMRRHRGLNVNANATSIT 211
BnaAZFP36      SFL---SL--QVSSSDGSKKPEKTHECSICKAEFSSGQALGGHMRRHRGLNVNANATSIT 211
BoZFP6         SFL---SL--QVTSSDGSKKPEKTHECSICKAEFSSGQALGGHMRRHRGLNVNANATSIT 211
BnaCZFP5       SFL---SL--QVTSSDGSKKPEKTHECSICKAEFSSGQALGGHMRRHRGLNVNANATSIT 211
BoZFP37        SFL---SL--QVTSSDGSKKPEKTHECSICKAEFSSGQALGGHMRRHRGLIVNANAT--- 213
BnaAZFP12      SFL---SL--QVTSSDGNKKPEKTHECSICKAEFSSGQALGGHMRRHRGLIVNANAT--- 213
BnaCZFP35      SFL---SL--QVTSSDGSKKPEKTHECSICKAEFSSGQALGGHMRRHRGLIVNANAT--- 213
BrZFP35        SFL---SL--QVTSSDGNKKPEKTHECSICKAEFSSGQALGGHMRRHRGLIVNANAT--- 213
BnaAZFP35      SFL---SL--QVTSSDGNKKPEKTHECSICKAEFSSGQALGGHMRRHRGLIVNANAT--- 213
BoZFP10        SFL---SL--QVTSSDGSKKQEKTHECSICKAEFSSGQALGGHMRRHRGLTLNANANSTI 219
BnaCZFP8       SFL---SL--QVTSSDGSKKQEKTHECSICKAEFSSGQALGGHMRRHRGLTLNANANSTI 219
BrZFP8         SFL---SL--QVTSSEGTKKQEKTHECSICKAEFSSGQALGGHMRRHRGLTLNANANSTT 218
BnaAZFP6       SFL---SL--QVTSSEGTKKQEKTHECSICKAEFSSGQALGGHMRRHRGLTLNANANSTT 218
BrZFP15        SSL---AS---LSSNIIISKANKVHECSICGSEFTSGQALGGHMRRHRTATTAV-IPVST 228
BoZFP20        SSL---AS---LSSNIIISKANKVHECSICGSEFTSGQALGGHMRRHRTATTAV-IPVAT 228
BnaCZFP32      SSL---AS---LSSNIIISKANKVHECSICGSEFTSGQALGGHMRRHRTATTAV-IPVAT 228
BnaAZFP14      SSL---AL---LSSNIIISKANKVHECSICGSEFTSGQALGGHMRRHRTATTAV-IPVAT 228
BoZFP19        PAL---AS---KASNIIISKANKVHECSICGSEFTSGQALGGHMRRHRTVTNVVSSPVST 233
BnaCZFP16      PAL---AS---KASNIIISKANKVHECSICGSEFTSGQALGGHMRRHRTVTNVVSSPVST 233
BrZFP25        PAL---AS---KASNIIISKANKVHECSICGSEFTSGQALGGHMRRHRTVTNVVSSPVTA 228
BnaAZFP26      PAL---AS---KASNIIISKANKVHECSICGSEFTSGQALGGHMRRHRTVTNVVSSPVTA 228
                                    :*.*:*.:*  **:*************
```

```
                                                               ---EAR---
BoZFP27        ATVRVAATAGTANTALSLSPMSFDQMS---VHPVQAPVKRARSAVVSLDLDLNLPAS--E 337
BnaCZFP24      ATVRVAATAGTANTALSLSPMSFDQMS---VHPVQAPVKRARSAVVSLDLDLNLPAS--E 337
BrZFP18        ATVRVAATAGTANTALSLSPMSFDHMS---VHPVQGPVKRARSAVVSLDLDLNLPAP--E 339
BnaAZFP19      ATVRVAATAGTANTALSLSPMSFDHMS---VHPVQGPVKRARSAVVSLDLDLNLPAP--E 339
BoZFP4         ATLALPASAATANTVLSLSPMSFDQMSDGPVYPVQAPVKRARSAVVSLDLDLNLPAP--E 349
BrZFP3         ATLALPASAATANTVLSLSPMSFDQLSDGPVYPVQAPVKRARSAVVSLDLDLNLPAP--E 341
BnaAZFP3       ATLALPASAATANTVLSLSPMSFDQLSDGPVYPVQAPVKRARSAVVSLDLDLNLPAP--E 341
BrZFP9         VTVA----AAAANTELSLSSMSYDQISEGQDHLVMPEAKKAKKMVVSLDLDLNLPAP--E 313
BnaAZFP7       VTVA----AAAANTELSLSSMSYDQISEGQDHLVMPEAKKAKKMVVSLDLDLNLPAP--E 313
BoZFP11        VTVA----AAAANTELSLSSMSYDQISDGQDHLVMPEAKKAKKMVVSLDLDLNLPAP--E 317
BnaCZFP7       VTVA----AAAANTELSLSSMSYDQISDGQDHLVMPEAKKAKKMVVSLDLDLNLPAP--E 316
BrZFP34        VTVA----TAAANTELSLSSMSFDQISDGQDHLVMPATKRAKKTVVSLDLDLNLPAS--E 301
BnaAZFP33      VTVA----TAAANTELSLSSMSFDQISDGQDHLVMPATKRAKKTVVSLDLDLNLPAS--E 301
BoZFP35        VTVA----TAAANTELSLSSMSFDQISDGQDHLVMPATKRAKKTVVSLDLDLNLPAP--E 302
BnaCZFP31      VTVA----TAAANTELSLSSMSFDQISDGQDHLVMPATKRAKKTVVSLDLDLNLPAP--E 302
BoZFP7         VTVT----LAAANTELSLSSMSFDQIS----------TKRAKKMVVSLDLDLNLPAPEY- 306
BrZFP5         VTVA----LAAANTELSLSSMSFDQIS----------TKRAKKMVVSLDLDLNLPALELE 308
BnaAZFP37      VTVA----LAAANTELSLSSMSFDQIS----------TKRAKKMVVSLDLDLNLPAP--E 306
BrZFP4         K------AV------LSSSH-----------------HQEPIRPKNFLELDLNLPAP--E 240
BnaAZFP36      K------AV------LSSSH-----------------HQEPIRPKNFLELDLNLPAP--E 240
BoZFP6         K------AL------LSSSH-----------------HQEPIRPKNFLELDLNLPAP--E 240
BnaCZFP5       K------AL------LSSSH-----------------HQEPIRPKNFLELDLNLPAP--E 240
BoZFP37        -----------------SSH-----------------HQESIRPKNFLELDLNLPAP--E 237
BnaAZFP12      -----------------SSH-----------------HQESIRPKNFLELDLNLPAP--E 237
BnaCZFP35      -----------------SSH-----------------HQESIRPKNFLELDLNLPAP--E 237
BrZFP35        -----------------SSH-----------------HQESIRPKNYLELDLNLPAP--E 237
BnaAZFP35      -----------------SSH-----------------HQESIRPKNYLELDLNLPAP--E 237
BoZFP10        R------TA------TSSSH-----------------NQESIRENNFMELDLNLPAP--E 248
BnaCZFP8       R------TA------TSSSH-----------------NQESIRENNFMELDLNLPAP--E 248
BrZFP8         R------TE------ISSSH-----------------HQESIREKNFIELDLNLPAP--E 247
BnaAZFP6       R------TE------ISSSH-----------------HQESIREKNFIELDLNLPAP--E 247
BrZFP15        TEVSRNSTE------EETEN-----------------LSSYIEQRKYLPLDLNLPAP--E 263
BoZFP20        TEVSRNSTE------EETEN-----------------LSSYIEQRKYLPLDLNLPAP--E 263
BnaCZFP32      TEVSRNSTE------EETEN-----------------LSSYIEQRKYLPLDLNLPAP--E 263
BnaAZFP14      TEVSRNSTE------EETEN-----------------LSSYIEQRKYLPLDLNLPAP--E 263
BoZFP19        AEVSRNSTE------EETEN-----------------LSRSMEQRKYLPLDLNLPAP--E 268
BnaCZFP16      AEVSRNSTE------EETEN-----------------LSRSMEQRKYLPLDLNLPAP--E 268
BrZFP25        AEVSRNSTD------EETEN-----------------LSRSMEQRKYLPLDLNLPAP--E 263
BnaAZFP26      AEVSRNSTD------EETEN-----------------LSRSMEQRKYLPLDLNLPAP--E 263
                                                              : *******
```

```
BoZFP27         DVNRVNGLSFASKQEQEQEHEHEQTHQREEQKTLVL-SSAPTLVDCHY-----        384
BnaCZFP24       DVNRVNGLSFASKQEQEQEHEHEQTHQREEQKTLVL-SSAPTLVDCHY-----        384
BrZFP18         DVNRVNGLSFASKQEQ--EHEHEQTQQREEQKSIVL-SSAPTLVDCHY-----        384
BnaAZFP19       DVNRVNGLSFASKQEQ--EHEHEQTQQREEQKSIVL-SSAPTLVDCHY-----        384
BoZFP4          DENRVNGLSVAPKQEHEQ--EHGQTQRREEQKSIVL-SSAPTLVDCYY-----        394
BrZFP3          DENRVNGLSVASKQEHG----HEQTQGREEQKSIVL-SSAPTLVDCYY-----        384
BnaAZFP3        DENRVNGLSVASKQEHG----HEQTQGREEQKSIVL-SSAPTLVDCYY-----        384
BrZFP9          DENRVNGLSLTLKQKHEQEQEHQQTKQREEQVSLVL--SAPTLVDCYY-----        359
BnaAZFP7        DENRVNGLSLTLKQKHEQEQEHQQTKQREEQVSLVL--SAPTLVDCYY-----        359
BoZFP11         DENRVNGLSLALKQKHEQEQEHQETKQKEEPVSLVL--SAPTLVDCYY-----        363
BnaCZFP7        DENRVNGLSLALKQKHEQEQEHQETKQKEEPVSLVL--SAPTLVDCYY-----        362
BrZFP34         DENRVNGFTFALKQKQE--QEHQPTMQREEPKCLLM--SAPTLVDCRY-----        345
BnaAZFP33       DENRVNGFTFALKQKQE--QEHQPTMQREEPKCLLM--SAPTLVDCRY-----        345
BoZFP35         DENRVNGFTFALKQKQE--QEHQPTMQREEPKCLLM--SAPTLVDCHY-----        346
BnaCZFP31       DENRVNGFTFALKQKQE--QEHQPTMQREEPKCLHM--SAPTLVDCHY-----        346
BoZFP7          -ENRVNGFSLCFKQKHE--QEHQQTKQRDEPKCLVL--SPPTLVDCHY-----        349
BrZFP5          DENMVNGFSLGFKQKHE--QEHQQTKQRDEPKCLVL--SPPTSVDCHY-----        352
BnaAZFP37       DENRVNGFSLGFKQKHE--QEHQQTKQRDEPKCLVL--SPPTLVDCHY-----        350
BrZFP4          DEPKF---VFASK---------------DQMLLFVS-ASNSLIDCHH-----         268
BnaAZFP36       DEPKF---VFASK---------------DQMLLFAS-ASNSLIDCHH-----         268
BoZFP6          DEPKF---VFASK---------------DQMLLFAS-ASNSLIDCHH-----         268
BnaCZFP5        DEPKF---VFASK---------------DQMLLFAS-ASNSLIDCHH-----         268
BoZFP37         DESKF---VFASK---------------DQIILFTT-ASNSLIDCHH-----         265
BnaAZFP12       DESKF---VFASK---------------DQIILFTT-ASNSLIDCHH-----         265
BnaCZFP35       DESKF---VFASK---------------DQIILFTT-ASNSLIDCHH-----         265
BrZFP35         DESKF---VFASK---------------DQIILFTT-ASNSLIDCHH-----         265
BnaAZFP35       DESKF---VFASK---------------DQIILFTT-ASNSLIDCHH-----         265
BoZFP10         DEPKF---VFASK---------------DQIILFAA-ASNSLIDCHH-----         276
BnaCZFP8        DEPKF---VFASK---------------DQIILFTA-ASNSLIDCHH-----         276
BrZFP8          DEPKF---VFASK---------------DQILLFAAAASNSLIDCHH-----         276
BnaAZFP6        DEPKF---VFASK---------------DQILLFAAAASNSLIDCHH-----         276
BrZFP15         DDLRE------SK---------------FQGIVFST-T-PALIDHYKKTAVF         292
BoZFP20         DDLRE------SK---------------FQGIVFST-T-PALIDCHY-----         287
BnaCZFP32       DDLRE------SK---------------FQGIVFST-T-PALIDCHY-----         287
BnaAZFP14       DDLRE------SK---------------FQGIVFST-T-PALIDCHY-----         287
BoZFP19         DDLRE------SK---------------FQGVVFSA-TTPALIDCHY-----         293
BnaCZFP16       DDLRE------SK---------------FQRIVFSA-TTPALIDCHY-----         293
BrZFP25         DDLRE------SK---------------FQGIVFSA-TTPALIDCHY-----         288
BnaAZFP26       DDLRE------SK---------------FQGIVFSA-TTPALIDCHY-----         288
                            *                    :    :  : :*
```

```
2i-D Brassica ZFPs                                  ----NLS---------
BoZFP26        MALEAMNCPTNSSSFTARKDRNEPT-DDLTNDAVFMEPWLKRKRSKRQRSGSPSSSS--S 57
BnaCZFP23      MALEAMNCPTNSSSFTAREDRNEPT-DDLTNDAVFMEPWLKRKRSKRQRSGSPS-----S 54
BrZFP20        MALEAMNCPTNSSSFTARKDRNEPT-DDLTNDTVFMEPWMKRKRSKRQRSRSPS-----S 54
BnaAZFP18      MALEAMNCPTNSSSFTARKDRNEPT-DDLTHDAVFMEPWLKRKRSKRPRSRSPS-----S 54
BoZFP5         MALEAMNSP----SFTVRKDRIEATEDDLMNDAVFMEPWLKRKRSKRQRSRSPSPSTS-S 55
BnaCZFP4       MALEAMNSP----SFTVRKDRIEATEDDLMNDAVFMEPWLKRKRSKRQRSRSPSPSTS-S 55
BrZFP2         MALEAMNSP----SFTVRKDRIEATNDDLTNDAVFMEPWLKRKRSKRQRSRSPSPSTSSS 56
BnaAZFP2       MALEAMNSP----SFTVRKDRIAATDDDLTNDAVFMEPWLKRKRTKRQRSRSPSPSTSSS 56
BoZFP13        MALEAMNSPTTMSSFTVRKDRIGAT-DDLMNDAVFLEPWLKRKRSKRQRSPSPSS----- 54
BnaCZFP9       MALEAMNSPTTMSSFTVRKDRIGAT-DDLMNDAVFLEPWLKRKRSKRQRSPSPSS----- 54
BrZFP12        MALEAMNSPTTTSPFTVRKDRIGAT-DDLMNDAVFLEPWLKRKRSKRQRSPSPSS----S 55
BnaAZFP10      MALEAMNSPTTTSPFTVRKDRIGAT-DDLMNDAVFLEPWLKRKRSKRQRSPSPSS----S 55
BoZFP30        MALEALNSPRIASPVPTLFEE----------------HWTKGKRSKRSRSDHP------- 37
BnaCZFP27      MALEALNSPRIASPVPTLFEE----------------HWTKGKRSKRSRSDHP------- 37
BrZFP24        MALEALNSPRIASPVPTLFEE----------------HWTKGKRSKRSRSDHL------- 37
BnaAZFP24      MALEALNSPRIASPVPTLFEE----------------HWTKRKRSKRSRSDHL------- 37
BoZFP36        MALEALSSPRLASPVPTLFQDYAV-------GFH----GSKGKRSKRSRSEFD------- 42
BnaCZFP34      MALEALSSPRLASPVPTLFQDYAV-------GFH----GSKGKRSKRSRSEFD------- 42
BnaAZFP34      MALEALSSPRLASPVPTLFQDSAV-------GFH----GSKGKRSKRSRSEFD------- 42
BoZFP29        MALEALSSPRLASPIPPVFQDSSR--------FHGVEQWTKGKRSKRSRFDFN------- 45
BnaCZFP26      MALEALSSPRLASPIPPVFQDSSR--------FHGVEQWTKGKRSKRSRFDFN------- 45
BrZFP30        MALEAISSPRLASPVPPLLEDSSR--------FHGVDHWTKGKRSKRSRSDFH------- 45
BoZFP25        MALEAISSPRLASPVPPLFEDSSR--------FHGVEHWTKGKRSKRSRSDFP------- 45
BnaCZFP22      MALEAISSPRLASPVPPLFEDSSR--------FHGVEHWTKGKRSKRSRSDFP------- 45
BnaAZFP30      MALEAISSPRLASPVPPLFEDSSR--------FHGVDHWTKGKRSKRSRSDFH------- 45
BoZFP16        MALEALSSPRLASPVPPLFEDSSR--------FHGVEHWTKGKRSKRSRSDFH------- 45
BnaCZFP13      MALEALSSPRLASPVPPLFEDSSR--------FHGVEHWTKGKRSKRSRSDFH------- 45
BrZFP28        MALEALSSPRLASPVPPLFEDSSR--------FHGVEHWTKGKRSKRSRSDFN------- 45
BnaAZFP28      MALEALSSPRLASPVPPLFEDSSR--------FHGVEHWTKGKRSKRSRSDFN------- 45
BoZFP14        MALETLNSPTSATATALPLLRYRE-----EMEPDNLEQWAKRKRTKRQRLDQN------- 48
BnaCZFP11      MALETLNSPTSATATALPLLRYRE-----EMEPDNLEQWAKRKRTKRQRLDQN------- 48
BrZFP23        MALETLNSPTSATATARPLLRYRE-----EMEPDNLEQWAKRKRTKRQRIDQN------- 48
BnaAZFP23      MALETLNSPTSATATARPLLRYRE-----EMEPDNLEQWAKRKRTKRQRLDQN------- 48
BoZFP9         MALETLNSPSSATASARPLLRYRE-----EMEPENLEQWAKRKRTKRQRFDQS------- 48
BnaCZFP6       MALETLNSPSSATASARPLLRYRE-----EMEPENLEQWAKRKRTKRQRFDQS------- 48
BrZFP7         MALETLNSPTSATASARPLLRYRE-----EMEPENLEQWAKRKRTKRQRFDQS------- 48
BnaAZFP38      MALETLNSPTSATASARPLLRYRE-----EMEPENLEQWAKRKRTKRQRFDQS------- 48
BrZFP29        MALETLNSPTSATATARPFLRYRE-----EMEPENLEQWAKRKRTKRQRFDHN------- 48
BnaAZFP29      MALETLNSPTSATATARPFLRYRK-----EMEPENLEQWAKRKRTKRQRFDHN------- 48
BoZFP33        MVLETLNSPTSATAAARPFLRYRE-----EMEPENLEQWAKRKRTKRQRFDHN------- 48
BnaCZFP29      MALETLNSPTSATAAARPFLRYRE-----EMEPENLEQWAKRKRTKRQRFDHN------- 48
BrZFP1         MALDTLNSPNSTTTAPSPFLT----------EPENLEPWTKRKRTKRHRIDD-------- 42
BnaAZFP1       MALDTLNSPNSTTTAPSPFLT----------EPENLEPWTKRKRTKRHRIDD-------- 42
BoZFP3         MALDTLNSPNSTTTAPSPFLT----------EPENLEPWTKRKRTKRHRIDD-------- 42
BnaCZFP2       MALDTLNSPNSTTTAPSPFLT----------EPENLEPWTKRKRTKRHRIDD-------- 42
BnaCZFP3       MALDTLNSPNSTTTAPSPFLT----------EPENLEPWTKRKRTKRHRIDD-------- 42
BrZFP13        MALDTLNSPTST-TAPPPFLT----------EPENLESWTKRKRTKRHRTVD-------- 41
BnaAZFP11      MALDTLNSPTST-TAPPPFLT----------EPENLESWTKRKRTKRHRTVD-------- 41
BoZFP31        MALDTLNSPTST-TAPPPFLT----------KPENLESWTKRKRTKRHRTVD-------- 41
BnaCZFP28      MALDTLNSPTST-TAPPPFLT----------KPENLESWTKRKRTKRHRTVD-------- 41
               *.*:::..*                              * **:** *
```

```
                              ----L-box----                        ---
BoZFP26        SPPR-SRPKSQIQDLAEEEYLALCLLMLANNHHQPKRR----------PQESTTKLSHKC  106
BnaCZFP23      SPPR-SRPKSQIQDLAEEEYLALCLLMLANNHHQPKRR----------PQESTTKLSHKC  103
BrZFP20        SPPR-SRPKSQIQDLAEEEYLALCLLMLANDHHQPKTQ---------PPQESTTKLSHKC  104
BnaAZFP18      SPPR-SRPKSQIQDLAEEEYLALCLIMLANDHHQPKTQ---------PPQESTTKLSHKC  104
BoZFP5         SPPRSRRPKSESQDLTEEEYLALCLLKLAKDKHSPRP-----------QPQDSTKLSYKC  104
BnaCZFP4       SPPRSRRPKSESQDLTEEEYLALCLLKLAKDKHSPRP-----------QPQDSTKLSYKC  104
BrZFP2         SPPRSRRPKYESQDLTEEEYLALCLLKLAKDKHSPPP---------QPQPRDSTKLSYKC  107
BnaAZFP2       SPPRSRRPKSESQDLTEEEYLALCLLKLAKDKHSPPP---------QPQPRDSTKLSYKC  107
BoZFP13        SPPR-SHAKSQSQDPSEEEYLALCLLMLAKDQPRTRFQPSL----PPPPQAR-TKLSYNC  108
BnaCZFP9       SPPR-SHAKSQSQDPSEEEYLALCLLMLAKDQPRTRFQPSL----PPPPQAR-TKLSYNC  108
BrZFP12        TPPR-LHAKSQSQDPSEEEYLALCLLMLAKDQPRTRFQPSP----PPPPQERTTKLSYNC  110
BnaAZFP10      TPPR-LHAKSQSQDPSEEEYLALCLLMLAKDQPRTRFQPSP----P-PPQERTTKLSYNC  109
BoZFP30        ------------HRLTEEEYLAFCLMLLARDGDRHHV--------------EEEKTVYKC  71
BnaCZFP27      ------------HRLTEEEYLAFCLMLLARDGDRHHV--------------EEEKTVYKC  71
BrZFP24        ------------HRLSEEEYLAFCLMLLARDGDRHHV--------------EEEKTVYKC  71
BnaAZFP24      ------------HRLTEEEYLAFCLMLLARDGDRHHV--------------EEEKTVYKC  71
BoZFP36        ------------RSLTEDEYIALCLMLLARDGNRTRDLPSCSS-LPPLLPTPTSTHTHKC  89
BnaCZFP34      ------------RSLTEDEYIALCLMLLARDGNRTRDLPSCSS-LPPLLPTPTSTHTHKC  89
BnaAZFP34      ------------RSLTEDEYIALCLMLLARDGNRTRHLPSSSS-SPPLLPTLTSTHLHKC  89
BoZFP29        -------H----HNLTEEEDLAFCLMLLARDGNRQL-LPLP----PVTVVAEKSSLRYKC  89
BnaCZFP26      -------H----HNLTEEEDLAFCLMLLARDGNRQL-LPLP----PVTVVAEKSSLRYKC  89
BrZFP30        -------H----KNLTEEEYLAFCLLLLARDGDRSNRNPLP----PPPVTVAEKSSTYTC  90
BoZFP25        -------H----KNLTEEEYLAFCLLLLARDGDRSNRNPLP----PPPVTVGEKSSTYTC  90
BnaCZFP22      -------H----KNLTEEEYLAFCLLLLARDGDRSNRNPLP----PPPVTVGEKSSTYTC  90
BnaAZFP30      -------H----KNLTEEEYLAFCLLLLARDGDRSNRNPLP----PPPVTVAEKSSTYTC  90
BoZFP16        -------Q----QNLTEEEYLAFCLLLLARDGNRSNRQPLP----LPPVAEKSSSSSYKC  90
BnaCZFP13      -------Q----QNLTEEEYLAFCLLLLARDGNRSNRQPLP----LPPVAEKSSSSSYKC  90
BrZFP28        -------Q----QNLTEEEYLAFCLLLLARDGNRSNRQPLP----PVTAAEKSSSSSYKC  90
BnaAZFP28      -------Q----QNLTEEEYLAFCLLLLARDGNRSNRQPLP----PVTAAEKSSSSSYKC  90
BoZFP14        -------HHNQETTPSEEEYLALCLLMLARGTAVQPPL----TPPPPSHRSRSDHRDFKC  97
BnaCZFP11      -------HHNQETTPSEEEYLALCLLMLARGTAVQPPL----TPPPPSHRSRSDHRDFKC  97
BrZFP23        -------HHNQETTPSEEEYLALCLLMLARGTAVQPPL----TPPPPSRRSPSDHRDFKC  97
BnaAZFP23      -------HHNQETTPSEEEYLALCLLMLARGTAVQPPP----TPPPPSRRLSSDHRDFKC  97
BoZFP9         -------RLDQETAPSEEEYLALCLLMLARGSAVKSPL-----RPSP----SSDHRGYKC  92
BnaCZFP6       -------RLDQETAPSEEEYLALCLLMLARGSAVKSPL-----RPSP----SSDHRGYKC  92
BrZFP7         -------RLNQETAPSEEEYLALCLLMLARGSAVQSPL-----PPSS----SSDHRGYKC  92
BnaAZFP38      -------RLNQETAPSEEEYLALCLLMLARGSAVQSPL-----PPSS----SSDHRGYKC  92
BrZFP29        ----------QEKTPSEEEYLALCLLMLARGSTVQSLP----PPSLP----SSDHRGYKC  90
BnaAZFP29      ----------QEKTPSEEEYLALCLLMLARGSTVQSLP----PPSLP----SSDHRGYKC  90
BoZFP33        ----------QEKTPSEEEYLALCLLMLARGSTVKSPP----PPSLP----SSDHRGYKC  90
BnaCZFP29      ----------QEKTPSEEEYLALCLLMLARGSTVKSPP----PPSLP----SSDHRGYKC  90
BrZFP1         ----------KSNPPSEEEYLALCLLMLARGSS--DD--DHHSSPP-PPPSDHHHRDYKC  87
BnaAZFP1       ----------KSNPPSEEEYLALCLLMLARGSS--DD--DHHSSPPPPPPSDHHHRDYKC  88
BoZFP3         ----------QSNPPSEEEYFALCLLMLARGSS--D---DDHHSSPPHPPSDHHHRDYKC  87
BnaCZFP2       ----------QSNPPSEEEYFALCLLMLARGSS--D---DDQHSSPPHPPSDHHHRDYKC  87
BnaCZFP3       ----------KSNPPSEEEYLALCLLMLARGSS--STNDGNDHPSPPAPPSDHHHRDYKC  90
BrZFP13        ----------QSHPPSEEEYLAICLLMLARGSSSSSSNDGNDHHSPPAPPSDHHHRDYKC  91
BnaAZFP11      ----------QSHPPSEEEYLAICLLMLARGSSSSSSNDGNDHHSPPAPPSDHHHRDYKC  91
BoZFP31        ----------QSHPPSEEEYLAICLLMLARGSS--STNDGNDHHSPPAPPSDHDHRDYKC  89
BnaCZFP28      ----------QSHPPSEEEYLAICLLMLARGSS--STNDGNDHPSPPAPPSDHHHRDYKC  89
                             :*:* :*:**: **.                       ..*
```

```
               ---------ZFP#1------
BoZFP26        SVCGKAFPSYQALGGHKASHRIKPPTTTADDD--------------STPPTIAVAHPTST 152
BnaCZFP23      SVCGKAFPSYQALGGHKASHRIKPPTTTADDD--------------STPPTIAVAHPTST 149
BrZFP20        SVCGKAFPSYQALGGHKASHRIKPQTTTADDD--------------STTPTIAVAHPTST 150
BnaAZFP18      SVCGKAFPSYQALGGHKASHRIKPPTTTADDD--------------STTPTIAVAHPTST 150
BoZFP5         SVCGKAFPSYQALGGHKASHRIKPLTAD--NS--------------TSPIIAGEKHHSSA 148
BnaCZFP4       SVCGKAFPSYQALGGHKASHRIKPLTAD--NS--------------TSPIIAGEKHHSSA 148
BrZFP2         SVCGKAFPSYQALGGHKASHRIKPLTAD--NS--------------TSPIIAGEKHHSSA 151
BnaAZFP2       SVCGKAFPSYQALGGHKASHRIKPLTAD--NS--------------TSPIIAGEKHHSSA 151
BoZFP13        SVCGKAFLSYQALGGHKASHRIKPPTAG--DS--------------TAPSIAGEKHPTST 152
BnaCZFP9       SVCGKAFLSYQALGGHKASHRIKPPTAG--DS--------------TAPSIAGEKHPTST 152
BrZFP12        SVCGKAFLSYQALGGHKASHRIKPPTAG--DS--------------TAPSIAGEKHPTST 154
BnaAZFP10      SVCGKAFVSYQALGGHKASHRIKPPTAG--DS--------------TAPSIAGEKHLTST 153
BoZFP30        GVCDKVFLSYQALGGHKASHRNFSS-----GGDVKP--------TT-------------- 104
BnaCZFP27      GVCDKVFLSYQALGGHKASHRNFSS-----GGDVKP--------TT-------------- 104
BrZFP24        GVCDKVFLSYQALGGHKASHRNLSS-----GGDVKP--------TT-------------- 104
BnaAZFP24      GVCDKVFLSYQALGGHKASHRNLSS-----GGDVKP--------TT-------------- 104
BoZFP36        SVCDKTFSSYQALGGHKASHRKNSSQTQSSRGDEKS--------TSSAITI----VRHG- 136
BnaCZFP34      SVCDKAFSSYQALGGHKASHRKKSSQTQSSGGDEKS--------TSSAITI----VRHG- 136
BnaAZFP34      SVCDKAFSSYQALGGHKASHRKNSSQTQSSGGDEKS--------TSSAITI----ASHGG 137
BoZFP29        SVCDKSFSSYQALGGHKASHRKNVSQTHSAGGDDQS--------TSSATTA----SAVT- 136
BnaCZFP26      SVCDKSFSSYQALGGHKASHRKNVSQTHSAGGDDQS--------TSSATTA----SAVT- 136
BrZFP30        SVCDKSFSSYQALGGHKASHRKNLSQTLSGGGDDQS--------TL---TT----SAAT- 134
BoZFP25        SVCDKSFSSYQALGGHKASHRKNLSQTLSGGGDDQS--------TS---TT----SAVT- 134
BnaCZFP22      SVCDKSFSSYQALGGHKASHRKNLSQTLSGGGDDQS--------TS---TT----SAVT- 134
BnaAZFP30      SVCDKSFSSYQALGGHKASHRKNLSQTLSGGGDDQS--------TS---TT----SAVT- 134
BoZFP16        SVCDKSFSSYQALGGHKASHRKNSSQSQSGGGDDQS--------TSA-TTT----SAVT- 136
BnaCZFP13      SVCDKSFSSYQALGGHKASHRKNSSQTQSGGGDDQS--------TSA-TTT----SAVT- 136
BrZFP28        SVCDKSFSSYQALGGHKASHRKNSSQTQSGGGDDQS--------TSA-TTT----SAVT- 136
BnaAZFP28      SVCDKSFSSYQALGGHKASHRKNSSQTQSGGGDDQS--------TSA-TTT----SAVT- 136
BoZFP14        TVCGKSFNSYQALGGHKTSHRKPPANNVNVPSSQEPSNNN-SHGNGGSVVFSGNGTASN- 155
BnaCZFP11      TVCGKSFNSYQALGGHKTSHRKPPANNVNVPSSQEPSNNN-SHGNGGSVVFSGNGTASN- 155
BrZFP23        TVCGKSFNSYQALGGHKTSHRKPPANNANVPSGQEPSNNK-SHSNGGSVLFSGNGTVSN- 155
BnaAZFP23      TVCGKSFNSYQALGGHKTSHRKPPANNANVPSGQEPSNNK-SHSNGGSVIFNGNGTVSN- 155
BoZFP9         TVCGKSFSSYQALGGHKTSHRKP-ASNVNVPINQEPSNNSHSNSNGGSVVINGNG----- 146
BnaCZFP6       TVCGKSFSSYQALGGHKTSHRKP-ASNVNVPINQEPSNNSHSNSNGGSVVINGNG----- 146
BrZFP7         TVCGKSFSSYQALGGHKTSHRKP-ASNVNVPINQEQSNNSHSNSNGGSVAINGNG----- 146
BnaAZFP38      TVCGKSFSSYQALGGHKTSHRKP-ASNVNVPINQEQSNNSHSNSNGGSVAINGNG----- 146
BrZFP29        TVCGKSFSSYQALGGHKTSHRKP-VNNTDVPSNQEPFNNTHRNSNGGSVVINGNG----- 144
BnaAZFP29      TVCGKSFSSYQALGGHKTSHRKP-VNNTDVPSNQEPFNNTHRNSNGGSVVINGNG----- 144
BoZFP33        TVCGKSFSSYQALGGHKTSHRKP-VNTINGPSAQEPSNKTHGNSNGGSIVINGNG----- 144
BnaCZFP29      TVCGKSFSSYQALGGHKTSHRKP-VNTNNGPSAQEPSNKTHGNSNGGSIVINGNG----- 144
BrZFP1         SVCGKSFPSYQALGGHKTSHRKPVSNN-N-NNHD-------DNNNSGNGSITNNGNISNG 138
BnaAZFP1       SVCGKSFPSYQALGGHKTSHRKPVSNN-N-YNHD-------DNNNSGNGSITNNGNISNG 139
BoZFP3         SVCGKSFPSYQALGGHKTSHRKPVSNN-NNNNHD-------GHNNSGNGSITNNGNISNG 139
BnaCZFP2       SVCGKSFPSYQALGGHKTSHRKPVSNN-NNNNHD-------GHNNSGNGSITNNGNISNG 139
BnaCZFP3       SVCGKSFPSYQALGGHKTSHRKPVSNN-NNNNHD-------GHNNSGNGSITNNGNISNG 142
BrZFP13        SVCGKSFPSYQALGGHKTSHRKPVSNT-N---CQ-------DTNNSGNGSVTNKGNISNG 140
BnaAZFP11      SVCGKSFPSYQALGGHKTSHRKPVSNT-N---CQ-------DTNNSANGSVTNNGNISNG 140
BoZFP31        SVCGKSFPSYQALGGHKTSHRKPVSNI-N---CQ-------DTINSGNGSVTNNGNISNG 138
BnaCZFP28      SVCGKSFPSYQALGGHKTSHRKPVSNT-N---CQ-------DTINTGNGSVTNNGNI--- 135
                *  * * *********:***
```

```
                       -----------ZF#2---------
BoZFP26        AIAPSGKIHECSICHKVFPTGQALGGHKRCHYEGTMGGG-GGSKSVSQSGSVTSTVS--- 208
BnaCZFP23      AIAPSGKIHECSICHKVFPTGQALGGHKRCHYEGTIGGG-GGSKSVSQSGSVTSTVS--- 205
BrZFP20        AIAPSGKIHECSICHKVFPTGQALGGHKRCHYEGTIGGG-GGSKSVSQSGSVTSTVS--- 206
BnaAZFP18      AIAPSGKIHKCSICHKVFPTGQALGGHKRCHYEGTIGGG-GGSKSVSQSGSVTSTVS--- 206
BoZFP5         TVPPSGKIHECSICRKVFPTGQALGGHKRCHYEGNLGG---GSKTISQSGSVSSTVS--- 202
BnaCZFP4       TVPPSGKIHECSICRKVFPTGQALGGHKRCHYEGNLGG---GSKTISQSGSVSSTVS--- 202
BrZFP2         TVPPSGKIHECSICRKVFPTGQALGGHKRCHYEGNLGG---GSKSISQSGSVSSTVS--- 205
BnaAZFP2       TVPPSGKIHECSICRKVFPTGQALGGHKRCHYEGNLGG---GSKSISQSGSVSSTVS--- 205
BoZFP13        TIAPSGKIHECSICHKVFPTGQALGGHKRCHYEGNLGGGGGGSKSVSHSGSVSSTVS--- 209
BnaCZFP9       TIAPSGKIHECSICHKVFPTGQALGGHKRCHYEGNLGGGGGGSKSVSHSGSVSSTVS--- 209
BrZFP12        TIAPSGKIHECSICHKVFPTGQALGGHKRCHYEGNLGG---GSKSVSHSGSVSSTVS--- 208
BnaAZFP10      AIAPSGKIHECSICHKVFPTGQALGGHKRCHYEGNLGGGGGGSKSVSHSGSVSSTVS--- 210
BoZFP30        --PAAVKSHVCSICHKSFTTGQALGGHKRCHYDGSSN-----VVSNSEGVGSTSHVSG-- 155
BnaCZFP27      --PAAVKSHVCSICHKSFTTGQALGGHKRCHYDGSSN-----VVSNSEGVGSTSHVSG-- 155
BrZFP24        --PSAVKSHVCSICHKSFATGQALGGHKRCHYDGSSS-----VVSNYEGVGSTSHV---- 153
BnaAZFP24      --PSAVKSHVCSICHKSFATGQALGGHKRCHYDGSSS-----VVSNSEGVGSTSHV---- 153
BoZFP36        --GGSVKPHVCSICNKSFATGQALGGHKRCHYEGKNGGGGSSSVSISEGVGSTSHVSS-- 192
BnaCZFP34      --GGSVKPHVCSICNKSFATGQALGGHKRCHYEGKNGGGGSSSVSISEGVGSTSHVSS-- 192
BnaAZFP34      GGGGSVKSHVCSICNKSFATGQALGGHKRCHYEGKNGS-------SSEGVGSTSHVSS-- 188
BoZFP29        --TGSGKSHVCSICQKSFPSGQALGGHKRCHYEGNNN---TSSVSNSEGAGSTSHVSS-- 189
BnaCZFP26      --TGSGKSHVCSICQKSFPSGQALGGHKRCHYEGNNN---TSSVSNSEGAGSTSHVSS-- 189
BrZFP30        --TGSGKSHVCSICHKSFPSGQALGGHKRCHYEGNNNS--SSSVANSEGAGSTSHVSS-- 188
BoZFP25        --TGSGKSHVCSICHKSFPSGQALGGHKRCHYEGNNNS--SSSVANSEGAGSTSHVSS-- 188
BnaCZFP22      --TGSGKSHVCSICHKSFPSGQALGGHKRCHYEGNNNS--SSSVANSEGAGSTSHVSS-- 188
BnaAZFP30      --TGSGKSHVCSICHKSFPSGQALGGHKRCHYEGNNNS--SSSVANSEGAGSTSHVSS-- 188
BoZFP16        --TGSGKSHVCTICHKSFPSGQALGGHKRCHYEGNNNN--TSSVSNSEGAGSTSHVSI-- 190
BnaCZFP13      --TGSGKSHVCTICNKSFPSGQALGGHKRCHYEGNNNN--TSSVSNSEGAGSTSHVSI-- 190
BrZFP28        --TGSGKSHVCTICHKSFPSGQALGGHKRCHYEGNNNN--TSSVSNSEGAGSTSHVSI-- 190
BnaAZFP28      --TGSGKSHVCTICHKSFPSGQALGGHKRCHYEGNNNN--TSSVSNSEGAGSTSHVSI-- 190
BoZFP14        GVNLSGKIHTCSICFKSFSSGQALGGHKRSHYDGGNNGNGNG----SVEVMGGSDVSDVD 211
BnaCZFP11      GVNLSGKIHTCSICFKSFSSGQALGGHKRSHYDGGNNGNGNG----SVEVMGGSDVSDVD 211
BrZFP23        GVNQSGKIHTCSICFKSFSSGQALGGHKRCHYDGGNNGNGNG----SVEVMGGSDVSDVD 211
BnaAZFP23      GVNQSGKIHTCSICFKSFSSGQALGGHKRCHYDGGNNGNGNG----SVEVMGGSDVSDVD 211
BoZFP9         -FSQSGKIHTCSICFKSFSSGQALGGHKRCHYDGGNNGNGNGSSSNSVEVVGGSDGSYVD 205
BnaCZFP6       -FSQSGKIHTCSICFKSFSSGQALGGHKRCHYDGGNNGNGNGSSSNSVEVVGGSDGSYVD 205
BrZFP7         -VSQSGKIHTCSICFKSFSSGQALGGHKRCHYDAGNNGNGNGSSSNSVEVVGGSDGSYVD 205
BnaAZFP38      -VSQSGKIHTCSICFKSFSSGQALGGHKRCHYDAGNNGNGNGSSSNSVEVVGGSDGSYVD 205
BrZFP29        -VSQSGKTHTCSICFKSFSSGQALGGHKRCHYDGGNNGN--GSSSNSVEVIGGSDVSDVD 201
BnaAZFP29      -VSQSGKTHTCSICFKSFSSGQALGGHKRCHYDGGNNGN--GSSSNSVEVIGGSDVSDVD 201
BoZFP33        -VSQSGKTHTCSICFKSFSSGQALGGHKRCHYDGGNNGN--GSSSNSVEVIGGSDVSDVD 201
BnaCZFP29      -VSQSGKTHTCSICFKSFSSGQALGGHKRCHYDGGNNGN--GSSSNSVEVIGGSDVSDVD 201
BrZFP1         LIGQSGKTHKCSICFKSFPSGQALGGHKRCHYDGGNSNSNIN------------------ 180
BnaAZFP1       LIGQSGKTHKCSICYKSFPSGQALGGHKRCHYDGGNSNSNIN------------------ 181
BoZFP3         LIGHSGKTHKCSICFKSFPSGQALGGHKRCHYDGGNGNSNIN------------------ 181
BnaCZFP2       LIGHSGKTHKCSICFKSFPSGQALGGHKRCHYDGGNGNSNIN------------------ 181
BnaCZFP3       LIGHSGKTHKCSICFKSFPSGQALGGHKRCHYDGGNGNSNIN------------------ 184
BrZFP13        LIGQSGKTHKCSICFKSFPSGQALGGHKRCHYDGGN------------------------ 176
BnaAZFP11      LISQSGKTHKCSICFKSFPSGQALGGHKRCHYDGGN------------------------ 176
BoZFP31        LIGQSGKTHKCSICFKSFPSGQALGGHKRCHYDGGN------------------------ 174
BnaCZFP28      -------THKCSICFKSFPSGQALGGHKRCHYDGGN------------------------ 164
                       * *:** * * :*********.**:.
```

```
                              --EAR--
BoZFP26        EERS-------NRAFIDLNLPALPELSLHHHNPVVDEEIQSPLTGKKPLLLTDHDKASIK 261
BnaCZFP23      EERS-------NRAFIDLNLPALPELSLHHHNPVVDEEIQSPLTGKKPLLLTDHDKASIK 258
BrZFP20        EERS-------NRVFIDLNLPALPELSLHHHNTVVDEEIQSPLTGKKPLLSTDHDKVIIK 259
BnaAZFP18      EERS-------NRVFIDLNLPALPELSLHHHNTVVDEEIQSPLTGKKPLLSTDHDKVIIK 259
BoZFP5         EDRS-------NHVLIDLNLPALPELSLH-HNPVVDEEILSPLTGKKPLLLTDRDQV-IK 253
BnaCZFP4       EDRS-------NHVLIDLNLPALPELSLH-HNPVVDEEILSPLTGKKPLLLTDRDQV-IK 253
BrZFP2         EDRS-------NRVLIDLNLPALPELSLH-HNPVVDDEILSPLTGKKPLLLTDRDRV-IE 256
BnaAZFP2       EDRS-------NRVLIDLNLPALPELSLH-HNPVVDDEILSPLTGKKPLLLTDRDQV-IK 256
BoZFP13        EGRS-------NRVIIDLNLPALPELSLH-HDSVVDEEILSPLTGKKPLLLTGHDQV-IK 260
BnaCZFP9       EGRS-------NRVIIDLNLPALPELSLH-HDSVVDEEILSPLTGKKPLLLTGHDQV-IK 260
BrZFP12        EERS-------NRVIIDLNLPALPELSLH-HDPVVDDEILSPLTGKKPLLLTGRDQV-IK 259
BnaAZFP10      EERS-------NRVIIDLNLPALPELSLH-HDPVVDDEILSPLTGKKPLLLTGHDQV-IK 261
BoZFP30        ---N-------SSRGFDLNITPINELS-------PDDEVMSPLPSKKLR--LK------- 189
BnaCZFP27      ---N-------SRRGFDLNITPINELS-------PDDEVKSPLPSKKLR--LT------- 189
BrZFP24        ------------SRGLDLNITPINEFS-------PDDEVMSPLPSKKLR--LK------- 185
BnaAZFP24      ------------SRGFDLNITPVNEFS-------PDDEVLSPLPSKKLR--LK------- 185
BoZFP36        ---GSH-HHHHHHRGFDLNIPPIPEFSTVN----GEEEVMSPMPAKKLR--LE------- 235
BnaCZFP34      ---GSHHHHHHHHRGFDLNIPPIPAFWTVN----GEEEVMSPMPTKKLR--LE------- 236
BnaAZFP34      ---GSH----HHHRGFDLNIPPIPEFSTVN----GEEEVMSPMPTKKMR--LE------- 228
BoZFP29        -----------SHRGFDLNIPPIPEFSTVN----GDDEVMSPMPAKKPR--LDFLEKLN- 231
BnaCZFP26      -----------SHRGFDLNIPPIPEFSTVN----GDDEVMSPMPAKKPR--LDFLEKLN- 231
BrZFP30        -----------GHRGFDLNIPPVPEFSLVN----GDDEVMSPMPAKKPR--FDFSEKA-- 229
BoZFP25        -----------GHRGFDLNIPPVPEFSLVN----GDDEVMSPMPAKKPR--FDFSEKA-- 229
BnaCZFP22      -----------GHRGFDLNIPPVPEFSLVN----GDDEVMSPMPAKKPR--FDFSEKA-- 229
BnaAZFP30      -----------GHRGFDLNIPPVPEFSLVN----GDDEVMSPMPAKKPR--FDFSEKA-- 229
BoZFP16        -----------SHRGFDLNIPPIPEFSLVN----GDDEVMSPMPAKKPR--FDFSEKLQH 233
BnaCZFP13      -----------SHRGFDLNIPPIPEFSLVN----GDDEVMSPMPAKKPR--FDFSEKLQH 233
BrZFP28        -----------SHRGFDLNIPPIPEFSLVN----GDDEVMSPMPAKKPR--FDFSEKLQH 233
BnaAZFP28      -----------SHRGFDLNIPPIPEFSLVN----GDDEVMSPMPAKKPR--FDFSEKLQH 233
BoZFP14        DERSSEQTAFGGHRGFDLNLPADQVSVVIA------------------------------ 241
BnaCZFP11      DERSSEQTAFGGHRGFDLNLPADQVSVVIA------------------------------ 241
BrZFP23        DERSSEQSAIGGHRGFDLNLPADQVSVAIS------------------------------ 241
BnaAZFP23      DERSSEQSAIGGHRGFDLNLPADQVSVVIS------------------------------ 241
BoZFP9         DERSSEQSATGDNRGFDLNLPADQVAVVIS------------------------------ 235
BnaCZFP6       DERSSEQSATGDNRGFDLNLPADQVAVVIS------------------------------ 235
BrZFP7         DERSSEQSATGDNRGFDLNLPADQVAVVIS------------------------------ 235
BnaAZFP38      DERSSEQSATGDNRGFDLNLPADQVAVVIS------------------------------ 235
BrZFP29        DERSSEQSGIGGHRGFDLNLPADQVTVVIS------------------------------ 231
BnaAZFP29      DERSSEQSGIGGHRGFDLNLPADQVTVVIS------------------------------ 231
BoZFP33        DERSSEQSAIGGHRGFDLNLPADQVTVVIS------------------------------ 231
BnaCZFP29      DERSSEQSAIGGHRGFDLNLPADQVTVVIS------------------------------ 231
BrZFP1         -------GNGSNSHGFDLNLPADQVFEVSC----DETLGKSQLSGEETKSPL-------- 221
BnaAZFP1       -------GNGSNSHGFDLNLPADQVFEVSC----DETLGKSQLSSEETKSPL-------- 222
BoZFP3         -------GNGSNNHGFDLNLPADQVFEVSC----DDTLGKSQLSGEETKSPL-------- 222
BnaCZFP2       -------GNGSNNHGFDLNLPADQVFEVSC----DDTLGKSQLSGEETKSPL-------- 222
BnaCZFP3       -------GNGSNNHGFDLNLPADQVFEVSC----DDTLGKSQLSGEETKSPL-------- 225
BrZFP13        -------GNGSNNHGFDLNLPADHD----------ETLVKSQLSGEELKSESDY------ 213
BnaAZFP11      -------GNGSNNHGFDLNLPADHD----------ETLVKC------------------- 200
BoZFP31        -------GNGSNNHGFDLNLPADHD----------ETLVRVNSPVKNQSRCHDYY----- 212
BnaCZFP28      -------GNGSNNHGFDLNLPADHD----------ETLVRVNSPVKNQSRCRDYY----- 202
                              :***:
```

BoZFP26	KEDFSLRI	269
BnaCZFP23	KEDFSLRI	266
BrZFP20	KEDFSLRI	267
BnaAZFP18	KEDFSLRI	267
BoZFP5	KEDLSLRI	261
BnaCZFP4	KEDLSLRI	261
BrZFP2	KEDLSLRI	264
BnaAZFP2	KEDLSLRI	264
BoZFP13	KEDLSLRI	268
BnaCZFP9	KEDLSLRI	268
BrZFP12	KEDLSLRI	267
BnaAZFP10	KEDLSLRI	269
BoZFP30	--------	189
BnaCZFP27	--------	189
BrZFP24	--------	185
BnaAZFP24	--------	185
BoZFP36	--------	235
BnaCZFP34	--------	236
BnaAZFP34	--------	228
BoZFP29	--------	231
BnaCZFP26	--------	231
BrZFP30	--------	229
BoZFP25	--------	229
BnaCZFP22	--------	229
BnaAZFP30	--------	229
BoZFP16	--------	233
BnaCZFP13	--------	233
BrZFP28	--------	233
BnaAZFP28	--------	233
BoZFP14	--------	241
BnaCZFP11	--------	241
BrZFP23	--------	241
BnaAZFP23	--------	241
BoZFP9	--------	235
BnaCZFP6	--------	235
BrZFP7	--------	235
BnaAZFP38	--------	235
BrZFP29	--------	231
BnaAZFP29	--------	231
BoZFP33	--------	231
BnaCZFP29	--------	231
BrZFP1	--------	221
BnaAZFP1	--------	222
BoZFP3	--------	222
BnaCZFP2	--------	222
BnaCZFP3	--------	225
BrZFP13	--------	213
BnaAZFP11	--------	200
BoZFP31	--------	212
BnaCZFP28	--------	202

```
2i-X Brassica ZFPs                                        --L-box--------
BoZFP23        -MDSSYTDFVMFNSQGQHDEGNMSRLPWKRVR---------DEDEELANCLVLLSNSGNA 50
BnaCZFP20      -MDSSYTDFVMFNSQGQHDEGNMSRLPWKRVR---------DEDEELANCLVLLSNSGNA 50
BrZFP31        MQNSSCIDLVMFSSRGQHDEGNMSRPPWKRERSNHHLNLYPNEDEELANCLVLLSNSGNA 60
BoZFP32        MQNSSCIDLVMFSSRGQHDEGNMSRPPWKRERSNHHLNLYPNEDEELANCLVLLSNSGNA 60
BnaCZFP33      MQNSSCIDLVMFSSRGQHDEGNMSRPPWKRKRSNHHLNLYPNEDEELANCLVLLSNSGNA 60
BrZFP32        -MNLSCIDFVMFSSRGQHDEGNMSRPSWKRERSNNLINLSPNEDEELANCLVLLSNSGDH 59
BnaAZFP31      -MNLSCIDFVMFSSRGQHDEGNMSRPSWKRERSNNLINLSPNEDEELANCLVLLSNSGDH 59
BoZFP24        -MNLSCIDFVMFSSRGQHDEGNMSRPSWKRERSNHLINLSPNQDEELANCLVLLSNSGDH 59
BnaCZFP21      -MNLSCIDFVMFSSRGQHDEGNMSRPSWKRERSNHLINLSPNQDEELANCLVLLSNSGDH 59
                 : *  *:***.*:**********  *** *         ::***************:
                                         -----------ZF#1-----------
BoZFP23        YNN----NEHGRIKDKNVKKRKTGHVFQCKGCKKVFASHQALGGHRASHKKVKGCFASQD 106
BnaCZFP20      YNN----NEHGHIKDKNVKKRKTGHVFQCKGCKKVFASHQALGGHRASHKKVKGCFASQD 106
BrZFP31        YNN----NKHGQGKSKTVKKQKTAHVFQCKACKKVFASHQALGGHRASHKKVKGCFASQD 116
BoZFP32        YNN----NKHGHGKSKTVKKQKTAHVFQCKACKKVFASHQALGGHRASHKKVKGCFASQD 116
BnaCZFP33      YNN----NKHGHGKSKTVKKQKTAHVFQCKACKKVFASHQALGGHRASHKKVKGCFASQD 116
BrZFP32        YNSGGHHNKHGHGKGKSIKKQKTSQAFQCKACKKVFTSHQALGGHRESHKKVKGCFATQN 119
BnaAZFP31      YNSGGHHNKHGHGKGKTIKKQKTSQAFQCKACKKVFTSHQALGGHRASHKKVKGCFATQN 119
BoZFP24        YNSGGHHNKHGHGKGKTIKKQKTSQSFQCKACKKVFTSHQALGGHRASHKKVKGCFATQD 119
BnaCZFP21      YNSGGHHNKHGHGKGKTIKKQKTSQSFQCKACKKVFTSHQALGGHRASHKKVKGCFATQD 119
               **.    *:**: * *.:**:**.: ****.*****:********* **********:*:
                                                         --NLS------ZF#2---
BoZFP23        KKAEEEEEEEEYKEE----EEEDEEEEE-------EEEPHITTRKRSNAHECTICHRVFSS 155
BnaCZFP20      KKAEEEEEEEEYEEE----EEE-EEEEE-------EDKPHITTRKRSNAHECTICHRVFSS 154
BrZFP31        KEEEEEEDEYKEDDDEEEEDDDAEEEEEEDKPT--AAAHIIARKRSNAHECTVCHRVFSS 174
BoZFP32        KEEEEEEDEYKEDDDEEEEDEDAEEEEEEDKPT--AAAHIIARKRSNAHECTVCHRVFSS 174
BnaCZFP33      KEEEEEEDEYKEDDDEEEEDEDAEEEEEEDKPT--AAAHIIARKRSNAHECTVCHRVFSS 174
BrZFP32        KEEEE-----DEDEYKE-EDEEEEEEEEEDKATAADYNNIITRKRSNAHECTICHRVFSS 173
BnaAZFP31      KEEEE-----DEDEYKE-EDEEEEEEEEEDKATAADYNNIITRKRSNAHECTICHRVFSS 173
BoZFP24        KEEEEEDEDEDEDEYKE-EDDEEEEEEEEDKATAADHNNIITRKRSNAHECTICHRVFSS 178
BnaCZFP21      KEEDEDE-------DED-EDDEEEEEEEEDKATAADHNNIITRKRSNAHECTIYHRVFSS 171
               *: :*             *::  ****           .* :**********: ******
               --------------                                       --EAR--
BoZFP23        GQALGGHK----RCHWLTPSTNYLRMKPLNDSSTHHHHSQPLDQPSLDLSLAC----VDP 207
BnaCZFP20      GQALGGHK----RCHWLTPSTNYLRMKPLNDSSTHHHHSQPLDQPSLDLSLAC----VDP 206
BrZFP31        GQALGGHK----RCHWLTPST-YLRMTPLHDSST--VRSQALDQPSLDLNLACQEYSLDP 227
BoZFP32        GQALGGHK----RCHWLTPST-YLRMTPLHDSSA--VRSQALDQPSLDLNLACQEYSVDP 227
BnaCZFP33      GQALGGHK----RCHWLTPST-YLRMTPLHDSSA--VRSQALDQPSLDLNLACQEYSVDP 227
BrZFP32        GQALGGHK----RCHWLTPSS-YFHMTPLHDSSSV-ARSQMLEQPSLDLNLTCQEYSVDP 227
BnaAZFP31      GQALGGHK----RCHWLTPSS-YFHMTPLHDSSSV-ARSQMLEQPSLDLNLTCQEYSVDP 227
BoZFP24        GQALGGHK----RCHWLTPST-YFLMTPLHDSSFA-ARSQMLEQPSLDLNLACQEYSVDP 232
BnaCZFP21      GQALGGHKRCHKRCHWLTPST-YFLMKPLHDSSFA-ARSQMLEQPSLDLNLACQEYSVDP 229
               ********    ********: *: *.**.***    :** *:******.*:*    :**

BoZFP23        TVMTIGRDGGGNNHNATTSSNSWLKLASGDWS 239
BnaCZFP20      TVMTIGRDGGGNNHNATTSSNSWLKLASGDWS 238
BrZFP31        TVMSVGRDGGGNIHNAT-SSNSWLKLASGDWS 258
BoZFP32        TTMSVGRDGGGNIHNAT-SSNSWLKLASGDWS 258
BnaCZFP33      TTMSVGRDGGGNIHNAT-SSNSWLKLASGDWS 258
BrZFP32        TVMSVWRDDGGNNHNAT-SPDSWLKLASGDWS 258
BnaAZFP31      TVMSVWRDDGGNNHNAT-SPDSWLKLASGDWS 258
BoZFP24        TVMRVGSDDGGNNHNAT-APDSWLKLARGDWS 263
BnaCZFP21      TVMRVGSDDGGNNHNAT-APDSWLKLARGDWS 260
               *.* :  * *** **** : :****** ****
```
