# Supplementary material for: Comparative analysis of the genetic variability within the Q-type C2H2 zinc-finger transcription factors in the economically important cabbage, canola and Chinese cabbage genomes
Source: Hereditas. 2018 Sep 21;155:29. doi: 10.1186/s41065-018-0065-5 (PMC6150991; doi:10.1186/s41065-018-0065-5)

Additional File 3

Title: Comparative analysis of the genetic variability within the Q-type C2H2 zinc-finger transcription factors in the economically important cabbage, canola and Chinese cabbage genomes

Additional File 3

A phylogenetic tree of 146 *Brassica oleracea*, *rapa* and *napus* ZFPs and 18 Arabidopsis ZFPs form 5 groups. The proteins were aligned with CLUSTALW and the phylogenetic tree was generated using the maximum likelihood method with 1000 bootstrap replicates.

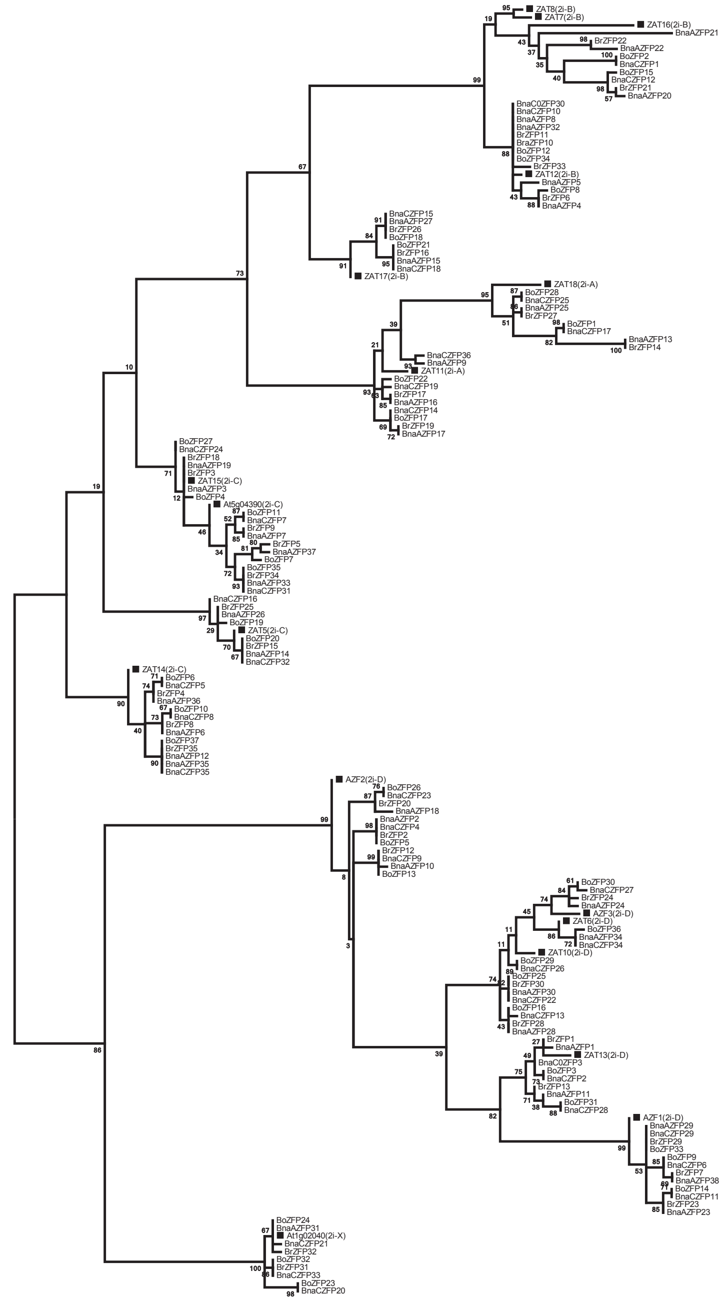

Supplement: Supplementary file 3 — Complete phylogenetic tree of 146 Brassica ZFPs and 18 Arabidopsis ZFPs form 5 groups. (PDF 692 kb) [file 41065_2018_65_MOESM3_ESM.pdf]
