# Supplementary material for: Comparative analysis of the genetic variability within the Q-type C2H2 zinc-finger transcription factors in the economically important cabbage, canola and Chinese cabbage genomes
Source: Hereditas. 2018 Sep 21;155:29. doi: 10.1186/s41065-018-0065-5 (PMC6150991; doi:10.1186/s41065-018-0065-5)
Supplement: Supplementary file 4 — SRA data confirming expression of B. napus ZFPs (PDF 195 kb) [file 41065_2018_65_MOESM4_ESM.pdf]

Additional File 4: SRA data confirming expression of *B. napus* ZFPs
Title; Comparative analysis of the genetic variability within the Q-type C2H2 zinc-finger transcription factors in the economically important cabbage, canola and Chinese cabbage genomes
.

Additional File 4: *B. napus* transcript data encompasses the coding region of the protein only. The transcripts are divided by groups, 2i-A-D, and subgroups by their homology to 17 Arabidopsis ZFPs. Only the *B. napus* transcripts are aligned.
Grey highlights represent SRA coverage and consensus between transcripts. Red nucleotides represent differences within the alignment verified by SRA data. To assess whether the SRA data confirms a unique transcript, comparisons are made of the number of nucleotide differences between the specific BnaAZFP and BnaCZFP homologs or between tandem duplications within the same subgenome. Comparison of transcripts that were not a direct homolog or tandem duplication, always have more divergent nucleotides. Sequences without grey highlights could not be verified by SRA data.
Table 1 provides details on the source tissue and amount of sequence coverage of the SRA data.

2i-A transcripts p2-4
2i-B transcripts p5-9
2i-C transcripts p10-17
2i-D transcripts p18-27
Table 1 p28-30

2i-A

Zat11-like

```
BnaA04g21410D      ATGATGAAGAGAGAACGATCTGAGTTCGAAGAGTCCATCAAGAAT---CTAGACATTTCT    57
BnaC04g45160D      ---ATGAAGAGAGAACGATCTGAGTTCGAAGAGTCCATCAAGAAT---CTAGACATTGCT    54
BnaA05g07070D      ---ATGAAGAGAGAAAGATCTGAGTTCGAAGAATCCATCAAGATGTCTGATGACATTGCT    57
BnaC04g07880D      ---ATGAAGAGAGAAAGATCTGAGTTCGAAGAATCCATCAAGATGTCTGATGACATTGCT    57
BnaA03g17210D      ---ATGAAGAGAGAAAGATCTGAGTACGAAGAATCCATCAAGAAT---CTAGACATCGTT    54
BnaCnng48130D      ---ATGAAGAGAGAAAGATCTGAGTACGAAGAATCCATCAAGAAT---CTAGACATCGTT    54
                      ************ ********* ****** **********        *****   *

BnaA04g21410D      AAATGTCTAATGATACTCTCTCAAACCTCCTCCATGGTCAAACAGATTGGTGTGAA----    113
BnaC04g45160D      AAATGTCTAATGATACTATCTCAAACCTCCTCCATGGTCAAACAGATTGGCGTGAA----    110
BnaA05g07070D      AGATGTCTGATGATATTATCACAGACC---TCCATGGTCAAACAAGTCGATGTGAA----    110
BnaC04g07880D      AGATGTCTGATGATATTATCACAGACC---TCCATGGTCAAACAAGTCGATGTGAA----    110
BnaA03g17210D      AAATGTTTAATGATATTATCACGAACC---TCCATTGCTAAACAGATTGGTGTGATTCAC    111
BnaCnng48130D      AAATGTTTAATGATATTATCACGAACC---TCCATTGTTAAACAGATTGGTGTGA-----    106
                   * **** * ****** * ** *  ***   ***** *  *****  * *  ****

BnaA04g21410D      --TCAATATACCGAGACCAATACAAGTAACCGGTTCGAATGCAAAACGTGTAACAAGAGA    171
BnaC04g45160D      --TCAATATGCCGAGAGCAATTCAAGTAACCGGTTCGAATGCAAAACATGTAACAAGAGA    168
BnaA05g07070D      --CCAATATACCGAGCGCGATACAAGTAACCGGTTCGAATGCAAAACGTGTAACAAGAGA    168
BnaC04g07880D      --CCAATATACCGATCGCGATACAAGTAACCGGTTCGAATGCAAAACGTGTAACAAGAGA    168
BnaA03g17210D      AATCAGTTTACCGAGAGAAATACAAGCAATCGGTTCGAATGCAAAACATGTAACCGGAGA    171
BnaCnng48130D      -ACCAGTTTACCGAGAGAAATACAAGTAATCGGTTCGAATGCAAAACATGTAACCGGAGA    165
                      ** * * ****     ** **** ** ***************** ******  ****

BnaA04g21410D      TTCTCTTCGTTTCAAGCCCTTGGTGGCCACCGGGCTAGCCATAAGAAGCCAAAGCTGACC    231
BnaC04g45160D      TTCTCTTCGTTTCAAGCCCTTGGTGGCCACCGTGCAAGCCATAAGAAGCCAAAGCTAACC    228
BnaA05g07070D      TTCTCTTCGTTTCAAGCCCTAGGTGGCCACCGGGCAAGCCATAAGAAGCCAAAGCTATCC    228
BnaC04g07880D      TTCTCTTCGTTTCAAGCCCTAGGTGGCCACCGGGCAAGCCATAAGAAGCCAAAGCTATCC    228
BnaA03g17210D      TTTTCTTCGTTTCAAGCCCTTGGTGGCCACCGGGCAAGCCACAAGAAACCAAAGCTAGCC    231
BnaCnng48130D      TTTTCTTCGTTTCAAGCCCTTGGTGGCCACCGGGCAACCCACAAGAAACCAAAGCTAACC    225
                   ** ***************** *********** ** * *** ***** ********  **

BnaA04g21410D      GTTGACCAAAAGGTGGTGAAACAATATCTTACCAAAG------ACGGAACTCAAGCCCAT    285
BnaC04g45160D      GTTGACCAAAAGGTGGTGAAACAATATCTTACCAAAG------AAGGAACTCAAGCCCAT    282
BnaA05g07070D      GTCGACCAGAAAGAGGTGAAAC---ATGTTACCAACAATTATAATGGAACTCATATACAC    285
BnaC04g07880D      GTTGACCAGAAAGAGGTGAAAC---ATGTTACCAACAATTATAATGGAACTCATATGCAC    285
BnaA03g17210D      GTTGACGAGAAAGATGTGAAAC---ATCTTACAAAAAATTATAAAGGAACACATATGCAC    288
BnaCnng48130D      GTTGACGAGAAAGATGTGAAAC---ATTTTACTAAAAATTATAAAGGAACACATATGCAC    282
                   ** *** * ** *  *******   ** **** **        * ***** **    **

BnaA04g21410D      GAATGTACAATATGCGGTCAGAGTTTTGGGACCGGACAGGCTTTAGGCGGTCACATGAGA    345
BnaC04g45160D      GAGTGTACAATATGCGGTAAGAGTTTTGGGACCGGACAGGCTTTAGGCGGTCACATGAGA    342
BnaA05g07070D      GAGTGTTCGATATGCAGTCAGAGTTTTGGGACCGGACAGGCTTTAGGTGGTCACATGAGA    345
BnaC04g07880D      GAGTGTTCGATATGCGGTCAGAGTTTTGGGACCGGACAGGCTTTAGGTGGTCACATGAGA    345
BnaA03g17210D      AAATGTTCGAGATGCGATCAGAGTTTTGGGACCGGGCAGGCTTTAGGTGGTCATATGAGA    348
BnaCnng48130D      AAATGTTCGAGATGCGGTCAGAGTTTTGGGACCGGGCAGGCTTTAGGTGGTCATATGAGA    342
                    * *** * * ****  * **************** *********** ***** ******

BnaA04g21410D      CGGCATAGGTCAAGCATGACGGTGGAGCCATCGGAGCTCATCTCTCCTGTGATTCATAAC    405
BnaC04g45160D      CGGCATAGGTCAAGCATGATGGTGGAGCCATCGGAGCTCATCTCTCCTGTGATTCATAAC    402
BnaA05g07070D      CGGCATAGGTCAAGCGTGACAGTGGAGCCATTGCAGACCATCTCTCCCGTGAATTCTACC    405
BnaC04g07880D      CGACATAGGTCAAGCATGAAGGTGGAGCCATCGCAGCTCATCTCTCCCGTGATTTCTAAC    405
BnaA03g17210D      CGGCATAGGTCAAGCATGACGTTGGAGCCATCGCAGCGCATCTCTCCCCGGATTCCTACC    408
BnaCnng48130D      CGGCATAGGTCAAGCATGACGTTGGAGCCATCGCAGCGCATCTCTCCCGTGATTCCTACC    402
                   ** ************ ***   ********* * **  *********   ** *  ** *

BnaA04g21410D      ATGCCGGTTCTGAAACGATGTAGTAGTAGCAAGAGGGTTTTGTCTTTGGATTTGAATTTA    465
BnaC04g45160D      ATGCCGGTTCTGAAACGATGTAGTAGTAGCAAGAGGGTTTTGTCTTTGGATTTGAATTTA    462
BnaA05g07070D      GTACCGGTTCTGAAACGATGCAGTAGTAGCAAGAGGGTTTTGTCTTTGGATTTGAATCTA    465
BnaC04g07880D      GTACCGGTTCTGAAACGATGCAGTAGTAGCAAGAGGGTTTTGTCTTTGGATTTGAATCTA    465
BnaA03g17210D      ATGCCGGTCATGAAACGTTGCAGTAGTAGCAAGAGGGTTTTGTCTTTGGATCTGAATCTA    468
BnaCnng48130D      ATGCCGGTTATGAAACGTTGCAGTAGTAGCAAGAGGGTTTTGTCTTTGGATCTGAATCTA    462
                    * *****  ******* ** ****************************** ***** **
```

```
BnaA04g21410D      ACTCCCTTAGAGAATGATCTTGAAATTCTTTTTGGGAAGACGTTTTTCCCCAACATAGAT   525
BnaC04g45160D      ACTCCCTTAGAGAATGATCTTGAAATTCTTTTTGGGAAGACGTTTTTCCCAAACATAGAT   522
BnaA05g07070D      ACTCCCTTAGAGAATGATCTTGAAACTATTTTTGGGAAGACGTTTTTCCCGAACATAGAT   525
BnaC04g07880D      ACTCCCTTAGAGAATGATCTTGAAACTATTTTTGGGAAGACGTTTTTCCCGAACATAGAT   525
BnaA03g17210D      ACTCCCTTAGAAAATGATCTAGAAATTATTTTTGGGAAGAAGTTTTTCCCCAACATAGAT   528
BnaCnng48130D      ACTCCCTTAGAAAATGATCTTGAAATTATTTTTGGGAAAAAGTTTTTCCCCAACATAGAT   522
                   *********** ******** **** * ********** * ********* *********

BnaA04g21410D      ATGAAGTTTGTTGTTTAG 543
BnaC04g45160D      ATGAAGTTTGTTGTTTAG 540
BnaA05g07070D      ATGAAGTTCGTTGTTTAG 543
BnaC04g07880D      ATGAAGTTCGCTGTTTAG 543
BnaA03g17210D      ATGAAGTTCGTTGTTTAG 546
BnaCnng48130D      ATGAAGTTAGTTGTTTAG 540
                   ******** * *******
```

11 nucleotide difference between BnaA04g21410D and BnaC04g45160D

13 nucleotide difference between BnaA05g07070D and BnaC05g07880D

10 nucleotide difference and 1 insert between BnaA03g17210D and BnaCnng48130D

Zat18-like

```
BnaA07g37620D      ATGAAGAGAGACCGGTCCGACTACGAAGAATCCATCACAGATTTAGACATAGTAAAAAGT      60
BnaC06g14860D      ATGAAGAGAGACCGGTCCGATTACGAAGAATCCATCACAGATTTAGACATAGTAAAAAGT      60
BnaA04g04630D      ATGAAGAGAGACCGTTCAGATTACGAAGATTTCATCACGCATATAGACATGATAAAAACT      60
BnaC04g27120D      ATGAAGAGAGACCGTTCAGATTACGAAGATTTCATCACGCATATAGACATGGTAAAAACT      60
                   ************** ** ** ******** * ******  ** *******  ****** *

BnaA07g37620D      CTCATGATGTTATCTCAGAGTTTCGTGGTCAAGCAAATCGAGGTACAGCAATCAACCCGA      120
BnaC06g14860D      CTCATGATGTTATCTCAGAGTTTCGTGGTCAAGCAAATCGAGGTAAAGCAATCAACCCGA      120
BnaA04g04630D      CTAACGATGTCATCTCATAGTTTTGTGGTCAAGCAAATCGA---------ATCTACCGGA      111
BnaC04g27120D      ATAACGATGTTGTCTCATAGTTGTGTAGTCAAGCAAATCGA---------ATCTACCGGA      111
                    * * *****  ***** ****   ** **************         *** *** **

BnaA07g37620D      AGCAGAACCAATGATAACCAATTCGAGTGCAAAACCTGTAATCGAAAATTTGATTCGTTC      180
BnaC06g14860D      ACCAGAACCAATGATAACCAATTCGAGTGCAAAACCTGTAATCGAAAATTTGATTCGTTC      180
BnaA04g04630D      AGCAAAATCAACGGTAACCGCTTTGAATGCAAAACGTGTAACCGGAAATTTGATTCGTTC      171
BnaC04g27120D      AGCAAAACCAAAGGTAACCGCTTTGAATGCAAAACGTGTAACCGGAAATTTGATTCGTTC      171
                   * ** ** *** * *****   ** ** ******** ***** ** ***************

BnaA07g37620D      CAAGCTCTAGGAGGTCACAGAGCCAGCCACAAGAAACCTAAGCTG---AGTATTGACCAA      237
BnaC06g14860D      CAAGCTCTAGGAGGTCACAGAGCCAGCCACAAGAAACCTAAGCTG---AGTATTGACCAA      237
BnaA04g04630D      CAAGCTCTCGGAGGTCATAGAGCCAGCCACAAGAAACCTAAGCTGATGAGTGTTGAGCAA      231
BnaC04g27120D      CAAGCTCTCGGAGGTCATAGAGCCAGCCACAAGAAACCTAAGCTGGTGAGTGTTGAGCAA      231
                   ******** ******** ***************************    *** **** ***

BnaA07g37620D      GAACAAGTTAAGCATCGTTACAATGAGAATGCTGCGCATAAGTGTTCAATCTGCGGTCAA      297
BnaC06g14860D      GAACAAGTTAAGCATCGTAACAATGAGAATGCTGCGCATAAGTGTTCGACTTGCGGTCAA      297
BnaA04g04630D      GAACAAGCCAAGCATCGGAACAATGAGAATGATGTGCATAAGTGTTCAATCTGCAGTCAA      291
BnaC04g27120D      GAACAAGCCAAACAT--------------ACTGTGCATAAGTGTTCAATCTGCGGTCAA      276
                   *******  ** ***               ** ************ *  *** *****

BnaA07g37620D      ATGTTTGGGACAGGTCAAGCTTTAGGAGGTCACATGAGAAAGCACAGGGAGAGCATGAGA      357
BnaC06g14860D      ATGTTTGGGACAGGTCAAGCTTTAGGAGGTCACATGAGAAAGCACAGGGAGAGCATGAGA      357
BnaA04g04630D      ATGTTTGGGACCGGTCAAGCTCTAGGCGGTCACATTAGAAGGCACAGGGCGAGCATGATA      351
BnaC04g27120D      ATGTTTGGGACCGGTCAAGCTTTAGGTGGTCACATGAGAAAGCACAGGGAGAGCATGATA      336
                   *********** ********* **** ******** **** ******** ******** *

BnaA07g37620D      ACCGAACAACCGGTTGTCTCGTCTACGGTTTAT---------CCGGTTTTGAACCGATGT      408
BnaC06g14860D      ACCGAACAACCGGTCGACTCGTCTACGGTTTAT---------CCGGTTTTGAACCGATGT      408
BnaA04g04630D      AATGAACAATCGGTTATCTCTTCTAAGGTATAT---ACCAGTCCGGTTAATGATCAATGC      408
BnaC04g27120D      AATGAACAATCGGTTATCTCTTCTATGGTATGTAGTACCAGTCCGGTTATGA--------      388
                   *  ****** ****    *** **** *** * *         ******
```

```
BnaA07g37620D     AATACCAGCAAG---------TGCTTGGATTTGAATCTAACTCCATTGGAGAATGATCTT       459
BnaC06g14860D     AATAGCAGCAAG---------TGCTTGGATTTGAATCTAACTCCATTGGAGAATGACCTT       459
BnaA04g04630D     CAGAGCAGCAAGAGGGTTATGCGCTTGGACCTGAATCTAACTCCCTTGGAGAATGATCTT       468
BnaC04g27120D     -ATAGCAGCAAGAGGGTTATGCGCTTGGACCTGAATCTAACTCCATTGGAGAATGACCTT       447
                   * * *******          *******  ************* *********** ***

BnaA07g37620D     GTATATGTGTTTGGGAAGAATTTGGCT---------GATTTTAAGTTTGTAAACTAG 507
BnaC06g14860D     GTATATGCGTTTGGGAAGAATTTGGCT---------GATTTGAAGTTTGTAAATTAG 507
BnaA04g04630D     TTAAACATCTTTGCGAAGAATTTGGCTCCACATATTGATTTGAAGTTTGTAAACTAG 525
BnaC04g27120D     GTATATATATTTGCTAAGAATTTGGTTCCACATATTGATTTGAAGTTTGTAAACTAG 504
                   ** *    ****  ********** *         ***** *********** ***
```

Did not find a SRA experiment that contained BnaC04g27120D.
6 different nucleotides between BnaA07g37620D and BnaC06g14860D
4 inserts between BnaA07g37620D and BnaA04g04630D

2i-B

Zat17-like

```
BnaC04g15490D      ATGGAAAAGGAAAGAGAGATGGAGATGATCAACAAGATGGCAAGCTGCTTGATTTTTCTA    60
BnaA07g14080D      ATGGAAAAGGAAAGAGAGATGGAGATAATCAACAAGATGGCAAGCTGCTTGATTTTTCTA    60
BnaA04g16610D      ATGGAAAAGGAAAGAGAGATAGAGATGATCAACAAGATGGCAAGCTGCTTGATTTATCTA    60
BnaC04g39930D      ATGGAAAAGGAAAGAGAGATAGAGATGATCAACAAGATGGCAAGCTGCTTGATTTATCTA    60
                   ******************** ***** **************************** ****

BnaC04g15490D      TCAAAGGCTCACCAACACGACACCAAAGGCCGCGTTTTCGCGTGCAAGACATGCAACAAG    120
BnaA07g14080D      TCAAAGGCTCACCAACACGACACCAAAGGCCGCGTTTTCGCGTGCAAGACATGCAACAAG    120
BnaA04g16610D      TCAAAGGCTCACCAACACGACACCAAAAGCCGCGTTTTCGCGTGCAAGACATGCAACAAG    120
BnaC04g39930D      TCAAAGGCTCACCAACACGACACCAAAAGCCGCGTTTTCGCGTGCAAGACATGCAACAAG    120
                   *************************** ********************************

BnaC04g15490D      GAGTTCTCGTCGTTCCAAGCCTTGGGAGGCCACCGAGCAAGCCACCGCAGATCGGCCGCG    180
BnaA07g14080D      GAGTTCTCGTCGTTCCAAGCCTTGGGAGGCCACCGAGCAAGCCACCGCAGATCGGCCGCG    180
BnaA04g16610D      GAGTTCCCGTCGTTCCAAGCCTTGGGAGGCCACCGAGCAAGCCACCGCAGATCGGCTGCG    180
BnaC04g39930D      GAGTTCCCGTCGTTCCAAGCCTTGGGAGGCCACCGAGCAAGCCACCGCAGATCGGCTGCC    180
                   ****** ************************************************* ** 

BnaC04g15490D      CTTGAAGGCCACGCACCACCTTCTCCCAAAAGAGTCAAACCGGTGAAACACGAGTGTCCC    240
BnaA07g14080D      CTTGAAGGCCACGCACCACCTTCTCCCAAGAGAGTCAAACCGGTGAAACACGAGTGTCCC    240
BnaA04g16610D      CTTGAAGGCCACGCACCTCCTTCTCCCAAGAGAGTCAAACCGGTGAAACACGAGTGTCCT    240
BnaC04g39930D      CTTGAAGGCCATGCAACTCCTTCTCCCAAGAGAGTCAAACCGGTGAAACACGAGTGTCCT    240
                   *********** *** * ************ ***************************** 

BnaC04g15490D      ATATGTGGTGCCGAGTTCGCCGTAGGGCAGGCCCTGGGTGGGCACATGAGGAAGCATAGA    300
BnaA07g14080D      ATATGCGGTGCCGAGTTCGCCGTAGGGCAGGCCCTGGGTGGGCACATGAGGAAGCATAGA    300
BnaA04g16610D      ATATGCGGTGCCGAGTTCGCCGTAGGGCAAGCCTTGGGTGGTCACATGCGGAAGCATAGA    300
BnaC04g39930D      ATATGCGGTGCCGAGTTCGCCGTAGGGCAAGCCTTGGGTGGTCACATGCGGAAGCATAGA    300
                   ***** *********************** *** ******* ****** ***********

BnaC04g15490D      GGAGGAGGAGCTAGCCGGAGTTTAGCGCCAGCGCCGGTGACGATGAAAAAAACTGGCGGC    360
BnaA07g14080D      GGAGGAGGATCTAGCCGGAGTTTAGCGCCAGCGCCGGTGACGATGAAAAAAACTGGCGGC    360
BnaA04g16610D      GGAGGAGGAGGTAGCCGAAGCTTGGCACCAGCGCCGGTGACTATGAAGAAATCCGGCGGC    360
BnaC04g39930D      GGAGGAGGAGGTAGCCGAAGCTTGGCACCAGCGCCGGTGACTATGAAGAAATCCGGCGGC    360
                   *********  ****** ** ** ** ************** ***** *** * ******

BnaC04g15490D      GGTAACGCAAAGAGGGTTTTGTGTTTGGATTTGAACTTAACGCCGGTAGAGAATGAAGAT    420
BnaA07g14080D      GGTAACGCAAAGAGGGTTTTGTGTTTGGATTTGAACTTAACGCCGGTAGAGAATGAAGAT    420
BnaA04g16610D      GGTAATGGGAAAAGGGTCTTGTGTATGGATTTGAACTTGACGCCGGGAGAGAATGATGAT    420
BnaC04g39930D      GGTAATGGAAAGAGGGTCTTATGTATGGATTTGAACTTGACGCCGGGAGAGAATGATGAT    420
                   ***** *  ** ***** ** *** ************* ******* ********* ***

BnaC04g15490D      TTGAAGCTGGAGCTTGGGAGGTTGATTTTCTAA    453
BnaA07g14080D      TTGAAGCTGGAGCTTGGGAGGTTAATTTTATAA    453
BnaA04g16610D      TTGAAGTTGGAGCTTGGGATGTTGTGA------    447
BnaC04g39930D      TTGAAGCTGGAGCTTGGGAGGTTGTGA------    447
                   ****** ************ ***
```

4 nucleotide differences between BnaC04g15490D and BnaA07g1480D

7 nucleotide differences between BnaA04g16610D and BnaC04g39930D

Zat12-like

```
BnaA10g12780D    ATGGTTGCTATTTCAGAGATCAAGTCGACGGTGGAGACTACGG---CGGCGAACTGTCTG    57
BnaC09g35160D    ATGGTTGCTATTTCAGAGATTAAGTTGACGGTGGAAACAACGGCGGCGGCGAACTGTCTG    60
BnaC03g11570D    ATGGTTGCAATCTCTGAGATTAACCCGACGGTGGAGGCTACGG---CAGCGAACTGTCTG    57
BnaA03g09250D    ATGGTTGCAATCTCTGAGATTAACCCGACGGTGGAGGCTAAGG---CGGCGAACTGTCTG    57
BnaA02g06790D    ATGGTTGCGGTTTCTGAGAATAAGTCGACGGTGGAGGCTACGG---CAGCTAACTGTCTG    57
BnaA02g06780D    ATG------GTTGCGGAGATTAAGTCTACGGTGGAGGCTACGG---CGGCTAACTGTCTG    51
                 ***       *  * ****  **    ********  * * **   * ** *********

BnaA10g12780D    ATGCTCTTATCAAGGGTCGGACAAGAAAACGTTGACGGTGGAAGCGCAAAACGCGTTTTC    117
BnaC09g35160D    ATGCTTTTATCAAGAGTCGGACAAGAAAACGTGGACGGTGGAAGTGCAAAACGCGTTTTC    120
BnaC03g11570D    ATGCTTTTATCAAGAGTCGGACAAAA---AGGTGCGGA------TCAGAAACGCGTTTTC    108
BnaA03g09250D    ATGCTTTTATCAAGAGTCGGACAAAA---AGGTGGGGA------TCAAAAACGCGTTTTC    108
BnaA02g06790D    ATGCTTTTATCAAGAGTCGGACAAGAAAACGGTGGAGAT---TATAAAAAACGCGTTTTC    114
BnaA02g06780D    ATGCTTTTATCAAGAGTCAGACAAGAAAACGGTGGAGA------TACAAAACGCGTGTTC    105
                 ***** ******** *** ***** *    *  *  *           ******** ***

BnaA10g12780D    ACATGTAAGACGTGTTTGAAGGAGTTTCATTCGTTTCAAGCGTTGGGAGGTCACCGTGCG    177
BnaC09g35160D    ACATGTAAGACGTGTTTGAAGGAGTTTCATTCGTTTCAAGCGTTGGGAGGTCACCGAGCG    180
BnaC03g11570D    ACATGTAAAACGTGTTTGAAAGAGTTTCATTCGTTTCAAGCGTTGGGAGGTCACCGTGCG    168
BnaA03g09250D    ACATGTAAAACGTGTTTGAAAGAGTTTCATTCGTTTCAAGCGTTGGGAGGTCACCGTGCG    168
BnaA02g06790D    ACATGTAAGACGTGTTTAAAGGAGTTTCAGTCGTTTCAAGCGTTGGGAGGTCACCGTGCA    174
BnaA02g06780D    ACATGTAAAACGTGTCTGAAGGAGTTTCATTCGTTTCAAGCGTTGGGAGGTCACCGTGCA    165
                 ******** ****** * ** ******** ************************** **

BnaA10g12780D    AGCCACAAGAAGCCTAAC---AATGAGAATCTCTCTAGTTTGATGAAGAAGACGAAAGCT    234
BnaC09g35160D    AGCCACAAGAAGCCTAAC---AATGAGAGTCTCTCCGGTTTGATAAAGAAGTCCAAAGCT    237
BnaC03g11570D    AGCCACAAGAAACCTAAC---AATGAGAGTCTCTCTGGATTGGTGAAGAAAGCCAAAGCT    225
BnaA03g09250D    AGTCACAAGAAACCTAACAACAATGAGAGTCTCTCTGGATTGGTGAAGAAAGCCAAAGCT    228
BnaA02g06790D    AGTCACAATAAGCCTAAC---AACGAGAATCTGTCTGGTCTGATAAAGAAGGCGAAAACG    231
BnaA02g06780D    AGTCACAAGAAGCCTAAC---AACGAGAATCTGTCTGGTCTGATAAAGAAAGCGAAAACG    222
                 ** ***** ** ******   ** **** *** **  *  ** * *****  * *** *

BnaA10g12780D    TCGTCTTCCCATCCTTGTCCGATATGTGGAGTGGAGTTTCCCATGGGACAAGCTCTAGGC    294
BnaC09g35160D    GCGTCGTCGCATCCTTGTCCGATATGCGGAGTGGAGTTTCCCATGGGACAAGCTCTAGGA    297
BnaC03g11570D    CCTTCGTCGCATCCTTGTCCGATATGCGGAGTGGAGTTTCCGATGGGACAAGCTCTAGGA    285
BnaA03g09250D    CCTTCGTCGCACCCTTGTCCGATATGCGGAGTGGAGTTTCCGATGGGACAAGCTCTAGGA    288
BnaA02g06790D    CCGTCGTCTCATCCTTGTCCTATATGCGGAGTGGAGTTTCCGATGGGACAAGCTCTTGGA    291
BnaA02g06780D    CCGTCGTCTCATCCTTGTCCCATATGCGGAGTGGAGTTTCCGATGGGACAAGCACTTGGA    282
                  * ** ** ** ******** ***** ************** *********** ** **

BnaA10g12780D    GGACACATGAGGAGACACAGGAACGAGAGTGGCGGCGCCGGCGCGTTGGTTACACGCGAG    354
BnaC09g35160D    GGACACATGAGGAGACACAGGAACGAGAGTGGCGGCGCCGGCGCATTGGTTACACGCGAG    357
BnaC03g11570D    GGTCACATGAGGAGACACAGGAACGAAAGTGGCGGCGGCGCAGCGTTGGTTACACGGGCG    345
BnaA03g09250D    GGTCACATGAGGAGACACAGGAACGAGATTGGCGGCGGAGCAGCGTTGGTTACACGGGCG    348
BnaA02g06790D    GGACACATGAGGAAACATAGGAACGAGAATGGCGGCGGCGTCACGTTGGTTACAAGAGCG    351
BnaA02g06780D    GGACACATGAGGAAACATAGGAACGAGAATGGCGGCGGCGTCGCGTTGGTTACAAGAGCG    342
                 ** ********** *** ******** * ********  *   * ********* * * *

BnaA10g12780D    TTACTGTCGGAGGCGGCGTTGACGACGTTGAAGAAATCAAGCAGTGGGA---GGTTGGCT    411
BnaC09g35160D    TTACTGTCGGAGGCGGCGTTGACGACGTTGAAGAAATCAAGCAGTGGGA---GGTTGGCT    414
BnaC03g11570D    TTATTGCCGGAGCCGACGATGACGACGTTGAAGAAGTCGAGCAGTGGGAAGAGGGTGGCG    405
BnaA03g09250D    TTATTGCCGGAGCCGACGATGACGACGTTGAAGAAGTCGAGCAGTGGGAAGAGGGTGGCG    408
BnaA02g06790D    TTATTGCCGGAGCCGACGATGACGACGTTGAAGAAGTCGAGTAGTGGGAAGAGGGTGGCT    411
BnaA02g06780D    TTATTGCCGGAGCCGACGGTGACGACGTTGAAGAAGACGAGTAGTGGGAAGAGGGTGGCT    402
                 *** ** ***** ** ** ****************  * ** *******   ** ****

BnaA10g12780D    TGTCTGGATCTGAGTTTGGGAATGGTGGAGAATTTGAATCTCAAGTTGGAACTTGGAAGA    471
BnaC09g35160D    TGTCTGGATCTGAGTTTGGGGATGGTGGAGAATTTGAATCTCAAGTTGGAGCTTGGAAGA    474
BnaC03g11570D    TGTTTGGATCTGAGTCTGGGGATGGTGGAGAATTTGAATCTCAAGTTGGAGCTTGGAAGA    465
BnaA03g09250D    TGTTTGGATCTGAGTTTGGGGATGGTTGAGAATTTGAATCTCAAGTTGGAGCTTGGAAGA    468
BnaA02g06790D    TGTCTTGATCTGAGCTTGGGGATGGTTGAGAATTTGAATCTCAAGTTGGAGCTTGGAAGA    471
BnaA02g06780D    TGTCTTGATCTGAGCTTGGGGATGGTTGAGAATTTGAATCTCAAGTTGGAGCTTGGAAGA    462
                 *** * ********  **** ***** *********************** *********
```

```
BnaA10g12780D      CCTGTTTGTTGA      483
BnaC09g35160D      CCTGTTTGTTGA      486
BnaC03g11570D      ACAGTTTGTTAA      477
BnaA03g09250D      ACTGTTTGTTGA      480
BnaA02g06790D      ACAGTTTATTGA      483
BnaA02g06780D      ACAGTTTATTGA      474
                    * **** ** *
```

20 nucleotide differences between BnaA10g12780D and BnaC09g35160D
9 nucleotide differences and 1 three base insert between BnaC03g11570D and BnaA03g09250D
18 nucleotides differences and 1 two base insert between BnaA02g06790D and BnaA02g06780D

Zat16-like

```
BnaA06g19880D       ------------------------------------------------------------      0
BnaA06g18210D       ATGGTTGCAAGAAGT------------------------------------------GAG      18
BnaC03g55590D       ATGGTTGCAAGAAGTGAGAATCTTAATCGGACGGTGGAAAATAGAGCGCAGGATAGCGAG      60
BnaA06g19890D       ATGGTTGTA---------------------------------------------------      9
BnaC01g24440D       ATGGTTGCGAGAAGTGAGAA----------------------------------------      20

BnaA06g19880D       ------------------------ATGGTTTCAAGAAGTGAGAGTTTTGACCGGACGGTG      36
BnaA06g18210D       AAACTTGATCGGACTGTGGAAGGTACGGCGGGGAATAACGAGAAACTTGAGAGGACTGTG      78
BnaC03g55590D       AAACTTGATCGGACTGTGGAAGGTACGGCAGCGAATAACGAGAAACTTGAGAGGACGGTG      120
BnaA06g19890D       ---------------------------------AGAAGTGAGAATCTTGATCAGACAGTG      36
BnaC01g24440D       --------TCGGAGTGTGAAAGATACGCCAGCGGAAAATGAGAATTTTGATCGGACCGTG      72
                                                    *  ****   ****   *** ***

BnaA06g19880D       GAAGATACGACAACCAATTGTCTGATGCTGTTGTCGAGAGTTGGGGAATGCGGCGGAGGA      96
BnaA06g18210D       GAAGATAAGGCGGCGAACTGTCTGATGCTGTTATCAAGAATTGGGGAAAACGGCGGAGGA      138
BnaC03g55590D       GAAGATAAGGCGGCAAACTGTCTGATGCTGTTATCAAGAATTGGGGAAAACGGCGGAGGA      180
BnaA06g19890D       GAAGACACGGAGGCTACCTGTTTGATGCTGTTATCGAAAGTAGGTGAATGCGGCCGAGAA      96
BnaC01g24440D       GAAGATACGGCGACGAGTTGTCTGATGCTGTTACCTAGGGTAGGGAAATGCATCCGA---      129
                    ***** * *    * *  *** **********  * *   * **  **  *  * **

BnaA06g19880D       GACGTAGAGGGTCGCGTTTTCAGATGCAAGACTTGTATGAAAGAGTTCTCATCGTTCCAC      156
BnaA06g18210D       GGCCGAGAGAGTCGTGTTTACAGATGCAAAACGTGTATGAAAGAGTTCTCATCGTTCCAA      198
BnaC03g55590D       GGCCGAGAGAGTCGTGTTTACAGATGCAAAACGTGTATGAAAGAGTTCTCATCGTTCCAA      240
BnaA06g19890D       AGAGGAGAGACTCGCGTTTTCAGATGCAAGACTTGTACGAAAGAGTTCTCATCGTTCCAA      156
BnaC01g24440D       ---GGAGAGAGTCGCATTTACCGATGCAAGACATGTATGAAAGAGTTCTCATCGTTCCAA      186
                         ****  ***  *** * ******* ** **** *********************

BnaA06g19880D       GCTCTGGGAGTTCATCGCGTAAGCCACAAGAAGCCAGTTAGCAATAGCAACGGCGAAGAA      216
BnaA06g18210D       TCTTTAGGAGGCCATCGCGCAAGCCACAATAAACACGTTAACAACAGCAGCAGCGACGAA      258
BnaC03g55590D       TCTTTAGGAGGCCATCGCGCAAGCCACAATAAACACGTTAACAACAACAGCAGCGACGAA      300
BnaA06g19890D       GCTTTAGGAGGCCATCGGGCAAGCCACAAGAAGCCAGTTAACAACAGCAGTGACGAACAG      216
BnaC01g24440D       GCGTTGGGAGGCCATCGCGCAAGCCACAAGAAGCCAATTAGCAACTCCAGCGACGACTGT      246
                     *  * ****  ***** * ********* ** *   *** ***   **    ***

BnaA06g19880D       CAGTCGGGAA------------TCTTCATCA------AGAAGAAGACCAAGAAGACGGGG      258
BnaA06g18210D       CAGTCGTCACTTTCTTCGGGATCCATCGCTAAGCCTAAGAGGAAGACCACGAACACAACA      318
BnaC03g55590D       CAGTCGTCACTTTCTTCGGGATCCATCGCTA------AGAGGAAGACCACGAACACAACA      354
BnaA06g19890D       TCGTCATCACTTTCTTCAGGATCCGTTACTAAGA---------AGACCAAA---AAGCCG      264
BnaC01g24440D       TCGTCAT---------CAGGATCCGTTACAAAAA---------ATACCAAA---AAGCAG      285
                      ***                  * *    *            * ****     *

BnaA06g19880D       TGGCATCATCGTTGTTCGATATGTGGCCTGGAGTTTGAGATGGGACAGGCTCTTGGGGGT      318
BnaA06g18210D       AGGTCGCATCCTTGTCCGATATGTGGCGTGGAGTTTCCGATAGGACAGGCTCTTGGTGGT      378
BnaC03g55590D       AGGTCGCATCGTTGTCCGATATGTGGCGTGGAGTTTCCGATGGGACAGGCTCTTGGTGGT      414
BnaA06g19890D       TTGTCGCATACTTGTCCGATATGTGGCTTGGAGTTTCCGATGGGACAAGCTCTTGGGGGT      324
BnaC01g24440D       TCGTCACATACTTGTCCTATATGTGGCTTGGAATTTCCGATGGGACAAGCTCTCGGTGGT      345
                      *   ***  **** * ********* **** ***  *** ***** ***** ** ***

BnaA06g19880D       CACATGAGGAAGCACTGGAACAAGGAAGACTCTGGCGGGGCGTTGATTACACGCTCGTTT      378
BnaA06g18210D       CACATGAGGAAGCATAGGAACGAGGAAGAGGCTAGCGGCGCGTTGGTTGCACGCCCTTTT      438
BnaC03g55590D       CACATGAGGAAGCATAGGAACGAGGAAGAGGCTAGCGGCGCGTTGGTTACACGCTCTTTT      474
BnaA06g19890D       CACATGAGGAAGCATAGGAACGAGAAAGA---AAGAGGGGCGTTGGTTACACGCTCTTTT      381
BnaC01g24440D       CACATGAGAAAGCATAGGAACGATAAAGA---AAGCCTCGCGTTGGTTACACGCTCTTTT      402
                    ******** *****  ***** *  ****     *    ****** ** ***** * ***

BnaA06g19880D       TTCCCAGAGGCGGCG------ATGACTAGGACTTTGAAGGAAACGAGTAAAGAGAAGGGA      432
BnaA06g18210D       TTCCCTGAGGCGGCGACTTCGATGATTACGACATTGAAGAAATCGAGTAGTGGGAAAAGA      498
BnaC03g55590D       TTCCCTGAGGCGGCGACTTCGATGATTACGACATTGAAGAAATCTAGTAGTGGGAAAAGG      534
BnaA06g19890D       TTGCCGGAGGCG---------AAGTTTACGACTTTGAAGAAATCGAGCAGTGGGAAAAGA      432
BnaC01g24440D       TTGCCCGAAGTG---------TTGACTACAACTTTGAAGAAATCGAGTAGTGGGAAGAGA      453
                    ** ** ** * *           *  **  ** ****** ** * ** *  * ***  *

BnaA06g19880D       GTTGCGTGTTTGGAACCGGGATCAGATTCGATAGAGGGT---ATCAATTTGAAGTTAGAA      489
BnaA06g18210D       GTTGCGTGTTTTGACTTGGGGCCAGATTCTGTGGAGAGTAGCATCAATTTGAACTTGGAG      558
BnaC03g55590D       GTTGAGTGTTTTGACTTGGGGCCAGATTCTGCGGAGAGTAGCATCATTTGAACTTGGAGA      594
BnaA06g19890D       GTGGCGTGTTTTGGTATGGGATTGGATCCGATGGAGAGAGTGATCAACTTGAAGTTGGAG      492
BnaC01g24440D       GTGGCGTGTTTTGACTCGGAATTGGATTCGATGGAGGGTATAATCAATTTGAATTTGGAG      513
                    ** * ****** *    **     *** *    *** *    ***** ***** ** **
```

```
BnaA06g19880D       TTGGGAAGAAAGATGTACTGA        510
BnaA06g18210D       TTAGGAAGTAGCATGTACTGA        579
BnaC03g55590D       TTAGGAAGAAGCATGTACTGA        615
BnaA06g19890D       TTGGGAAGAACAATCTATTGA        513
BnaC01g24440D       TTGGGAATATCAATTTATTGA        534
                    ** ****     ** ** ***
```

62 nucleotide differences and three inserts between A06g19890D and C01g24440D
No SRA coverage found for BnaC03g55590D.

2i-C

Zat5-like

```
MyBnaA04g16320D        ATGATGAGTCAAGATCAT---GTTGGTAGTGACCAGACGCAAATCATAAAGGGTAAGCGT    57
MyBnaCnng66450D        ATGATGAGTCAAGATCAT---GTTGGTAGTGACCAGACGCAAATCATAAAGGGTAAGCGT    57
MyBnaA07g13700D        ATGATGGGTCAAGATCATGAGGTTGGTAGTGATCAGACGCAAATCATTAAGGGGAAGCGT    60
MyBnaC04g16100D        ATGATGGGTCAAGATCATGAGGTTGGTAGTGATCAGACGCAAATCATTAAGGGGAAGCGT    60
                       ****** ***********   *********** ************** ***** ******

MyBnaA04g16320D        ACGAAGCGACAGAGATCATTGTCGTCGACCTTTTTGGTGGCGGCCGCAGCGGCTACCACC    117
MyBnaCnng66450D        ACGAAGCGACAGAGATCATCGTCGTCGACCTTTTTGGTGGCGGCCGCGGCGGCTACCACC    117
MyBnaA07g13700D        ACGAAGCGGCAGAGATCATCCTCTTCGACGTTTTTGGTGGCGGCGG---CGGCCCCCACA    117
MyBnaC04g16100D        ACGAAGCGGCAGAGATCATCCTCTTCGACGTTTTTGGTGGCGGCGG---CGGCGACCACA    117
                       ******** **********  ** ***** ************** *   ****  ****

MyBnaA04g16320D        AACACCTCTTCGAGCTCATCAGCCGGGGATGGCGGCGGAGGGAGAGCAGTTTCCGATGAA    177
MyBnaCnng66450D        AACACCTCTTCAAACTCATCAGCCGGGGATGGCGGCGGAGGGAGAGCAGTTTCCGATGAA    177
MyBnaA07g13700D        ATCACCTCCACAAGTTCATCTGCC---------GGCGGAGAAAGAACAGCTTCAGAAGAA    168
MyBnaC04g16100D        ATCACCTCCACAAGTTCATCTGCC---------GACGGAGAAAGAACAGCTTCCGATGAA    168
                       * ******  * *  ***** ***         * *****  *** *** *** ** ***

MyBnaA04g16320D        TACAACTCGGCGGTTTCGTCTCCAGTGACCACAACTGATTGTACCGAAGAAGAGGAAGAC    237
MyBnaCnng66450D        TACAACTCGGCGGTTTCGTCTCCAGTGACCACAACTGATTGTACCGAAGAAGAGGAAGAC    237
MyBnaA07g13700D        TACAACTCGGTAGTTTCGTCTCCGGTTACTACAACTGATTGTACAGAAGAAGAGGAAGAC    228
MyBnaC04g16100D        TACAACTCGGTAGTTTCGTCTCCGGTTACTACAACTGATTGTACAGAAGAAGAGGAAGAC    228
                       **********  *********** ** ** ************** ***************

MyBnaA04g16320D        ATGGCGATTTGTCTCATCATGCTTGCGCGCGGAGCGGCTCTTTCG---------CCAGAT    288
MyBnaCnng66450D        ATGGCGATTTGTCTCATCATGCTTGCGCGCGGAGCGGCTCTTTCG---------CCAGAT    288
MyBnaA07g13700D        ATGGCGATTTGTCTCATCATGCTTGCACGTGGAGCTGCTCCATCGCCGCCGCTGCCGGAT    288
MyBnaC04g16100D        ATGGCGATTTGTCTCATCATGCTTGCACGTGGAGCTGCTCCATCGCCGCCGCTGCCGGAT    288
                       ************************** ** ***** ****  ***         ** ***

MyBnaA04g16320D        CTCAAGAATTCGAGAAAAGCTGATAAAACTTTTTCTCCG------------GCAGAGAAT    336
MyBnaCnng66450D        CTCAAGAATTCGAGAAAAGCTGATAAAACTCTTTCGTCG------------GCAGAGAAT    336
MyBnaA07g13700D        CTCAAGAATTCTACCAAAACTGACAAAAATCTTTATCAGAA---------------GAAT    333
MyBnaC04g16100D        CTAAAGAATTCTACCAAAACTGACAAAAATCTTCATCTGAAGACTTCGTCCTCGGAGAAT    348
                       ** ******** *  *** **** **** * **     *                 ****

MyBnaA04g16320D        TCGAGTTTCTTTGTCTACGAGTGTAAAACATGTAGCCGGACGTTCTCGTCGTTCCAAGCT    396
MyBnaCnng66450D        TCGAGTTTCTTTGTCTACGAGTGTAAAACATGTAGCCGGACGTTCTCGTCGTTCCAAGCT    396
MyBnaA07g13700D        TCTAGTTTCTATGTCTACGAGTGTAAAACATGTAACCGGACGTTCCCGTCGTTCCAAGCT    393
MyBnaC04g16100D        TCTAGTTTCTACGTCTACGAGTGTAAAACATGTAACCGGACGTTCCCGTCGTTTCAAGCT    408
                       ** *******  ********************** ********** ******* ******

MyBnaA04g16320D        CTCGGTGGACACAGGGCGAGCCACAAGAAACCTAGAGTGTCGATAGAAGAAAAGACTAAA    456
MyBnaCnng66450D        CTCGGTGGACACAGGGCGAGCCACAAGAAACCTAGAGTGTCGATAGAGGAAAAGACTAAA    456
MyBnaA07g13700D        CTTGGTGGACACAGGGCGAGCCACAAGAAGCCGAGAGCGTCGATAGACGAAAAAGCTAAA    453
MyBnaC04g16100D        CTGGGTGGACACAGGGCGAGCCACAAGAAGCCTAGAGCGTCGATAGACGAAAAAGCTAAA    468
                       ** ************************** ** **** ********* *****  *****

MyBnaA04g16320D        CTACCCCTGATGCAGGCCAAGTCTAGTGGGTCAGAGGAAGGGCAAAAAAATAATTTCAAA    516
MyBnaCnng66450D        CTACCCCTGATGCAGGCCAAGTCTAGTGGGTCAGAGGAAGGGCAAAAATATAATTTCAAA    516
MyBnaA07g13700D        GTACCCCTTACGCAGCTCAAGTCTAGTGCATCAGAGGAAGGGCAAAAAGGTCATTTTAAA    513
MyBnaC04g16100D        GTACCCCTTACGCAGCTCAAGTCTAGTGCATCAGAGGAAGGGCAAAAAAGTCATTTTAAA    528
                        ******* * ****  ***********  ******************  * **** ***

MyBnaA04g16320D        GTGTTTGGTTCATCCCTAGCTTTGCTGTCAAGTAACATCATCATCAGCAAGGCAAACAAA    576
MyBnaCnng66450D        GTGTTTGGTTCATCCCTAGCTTCGCTGTCAAGTAACATCATCATCAGCAAGGCAAACAAA    576
MyBnaA07g13700D        GTTTCCGGCCCAGCCCTTGCTTCAAAGGCAAGTAACATCATCATCAGCAAAGCAAACAAA    573
MyBnaC04g16100D        GTTTCCGGCCCAGCCCTAGCTTCGAAGGCAAGTAACATCATCATCAGCAAAGCAAACAAA    588
                       ** *  **  ** **** ****    * ********************** *********

MyBnaA04g16320D        GTACACGAGTGTTCGATCTGCGGTTCAGAGTTCACTTCGGGACAAGCCCTCGGCGGCCAC    636
MyBnaCnng66450D        GTACACGAGTGTTCGATCTGCGGTTCAGAGTTCACTTCGGGACAAGCCCTCGGCGGCCAC    636
MyBnaA07g13700D        GTACACGAGTGTTCGATCTGTGGTTCTGAGTTCACTTCGGGCCAAGCACTCGGAGGCCAC    633
MyBnaC04g16100D        GTACACGAGTGTTCGATCTGTGGTTCTGAGTTCACTTCAGGCCAAGCACTCGGAGGCCAC    648
                       ******************** ***** *********** ** ***** ***** ******
```

```
MyBnaA04g16320D      ATGAGGCGGCACAGGACAGCCACCACTGCGGTAA---TACCGGTCGCTACCACGGAAGTT     693
MyBnaCnng66450D      ATGAGGCGGCACAGGACAGCCACCACTGCGGTAA---TACCGGTCGCTACCACGGAAGTT     693
MyBnaA07g13700D      ATGAGGCGGCACAGGACAGTTACCAACGTGGTTAGCAGCCCGGTCACTGCAGCAGAAGTG     693
MyBnaC04g16100D      ATGAGGCGGCACAGGACAGTTACCAACGTGGTTAGCAGCCCGGTCAGTACAGCAGAAGTG     708
                     *******************  ****  * *** *     ******  * *  * *****

MyBnaA04g16320D      AGCAGAAACAGTACAGAAGAGGAGACTGAGAATTTGAGCTCTTACATTGAGCAAAGGAAA     753
MyBnaCnng66450D      AGCAGAAACAGTACAGAAGAGGAGACTGAGAATTTGAGCTCTTACATTGAGCAGAGGAAA     753
MyBnaA07g13700D      AGCAGAAACAGTACAGATGAAGAGACTGAGAATTTGAGCCGTTCGATGGAACAGAGAAAA     753
MyBnaC04g16100D      AGCAGAAACAGTACAGAAGAAGAGACTGAGAATTTGAGCCGTTCGATGGAACAGAGAAAA     768
                     ***************** ** ******************  **  ** ** ** ** ***

MyBnaA04g16320D      TATTTACCGCTGGATCTTAATCTACCGGCACCAGAAGATGATCTAAGAGAGTCCAAGTTT     813
MyBnaCnng66450D      TATTTACCGCTGGATCTTAATCTACCGGCACCAGAAGATGATCTAAGAGAGTCCAAGTTT     813
MyBnaA07g13700D      TATCTACCGTTGGATCTTAATCTACCGGCACCAGAAGATGATCTAAGAGAATCAAAGTTT     813
MyBnaC04g16100D      TATCTACCGTTAGATCTTAATCTACCGGCACCAGAAGATGATCTAAGAGAATCGAAGTTT     828
                     *** ***** * ************************************** ** ******

MyBnaA04g16320D      CAAGGGATTGTGTTCTC---AACCACACCAGCGTTAATAGACTGTCATTACTAG 864
MyBnaCnng66450D      CAAGGGATTGTGTTCTC---AACCACACCAGCGTTAATAGATTGTCATTACTAG 864
MyBnaA07g13700D      CAAGGGATAGTGTTCTCAGCAACCACACCAGCGTTAATAGATTGCCATTACTAG 867
MyBnaC04g16100D      CAAAGGATAGTGTTCTCAGCAACCACACCAGCGTTAATAGATTGCCATTACTAG 882
                     *** **** ********   ********************* ** *********
```

9 nucleotide differences between MyBnaA04g16320D and MyBnaCnng66450D
18 nucleotide differences and 1 insert found between MyBnaA07g13700D and MyBnaC04g16100D

Zat15-like

```
MyBnaA01g31690D    ATGGAAACAGCCGAGGAGGCGATATCGGCAGCTAAGGCGCAAGCCTTGATCATTAAAGGG    60
MyBnaA05g28380D    ATGGAAACAGCAGAGGAGGCGATATCGGCAGCTAAGGAGCAAGCCTTGATCCTTAAAGGG    60
MyBnaC05g42550D    ATGGAGACAGCAGAGGAGGCGATATCGGCAGCTAAAGAGCAAGCCTTGATCCTTAAAGGG    60
                   ***** ***** *********************** * ************* ********

MyBnaA01g31690D    AAGAGGACTAAGAGGCAGCGTCCTCAGTCCCCAATCCCTTTCTCTATTGTCCCTCCTATG    120
MyBnaA05g28380D    AAGAGGACTAAGAGGCAGCGTCTGCAGTCCCCAATCCCTTTCTCTATCGTCCCTCCTATG    120
MyBnaC05g42550D    AAGAGGACTAAGAGGCAGCGTCTGCAGTCCCCAATCCCTTTCTCTATCGTCCCTCCTATG    120
                   **********************  *********************** ************

MyBnaA01g31690D    TCTTCTCAAGAACCGGATACCCAAGAAGAGTCCACTAGTCTTGTTGCCAAGGAGAAGAGT    180
MyBnaA05g28380D    TCTTCTCAAGAACCTGATGTGGAAGAAGAGTCCACCAGTCTTGTTTCCAAGGAGAAGAGT    180
MyBnaC05g42550D    TCTTCTCAAGAACCTGATGTGGAAGATGAGTCCACCAGTCTTGTTTCCAAGGAGAAGAGT    180
                   ************** ***    **** ******** ********* **************

MyBnaA01g31690D    CTCAATGATGAGATCAACTACAACAATAATAAGAATGATAACAACATTTTGAGCATTGGT    240
MyBnaA05g28380D    CTCAATGATGATATTAACACCAA------CAAGAATGATAACAACGTTTTGATCAATGGT    234
MyBnaC05g42550D    CTCAATGATGAGATCAACACCAA------CAAGAATGATAACAACATGTTAAGCAATGGT    234
                   *********** ** ***  ***       *************** * ** * ** ****

MyBnaA01g31690D    GTGACATCTTCATCTTCAACTTCTTCATCTTTCAACAATGCGACATTAAAGGCCGCGGCT    300
MyBnaA05g28380D    GTGACATCTCCTGCC---TCTTCATCTTCTAACAACAATGCAACATTAAAGACCGCCGCT    291
MyBnaC05g42550D    GTGACATCTCCTGCC---TCTTCATCTTCTAACAACAATGCAACATTAAAGACCGCCGCT    291
                   ********* *  *     **** ** ***  ********* ********* **** ***

MyBnaA01g31690D    GACGAAGAAGATCAAGATATGGCTAACTGTTTGATCCTCCTTGCCCAAGGACACTCTCTT    360
MyBnaA05g28380D    GACGAGGAAGACCAAGACATGGCTAATTGTTTGATCCTCCTCGCCCAAGGTCACTATACC    351
MyBnaC05g42550D    GACGAGGAAGACCAAGACATGGCTAATTGTTTGATCCTCCTCGCCCAAGGTCACTATACC    351
                   ***** ***** ***** ******** ************** ******** **** *

MyBnaA01g31690D    CCACACCAACA---ACCACAAACAAGACAACTTATGGTAAGTTACCAAGAATCCGGTAAC    417
MyBnaA05g28380D    CCTCAACAACAACCACAACAAACAAGACAGTTTATGATGAGTTACCAAGAATCTGGTAAT    411
MyBnaC05g42550D    CCTCAACAACAACCACAACAAACAAGACAGTTTATGATGAGTTACCAAGAATCTGGTAAT    411
                   ** ** *****   ** ************  ***** * ************** *****

MyBnaA01g31690D    AACAACAACAATGCTTATAGATCTAGCAGCAGGAGATTTCTAGAGACATCTTCATCAAAC    477
MyBnaA05g28380D    AACAACAACAATGCGTATAGATCTAGCAGCAGAAGGTTCCTCGAGACGTCTTCACCTAAT    471
MyBnaC05g42550D    AACAATAACAATGCGTATAGATCTAGCAGCAGAAGGTTTCTCGAGACAT------CTAAT    465
                   ***** ******** ***************** ** ** ** ***** *      * **

MyBnaA01g31690D    GGGACCACTACAAGTGGAGGCAGAGCTGGTTACTATGTTTACCAATGCAAAACATGTGAC    537
MyBnaA05g28380D    GGAACCA---CGAGTGGAGGCAGAGCCGGTTACTATGTTTACCAATGCAAAACGTGTGAC    528
MyBnaC05g42550D    GGGACCA---CGAGTGGAGGCAGAGCCGGTTACTATGTTTACCAATGCAAAACGTGTGAC    522
                   ** ****   * ************** ************************** ******

MyBnaA01g31690D    CGGACTTTTCCTTCTTTCCAAGCTCTTGGTGGCCATAGAGCCAGCCACAAGAAGCCTAAA    597
MyBnaA05g28380D    CGGACTTTCCCTTCTTTCCAAGCTCTTGGTGGCCATAGAGCCAGCCACAAGAAGCCTAAA    588
MyBnaC05g42550D    CGGACTTTTCCTTCTTTCCAAGCTCTTGGTGGCCATAGAGCCAGCCACAAGAAGCCTAAA    582
                   ******** ***************************************************

MyBnaA01g31690D    GCCGCTCCGGGTCTTC---------ATGACCTCAAGAAGTCTATCTACAACGACGCCGTT    648
MyBnaA05g28380D    GCCGCAGCGGGTCTTCACTCCGACCATGACCTCAAGAAGTCTATATACAACGACGCCGTT    648
MyBnaC05g42550D    GCCGCAGCGGGTCTTCACTCCGACCATGACCTCAAGAAGTCTATCTACAACGACGCCGTT    642
                   *****  *********         ******************* ***************

MyBnaA01g31690D    TCTCATCATCTCAACAACGTCCTCACCACAACTCCTAACAACAATAATAACCATAGATCG    708
MyBnaA05g28380D    TCTCTTCATCTCAACAACGTCCCCACCGCAACTCCTAACAACAATAGTAGCCACAGGTCG    708
MyBnaC05g42550D    TCTCTTCATCTCAACAACGTCCCCGCCGCAACTCCTAACAATAATAGTAGCCACAGGTCG    702
                   **** ***************** * ** ************* **** ** *** ** ***

MyBnaA01g31690D    CTTGTGGTGCACGGTAAAGCGAATAACAATAAAGTCCACGAATGTGGAATATGTGGAGCC    768
MyBnaA05g28380D    CTTGTGGTATACGGCAAAGCGGGTAACAATAAAGTCCATGAATGTGGGATATGTGGAGCT    768
MyBnaC05g42550D    CTTGTGGTGTACGGCAAAGCGAATAACAATAAAGTCCATGAATGTGGGATATGTGGAGCT    762
                   ********  **** ******  *************** ******** ***********

MyBnaA01g31690D    GAGTTCACGTCCGGACAAGCCTTAGGTGGTCACATGAGACGCCATAGAGGCGCAGGGGTA    828
MyBnaA05g28380D    GAGTTCACGTCCGGACAAGCCTTAGGTGGTCACATGAGACGTCATAGAGGCGCAGTGGTG    828
MyBnaC05g42550D    GAGTTCACATCCGGACAAGCCTTAGGTGGTCACATGAGACGTCATAGAGGCGCAGTGGTG    822
                   ******** ******************************** ************* ***
```

```
MyBnaA01g31690D       GCAGCCAC---AGCCACCCCAACCGCGACATTAGCACTTCCGGCCAGTGCGGCTACCGCA     885
MyBnaA05g28380D       GCCTCAGCAGCATCCGCTTCCACCGCAACGGTTAGGGTTGCGGCCACTGCGGGTACAGCA     888
MyBnaC05g42550D       GCCTCAGCAGCATCCGCTTCCACCGCAACGGTTAGGGTTGCGGCCACTGCGGGTACAGCA     882
                      **  *  *   * ** *  * ***** **  *     ** ****** ***** *** ***

MyBnaA01g31690D       AACACGGTTTTGTCATTGTCGCCTATGTCGTTCGATCAGTTGTCTGACGGTCCGGTTTAT     945
MyBnaA05g28380D       AACACGGCCTTGTCATTGTCACCTATGTCGTTTGACCACATGT---------CGGTTCAT     939
MyBnaC05g42550D       AACACGGCTTTGTCATTATCACCTATGTCGTTTGACCAAATGT---------CGGTTCAT     933
                      *******  ******** ** *********** ** **  ***         ***** **

MyBnaA01g31690D       CCGGTTCAGGCTCCGGTTAAGAGAGCTAGGAGTGCGGTTGTGTCGTTGGATTTAGATCTG     1005
MyBnaA05g28380D       CCGGTTCAGGGTCCGGTTAAGAGAGCGAGGAGTGCGGTTGTGTCGTTAGATTTGGATCTG     999
MyBnaC05g42550D       CCGGTTCAGGCTCCGGTTAAGAGAGCGAGGAGCGCGGTTGTGTCGTTAGATTTGGATCTG     993
                      ********** *************** ***** ************** ***** ******

MyBnaA01g31690D       AATCTACCAGCCCCGGAAGATGAGAACCGGGTCAACGGATTAAGTGTTGCTTCAAAGCAA     1065
MyBnaA05g28380D       AATCTACCTGCCCCGGAAGATGTGAATCGGGTCAACGGGTTAAGCTTTGCTTCAAAGCAA     1059
MyBnaC05g42550D       AATCTACCTGCCTCGGAAGATGTGAATCGAGTCAACGGATTAAGCTTTGCTTCAAAGCAA     1053
                      ******** *** ********* *** ** ******** *****  **************

MyBnaA01g31690D       G------------AACATGGACATGAACAAACACAAGGGAGAGAAGAACAAAAGTCTATT     1113
MyBnaA05g28380D       GAACAGG------AACATGAACATGAACAAACGCAACAGAGAGAAGAACAAAAGTCTATT     1113
MyBnaC05g42550D       GAACAGGAACAGGAACATGAACATGAACAAACACATCAGAGAGAAGAACAAAAAACTCTT     1113
                      *            ****** ************ **   ***************  ** **

MyBnaA01g31690D       GTTTTGTCTTCTGCTCCTACATTGGTGGATTGCTATTACTGA------------------     1155
MyBnaA05g28380D       GTTTTGTCTTCGGCTCCTACATTGGTGGATTGCCATTACTAA------------------     1155
MyBnaC05g42550D       GTTTTGTCTTCCGCTCCTACATTGGTGGATTGCCATTACTAAGTCAAATATTTGATACCA     1173
                      *********** ********************* ****** *

MyBnaA01g31690D       ---------------  1155
MyBnaA05g28380D       ---------------  1155
MyBnaC05g42550D       AAATTCGTTTATTAA  1188
```

130 nucleotide differences and 7 inserts differ between MyBnaA01g31690D and MyBnaA05g28380D
31 nucleotide differences and two inserts differ between MyBnaA05g28380D and MyBnaC05g42550D

Zat14-like

```
BnaAnng00680D        ATGACAAGTTTTCATGAGGAGACGCGTCTAGTACTCCTCACCAAGGGGAAACGAACCAAA    60
BnaC02g03060D        ATGACAAGTTTTCACGAGGAGACGCGTCTAGTACTCCTCACCAAGGGGAAACGAACCAAA    60
BnaA10g26510D        ATGACAAGTGTTCATGAGGAGATGCGTCATGTACTCCTCATCAAAGGCAAACGCACCAAA    60
MyBnaA03g46860D      ATGACAAGTGTTCATGAGGAGACTCGTCTAGTACTCCTCGTCAAAGGCAAACGCACCAAA    60
BnaCnng02770D        ATGACAAGTGTTCATGAGGAGACTCGTCTAGTACTCCTCGTCAAAGGCAAACGCACCAAA    60
MyBnaA03g00910D      ATGACAAGTGTTCATGAGGAGACGCGTCTAGTACTCCTCATCAAGGGCAAACGTACCAAA    60
MyBnaC03g01240D      ATGACAAGTGTTCATGAGGAGACGCGTCTAGTACTCCTCATCAAGGGCAAACGTACCAAA    60
                     ********* **** *******  ****  *********  *** ** ***** ******

BnaAnng00680D        CGTCCTCGATCTTCGTCTCCTCATATGAACGCAGAAGCAGTGTCCGGCGTTTGCAGCGAG    120
BnaC02g03060D        CGTCCTCGATCTGAGTCTCCTCATATGAACGCAGAAGCAGTGTCCGGCGTTTGCAGCG--    118
BnaA10g26510D        CGTCAGCGATCTGCGTCTCCTCATATGAACGCAGAAGCAGTGTCTGGCGTTTGCAGCGAG    120
MyBnaA03g46860D      CGTCAGCGATCTGCGTCTCCTCATATGAACGCAGAAGCAGTGTCTGGCGTTTGCAGCGAG    120
BnaCnng02770D        CGTCAGCGATCTGCGTCTCCTCATATGAACGCAGAAGCAGTGTCTGGCGTTTGCAGCGAG    120
MyBnaA03g00910D      CGTCAGCGATCTGTGTCCCCGCATATGAACGCAGAAGCAATGTCCAGCGTTTGCAAAGAG    120
MyBnaC03g01240D      CGTCAGCGATCTGCGTCCCCTCATATCAAGGCAGAAGTAATGTCCAGTGTTTGCAACGAG    120
                     ****  ******  *** ** ***** ** ******* * ****  * *******  *

BnaAnng00680D        GACCGATCACTAGAGGCAAAAGA---AGGAGCTGGTGAAGTTGAATTCCAGGGAGCAACG    177
BnaC02g03060D        -ACCGATCACTAGGGGCAAAAGAAGAAGAAGCTGATGAAGTTGAGTTCCAGGGAGCTACG    177
BnaA10g26510D        GAACGATCACTGGAGGCAAGAGAAGATGGAGCTGGTGAAATCGAGTTCCAGGGAGCTACG    180
MyBnaA03g46860D      GAACCATCACTGGAGGCAAGAGAAGAAGGAGCTGGTGAAGTCGAGTTCCAGGGAGCTACG    180
BnaCnng02770D        GAACCATCACTGGAGGCAAGAGAAGAAGGAGCTGGTGAAGTCGAGTTCCAGGGAGCTACG    180
MyBnaA03g00910D      GAACGATCACTGGAGGCAAGAGA---AGGAGTTGGTGAAATCGAATTCCGGGGAGCTACG    177
MyBnaC03g01240D      GAACGATCACTGGAGGCAAGAGAAGAAGGAGCTGGTGAAATCGAATTCCGGGGAGCTACG    180
                      * * ****** * ***** ***     * ** ** **** * ** **** ****** ***

BnaAnng00680D        GACGAAGACCAAGACATGGCGAATTGTCTGATGCTATTGTCGCAAGGACATAAAGAAAAG    237
BnaC02g03060D        GACGAAGATCAAGACATGGCGAACTGTCTGATGCTATTGTCGCAAGGACACAAAGCAAAG    237
BnaA10g26510D        GAGGAAGACCAAGACATGGCGAACTGTCTGATGTTATTGTCACAAGGACACAAAGCAAAT    240
MyBnaA03g46860D      GAGGAAGACCAAGACATGGCGAACTGTCTGATGTTATTGTCACAAGGACACAAAGCAAAT    240
BnaCnng02770D        GAGGAAGACCAAGACATGGCGAACTGTCTGATGTTATTGTCACAAGGACACAAAGCAAAT    240
MyBnaA03g00910D      GACGAAGACCAAGACATGGCGAATTGTCTAATGTTGTTGTCGCAAGGACATAAATCAAAC    237
MyBnaC03g01240D      GACGAAGACCAAGACATGGCGAATTGTCTAATGTTGTTGTCGCAAGGACATAAATCAAAC    240
                     ** ***** ************** ***** *** * ***** ******** ***  ***

BnaAnng00680D        AGTAGTGGCGATCATTCATCGACGCTGAAGATTGGTTTCTTGACAGACAAGAAACCGGTG    297
BnaC02g03060D        AGTAGTGGCGATCATTTGTCGACGCAGAAGACTGGTTTCTTGAGCGACAAGAAACCGGTG    297
BnaA10g26510D        GGCAGTGGAGATCATTCGTCGACGCATAAACTTGATTTCTTGAGCTACAAGAATCCGGCT    300
MyBnaA03g46860D      GGCAGTGGAGATCATTCGTCGACGCACAAAATTGATTTCTTGTGCAAGAAGAAACCAGTA    300
BnaCnng02770D        GGCAGTGGAGATCATTCGTCGACGCACAAAATTGATTTCTTGTGCAAGAAGAAACCAGTA    300
MyBnaA03g00910D      ACTAGTGGAGATCCTTTGTTGACGCAGAAAATTGGTTTCTTGAGCAACAAGAAACCGGTG    297
MyBnaC03g01240D      ACTAGTGGAGATCCTTTGTTGACGCAGAAAATTGGTTTCTTGAGCAACAAGAAACCGGTG    300
                        ***** **** **  * *****  **    ** *******     * ***** ** *

BnaAnng00680D        GCTTCTCTAGGTTTAGGGCTAGACGGTGTTTACCAGTGCAAAACGTGTGATAAAAGCTTC    357
BnaC02g03060D        GGTTCTCTAGGGTTAGGGCTAGACGGTGTTTACCCGTGCAAAACGTGTGATAAAAGCTTC    357
BnaA10g26510D        GCTTCTCTAGGTTTAGGTCTAGAGGGTGTTTACCAGTGCCAAACGTGTGACAAAAGCTTC    360
MyBnaA03g46860D      GCTTCTCTAGGTTTAGGGCTAGAGGGTGTTTACCAGTGCCAAACGTGTGACAAAAGCTTC    360
BnaCnng02770D        GCTTCTCTAGGTTTAGGGCTAGAGGGTGTTTACCAGTGCCAAACGTGTGACAAAAGCTTC    360
MyBnaA03g00910D      GCCTCTCTAGGGTTAGGGCTAAACGGTGTTTACCAGTGCAAAACGTGTGATAAAAGCTTC    357
MyBnaC03g01240D      GCCTCTCTAGGGTTAGGGATAAACGGTGTTTACCAGTGCAAAACGTGTGATAAAAGCTTC    360
                     *  ******** *****   ** * ********** **** ********** *********

BnaAnng00680D        CACTCGTTTCAAGCGCTAGGAGGACACAGGGCTAGCCATAAGAAGCCCAAAC--------    409
BnaC02g03060D        CACTCGTTTCAAGCGCTAGGAGGACACAGGGCTAGCCATAAGAAGTCCAAAC--------    409
BnaA10g26510D        CACTCGTTTCAAGCGCTAGGAGGGCACAGAGCTAGCCATAAGAAGCCCAAACTCGGAGCA    420
MyBnaA03g46860D      CACTCGTTTCAAGCGCTAGGAGGGCACAGAGCTAGCCATAAGAAGCCCAAACTCGGAGAA    420
BnaCnng02770D        CACTCGTTTCAAGCGCTAGGAGGGCACAGAGCTAGCCATAAGAAGCCCAAACTCGGAGAA    420
MyBnaA03g00910D      CACTCGTTTCAAGCGCTAGGAGGGCACAGGACTAGCCATAAGAAGCCTAAACTCGGAGCA    417
MyBnaC03g01240D      CACTCGTTTCAAGCGCTAGGAGGGCACAGGACTAGCCATAAGAAGCCCAAACTCGGAGCA    420
                     *********************** *****  ************** * ****
```

```
BnaAnng00680D      -------------GCGAACGAGAAGACTCTGCGTCCGCGGTTGAAACGGTGGAATCTGCA    456
BnaC02g03060D      -------------GCGACGAGAAGAACTCTGCGTCTGCGGTTGAAACGGTGGAATCTGCA    456
BnaA10g26510D      ATCGTTCTCAAATGCCACGAGAAGAAATCTTCATCAGCTTTT---GCGGTTGAAACGGCC    477
MyBnaA03g46860D    AACGTTCTCAAATGCCACGAGAAGAAATCTTCATCAGCTTTT---GCGGTTGAAACGGCC    477
BnaCnng02770D      AACGTTCTCAAATGCCACGAGAAGAAATCTTCATCAGCGTCT---GCGGTTGAAACGGCC    477
MyBnaA03g00910D    ATCATTCTCAAATGCGGCGAGAAGAAATCTGCGTCTGCGGTTAAAACAGTAGAAGCTGCG    477
MyBnaC03g01240D    ATCGTTCTCAAATGCGACGAGAAGAAATCTGCGTCTGCGGTTAAAACAGTTGAAGCTGCG    480
                                **  ********* *** * ** **   *    * ** *** * **

BnaAnng00680D      GAAGCTGTAGGAAGCTTTCTTTCTCTGCAAGTAAGTAGCAGTGATGGTAGCAAGAAACCT    516
BnaC02g03060D      GAAACTGTAGGAAGCTTTCTCTCTCTGCAAGTAACTAGCAGTGATGGTAGCAAGAAACCT    516
BnaA10g26510D      A------AAGGAAGCTTTCTTTCTCTGCAAGTAACTAGCAGTGATGGTAACAAGAAACCT    531
MyBnaA03g46860D    A------AAGGAAGCTTTCTTTCTCTGCAAGTAACTAGCAGTGATGGTAACAAGAAACCT    531
BnaCnng02770D      A------AAGGAAGCTTTCTTTCTCTGCAAGTAACTAGCAGTGATGGTAGCAAGAAACCG    531
MyBnaA03g00910D    AGAGTTGTAGGAAGCTTTCTTTCTCTGCAAGTAACTAGTAGTGAAGGTACCAAGAAACAG    537
MyBnaC03g01240D    AGAGTTGTAGGAAGCTTTCTTTCTCTACAAGTAACTAGCAGTGACGGTAGCAAGAAACAG    540
                           ************ ***** ******* *** ***** **** ********

BnaAnng00680D      GAAAAGACACATGAATGTTCGATCTGCAAGGCCGAGTTTTCTTCAGGACAAGCTTTGGGA    576
BnaC02g03060D      GAAAAAACACATGAATGTTCGATCTGCAAGGCCGAGTTTTCTTCAGGACAAGCTTTAGGA    576
BnaA10g26510D      GAAAAAACACATGAATGTTCGATCTGCAAGGCCGAGTTTTCTTCAGGACAAGCCTTAGGT    591
MyBnaA03g46860D    GAAAAAACACATGAATGTTCGATCTGCAAGGCCGAGTTTTCTTCAGGACAAGCCTTAGGT    591
BnaCnng02770D      GAAAAAACACATGAATGTTCGATCTGCAAGGCCGAGTTTTCATCAGGACAAGCCTTAGGT    591
MyBnaA03g00910D    GAAAAAACACATGAATGTTCGATCTGTAAGGCCGAGTTTTCTTCGGGACAAGCCTTAGGA    597
MyBnaC03g01240D    GAAAAAACACATGAATGTTCGATCTGCAAGGCCGAGTTTTCTTCAGGACAAGCCTTAGGA    600
                   ***** ******************** ************** ** ******** ** **

BnaAnng00680D      GGTCATATGAGGAGACATAGAGGTTTAAACGTAAACGCAAATGCTACTTCAATAACAAAA    636
BnaC02g03060D      GGTCATATGAGGAGACATAGAGGTTTAAACGTAAACGCAAACGCTACTTCAATAACCAAA    636
BnaA10g26510D      GGTCATATGAGGAGACATAGAGGTTTAATAGTAAACGCAAACGCTAC-------------    638
MyBnaA03g46860D    GGTCATATGAGGAGACATAGAGGTTTAATAGTAAACGCAAACGCTAC-------------    638
BnaCnng02770D      GGTCATATGAGGAGACATAGAGGTTTAATAGTAAACGCAAACGCTAC-------------    638
MyBnaA03g00910D    GGACATATGAGGAGACATAGAGGTTTAACATTGAATGCAAATGCTAACTCAACAACCAGA    657
MyBnaC03g01240D    GGACATATGAGGAGACATAGAGGTTTAACATTGAATGCAAATGCTAACTCAACAATCAGA    660
                   ** *************************   * ** ***** ****

BnaAnng00680D      GCCGTGTTATCATCAAGTCATCACCAAGAACCAATACGGCCAAAGAACTTTCTTGAGTTG    696
BnaC02g03060D      GCCCTGTTATCATCAAGTCATCACCAAGAACCTATACGGCCAAAGAACTTTCTGGAGTTG    696
BnaA10g26510D      -----------TTCAAGTCATCATCAAGAATCTATACGGCCAAAGAACTATCTGGAGTTG    687
MyBnaA03g46860D    -----------TTCAAGTCATCATCAAGAATCTATACGGCCAAAGAACTTTCTGGAGTTG    687
BnaCnng02770D      -----------TTCAAGTCATCATCAAGAATCTATACGGCCAAAGAACTTTCTGGAGTTG    687
MyBnaA03g00910D    ACAGAGATATCATCAAGTCATCATCAAGAATCTATACGGGAAAAGAACTTTATAGAGCTA    717
MyBnaC03g01240D    ACAGCGACATCATCAAGTCATAATCAAGAATCTATACGGGAAAATAACTTTATGGAGTTA    720
                               ********* * ****** * ******  *** **** * * *** *

BnaAnng00680D      GACCTGAACCTTCCAGCGCCAGAAGATGAACCAAAATTCGTGTTTGCGTCCAAAGATCAG    756
BnaC02g03060D      GATCTGAACCTTCCAGCGCCAGAAGATGAACCAAAATTCGTGTTTGCGTCCAAAGATCAG    756
BnaA10g26510D      GATCTGAATCTTCCAGCGCCAGAAGATGAATCCAAGTTCGTGTTTGCGTCCAAAGATCAG    747
MyBnaA03g46860D    GATCTGAATCTTCCAGCGCCAGAAGATGAATCCAAGTTCGTGTTTGCGTCCAAAGATCAG    747
BnaCnng02770D      GATCTGAATCTTCCAGCGCCAGAAGATGAATCCAAGTTCGTGTTTGCGTCCAAAGATCAG    747
MyBnaA03g00910D    GATCTGAATCTTCCAGCGCCAGAAGATGAACCCAAGTTCGTGTTTGCGTCCAAAGATCAG    777
MyBnaC03g01240D    GATCTGAATCTTCCAGCGCCAGAAGATGAACCCAAGTTCGTGTTTGCGTCCAAAGATCAG    780
                   ** ***** ********************* * ** ************************

BnaAnng00680D      ATGCTTCTCTTCGCGTCTG---CGTCCAATTCTTTGATTGATTGTCATCACTAA- 807
BnaC02g03060D      ATGCTTCTCTTCGCGTCTG---CGTCCAATTCTTTGATTGATTGTCATCACTAA- 807
BnaA10g26510D      ATTATTCTCTTCACGACCG---CGTCCAATTCTTTAATTGATTGTCATCATTGA- 798
MyBnaA03g46860D    ATTATTCTCTTCACGACCG---CGTCCAATTCTTTAATTGATTGTCATCATTGA- 798
BnaCnng02770D      ATTATTCTCTTCACGACCG---CGTCCAATTCTTTAATTGATTGTCATCATTGA- 798
MyBnaA03g00910D    ATTCTTCTCTTCGCGGCTGCAGCGTCCAATTCTTTGATTGATTGTCATCACTAA- 831
MyBnaC03g01240D    ATTATTCTCTTCACGGCTG---CGTCCAATTCTTTGATTGATTGTCATCACTAAT 832
                   **  ******** ** * *   ************* ************** * *
```

29 nucleotide and 2 inserts differ between BnaAnng00680D and BnaC02g03060D
4 nucleotide differences between BnaCnng02770D and MyBnaA03g46860D
13 nucleotide differences between BnaA10g26510D and MyBnaA03g46860D
25 nucleotides and 1 insert differs between MyBnaA03g00910D and MyBnaC03g01240D

At5g04390-like

```
MyBnaAnng17660D     ATGGAAGCGTTTGAAGAGGCAATAGCAGCCTCAAAGGAGCAATCACTGATCCTTAAAGGA     60
MyBnaA10g25770D     ATGGAAGCGTTTGTAGAGGCCATAGCGGCTTCAAAGGAGCAATCATTAATCTTTAAAGGG     60
MyBnaC09g50520D     ATGGAAGCGTTTGAAGAGGCCATAGCGGCTTCAAAGGAGCAATCATTGATCTTTAAAGGG     60
MyBnaA03g01240D     ATGGAAGCATTTGAAGAGGCGATAGCGGCCTCAAAAGAGCAAGCATTGATCCTTAAAGGG     60
MyBnaC03g71210D     ATGGAAGCATTTGAAGAGGCGATAGCGGCCTCAAAAGAGCAAGCATTGATCCTTAAAGGG     60
                    ******** **** ****** ***** ** ***** ****** ** * *** *******

MyBnaAnng17660D     AAGAGGACAAAGCGACAACGTCCACAATCCCCTGTTCCTTTCTCTAGC---TCTCCTCCT     117
MyBnaA10g25770D     AAGCGTACAAAGCGACAACGTCCACAGTCTCCCATTCCTTTCTCTATC---GCCCCTCCT     117
MyBnaC09g50520D     AAGCGTACAAAGCGACAACGTCCACAGTCTCCTATTCCTTTCTCTATCATCTCCCCTCCT     120
MyBnaA03g01240D     AAGCGTACAAAGCGACAACGTCCACAGTCTCCTATTCCTTTCTCTGTA---TCCCCTCCT     117
MyBnaC03g71210D     AAGCGTACAAAGCGACAACGTCCACAGTCTCCTATTCCTTTCTCTGTA---TCCCCTCCT     117
                    *** * ******************** ** **  ***********       * ******

MyBnaAnng17660D     ATAGTTTCGTGCCACGCACACGACATTGAAGAGGAATACACTGATCTTGATTCCAAGGAA     177
MyBnaA10g25770D     ATAGTTTCT------------------T------------------------CCCATGCA     135
MyBnaC09g50520D     ATAGTTTCT------------------T------------------------CCCATGCA     138
MyBnaA03g01240D     ATAGTTGAA------------------GAAGAAGTATCCAACGTTCTTGATTCCAAGGAA     159
MyBnaC03g71210D     ATAGTTGAA------------------GAAGAAGTATCCAACGTTCTTGATTCCAAGGAA     159
                    ******                                              ** * * *

MyBnaAnng17660D     AATGCCTTAGGCAATAATGTGGAGAACCACAACAAGGATGGTGTTATCACGTCTTCATCT     237
MyBnaA10g25770D     CGTGACA---------TTCTAGAAGAATCCAAAAAGGATGGTGTGATCACGTCTTCATCT     186
MyBnaC09g50520D     CATGACA---------TTCAAGAAGAATCCAAAAAAGATGGTGTGATCACATCTTCATCT     189
MyBnaA03g01240D     AATGA------------TGTAGCAAACCGCAAAAAAGATGGTGTGATCACGTCTTCATCT     207
MyBnaC03g71210D     AATGA------------TGTAGCAAACCGCAAAAAGGATGGTGTGGTCACGTCTTCATCT     207
                      **             *   *   *   *** ** ********  **** *********

MyBnaAnng17660D     TCGTCAGCCTCTTGGTCCTCTAACAACAACCCAACATTAAAGGCCGAAGAAGACGAGGAA     297
MyBnaA10g25770D     TCATCAGCCTCTTGGTTCTCTAACAACAACGCAACTTTGAAGGCTGAAGAAGACGAGGAA     246
MyBnaC09g50520D     TCATCAGCCTCTTGGTCCTCTAACAACAACGCAACTTTGAAGGCTGAAGAAGACGAAGAA     249
MyBnaA03g01240D     TCATCAGCCTCTTGGTCCTCTAACAACAACCCAACATTGAAGGCCGAAGAAGACGAGGAA     267
MyBnaC03g71210D     TCATCAGCATCTTGGTCCTCTAACAACAACCCAACGTTGAAGGGCGAAGAAGACGAGGAA     267
                    ** ***** ******* ************* **** ** ****  *********** ***

MyBnaAnng17660D     GATCTAGACATAGCCAGTTGTTTGATCCTCCTTTCCCGAGGCCACTCTCTTCCA------     351
MyBnaA10g25770D     GAGCAAGACATAGCCAACTGTTTGATCCTCTTGTCTCAAGGTCACTCTCTTCCCA-----     301
MyBnaC09g50520D     GAGCAAGAAATAGCCAACTGTTTGATTCTCTTGTCCCAAGGTCACTCTCTTCCCC-----     304
MyBnaA03g01240D     GATCAAGACATAGCCAATTGTTTGATACTCCTTTCCCAGGGACACTCTTTCCCTCAACAC     327
MyBnaC03g71210D     GATCAAGACGTAGCCAATTGTTTGATACTCCTTTCCCAGGGACACTCTTTCCCCCAACAC     327
                    ** * ***  ******  ******** *** * ** *  ** ****** * **

MyBnaAnng17660D     ------CAGCTCAAGATACCTAACCACGAAACAACGTACAACAATAAGACGTATAAATTT     405
MyBnaA10g25770D     ----------------TACCTAACCACGAAGCAA---ACAACAATAACACGTTTAGATTT     342
MyBnaC09g50520D     ----------------TACCTAACCACGAAGCAA---ACAACAATAACACGTATAGATTT     345
MyBnaA03g01240D     AACCAACAGCTCAAGATACCTCACCAAGAAATAA---ACAATAATAACACGTATAGATTT     384
MyBnaC03g71210D     AACCAACAGCTCAAGATACCTCACCAAGAAATAA---ACAATAATAACACGTATAGATTT     384
                                    ***** **** ***  **   **** ***** **** ** ****

MyBnaAnng17660D     TGCAGCAGGAGGTTCCTAGAGACTTCTTCATCTAACGGTGGCGGTAAAGCTGGTTACTAT     465
MyBnaA10g25770D     AGCAGCAGGAGGTTTCTAGAGACTTCTTCATCTAACGGTGGTGGCAAAGCCGGTTACTAC     402
MyBnaC09g50520D     AGCAGCAGGAGGTTTCTAGAGACTTCTTCATCTAACGGTGGTGACAAAGCCGGTTACTAC     405
MyBnaA03g01240D     AGCAGCAGGAGGTTTCTAGAGACTTCTTCATCAAACGGTGGTGGCAAATCAGGTTACTAC     444
MyBnaC03g71210D     AGCAGCAGGAGGTTTCTAGAGACTTCTTCATCAAACGGTGGTGGCAAATCAGGTTACTAC     444
                     ************* ***************** ******** *  *** * ********

MyBnaAnng17660D     GTTTATCAGTGCAAATCATGTGACCGGACCTTCTCTTCTTTTCAGGCTTTAGGTGGCCAT     525
MyBnaA10g25770D     GTTTATCAATGCAAAACTTGTGACCGGACCTTCCCTTCTTTCCAGGCTTTAGGCGGCCAT     462
MyBnaC09g50520D     GTTTATCAATGCAAAACATGTGACCGGACCTTCCCTTCTTTCCAGGCTTTAGGCGGCCAT     465
MyBnaA03g01240D     GTTTATCAGTGCAAAACATGTGACCGGACCTTCCCTTCTTTTCAGGCTCTAGGCGGCCAT     504
MyBnaC03g71210D     GTTTATCAGTGCAAAACATGTGACCGGACCTTCCCTTCTTTTCAGGCTCTAGGCGGCCAT     504
                    ******** ****** * *************** ******* ****** **** ******
```

```
MyBnaAnng17660D      AGAGCTAGCCACAAAAAACCTAAGGCCACCT---CCTTTTATTCCAACCTTGACCACCTC     582
MyBnaA10g25770D      AGAGCTAGCCACAAGAAACCTAAAGCCACGTTATCCTTATATTCCAACATTGAC---GTT     519
MyBnaC09g50520D      AGAGCTAGCCACAAGAAACCTAAAGCCACGTTATCCTCATACTCCAACATTGAC---GTT     522
MyBnaA03g01240D      AGAGCTAGCCATAAGAAACCTAGAGCCACCT---CTTTTTACTCCAACCTTGAC---GTA     558
MyBnaC03g71210D      AGAGCTAGCCACAAGAAACCTAGAGCCACCT---CCTTTTACTCCAACCTTGAC---CTA     558
                     *********** ** *******  ***** *    * *  ** ****** *****    *

MyBnaAnng17660D      AAGAAGAACATATACGAAAATGA------TTCACTCTCCACAACCACAACTATTTACAAT     636
MyBnaA10g25770D      AAGAAGAATATCTACGAAAGTGACGCCGTTTCACTCGTCACAACCTCAACTATTTACAAG     579
MyBnaC09g50520D      AAGAAGAATATCTACGAAAGTAACGCCGTTTCACTCGTCACAACCTCAACTATTTACAAG     582
MyBnaA03g01240D      AAGAAGAGTATCTACGAAAACGACGCCGCTTCACT---------CACAAATATTTACAAT     609
MyBnaC03g71210D      AAGAAGAGTATCTACGAAAACGACGCCGCTTCACTCGTTACAACCACAAATATTTACAAT     618
                     *******  ** *******   *      ******         * *** *********

MyBnaAnng17660D      ------AACAACAAGAATAGATCGCTTGTCGTGTACGGTAAGGCAGGTAACAACAAGGTT     690
MyBnaA10g25770D      ------AACAATAACAATAGATCGCTTGCCGTGTATGGTAAGGCTGGTAGCAATAAGGTT     633
MyBnaC09g50520D      ------AACAATAACAATAGATCGCTTGCCGTGTATGGTAAGGCTGGTAGCAATAAGGTT     636
MyBnaA03g01240D      AACAAGAACAATAACAATAGGTCGCTTGTTGCTTACGGAAAGGCAGGTAACAATAAAGTT     669
MyBnaC03g71210D      AACAACAAAAACAACAATAGATCGCTTGTTGCTTACGGAAAGGCAAATAACAATAAAGTT     678
                           ** ** ** ***** *******  *  ** ** *****   ** *** ** ***

MyBnaAnng17660D      CATGAATGTGGAATCTGTGGAGCCGAGTTTACGTCAGGACAAGCCTTAGGTGGCCACATG     750
MyBnaA10g25770D      CATGAATGTGGAGTCTGTGGAGCCGAGTTTACGTCCGGGCAAGCCTTAGGTGGCCACATG     693
MyBnaC09g50520D      CATGAATGTGGAGTCTGTGGAGCCGAGTTTACGTCCGGGCAAGCCTTAGGTGGCCACATG     696
MyBnaA03g01240D      CATGAATGTGGAATATGTGGAGCCGAGTTTACGTCAGGACAAGCCTTAGGTGGCCACATG     729
MyBnaC03g71210D      CATGAATGTGGAATTTGTGGAGCCGAGTTTACGTCTGGACAAGCCTTAGGTGGTCACATG     738
                     ************ * ******************** ** ************** ******

MyBnaAnng17660D      AGACGGCATAGAGGCGCGGTTGTTGTTGCT------------CCCACTGTGACGGTGGCC     798
MyBnaA10g25770D      AGACGGCATAGAGGCGCAGTGGTTATTGCTGCGGCGCCGGTTACCACCGTGACGGTGGCC     753
MyBnaC09g50520D      AGACGGCATAGAGGCGCGGTGGTTATTGCTGCGGCGCCGGTTACCACCGTGACAGTGGCC     756
MyBnaA03g01240D      AGACGGCATAGAGGTGCGGTGGTTGTGGCTGCGGCACCAGCTCCCATCGTGACGGTGGCC     789
MyBnaC03g71210D      AGACGGCATAGAGGTGCGGTGGTTGTGGCTGCGGCACCAGCTCCCATCGTGACGGTGGCC     798
                     ************** ** ** *** * ***            ***  ***** ******

MyBnaAnng17660D      TTAGCTGCAGCCAACACGGAGTTATCATTGTCTTCGATGTCGTTCGATCAAATATC----     854
MyBnaA10g25770D      ACGGCTGCTGCCAACACGGAGTTATCATTGTCTTCTATGTCGTTCGATCAAATATCCGAC     813
MyBnaC09g50520D      ACGGCTGCTGCCAACACGGAGTTATCATTGTCTTCTATGTCGTTCGATCAAATATCTGAC     816
MyBnaA03g01240D      GCGGCTGCGGCCAACACGGAGTTATCATTGTCTTCCATGTCGTATGATCAAATATCCGAG     849
MyBnaC03g71210D      GCGGCTGCGGCCAACACGGAGTTATCATTGTCTTCCATGTCGTATGATCAAATATCCGAC     858
                        ***** **************************  *******  ***********

MyBnaAnng17660D      --------------------------GACAAAGAGAGCTAAGAAGATGGTGGTGTCATTG     888
MyBnaA10g25770D      GGTCAAGATCATTTGGTGATGCCAGCTACAAAGAGAGCTAAGAAGACGGTCGTGTCATTG     873
MyBnaC09g50520D      GGTCAAGATCATTTGGTGATGCCAGCTACAAAGAGAGCTAAGAAGACGGTCGTGTCATTG     876
MyBnaA03g01240D      GGTCAAGATCATCTGGTGATGCCAGAGGCAAAGAAAGCTAAGAAGATGGTCGTGTCACTG     909
MyBnaC03g71210D      GGTCAAGATCATCTGGTGATGCCAGAGGCAAAGAAAGCTAAGAAGATGGTCGTGTCATTG     918
                                                ****** *********** *** ****** **

MyBnaAnng17660D      GATTTGGATCTGAATCTACCCGCACCGGAAGATGAGAATAGGGTCAACGGATTCAGCTTA     948
MyBnaA10g25770D      GATTTGGATCTGAATCTACCCGCATCCGAAGATGAGAATCGGGTCAACGGGTTTACCTTT     933
MyBnaC09g50520D      GATTTGGATCTGAATCTACCCGCACCCGAAGATGAGAATCGGGTCAACGGGTTTACCTTT     936
MyBnaA03g01240D      GATTTGGATCTGAATCTACCCGCACCGGAAGATGAGAATCGGGTCAACGGGTTGAGCTTG     969
MyBnaC03g71210D      GATTTGGATCTGAATCTACCTGCACCGGAAGATGAGAATAGGGTCAATGGGTTGAGCTTG     978
                     ******************** *** * ************ ******* ** ** * ***

MyBnaAnng17660D      GGTTTTAAGCAAAAACATGAA------CAAGAACATCAACAGACAAAGCAAAGAGATGAA     1002
MyBnaA10g25770D      GCTTTGAAGCAAAAACAGGAA------CAAGAACATCAACCAACGATGCAAAGAGAAGAA     987
MyBnaC09g50520D      GCTTTGAAGCAAAAACAGGAA------CAAGAACATCAACCAACGATGCAAAGAGAAGAA     990
MyBnaA03g01240D      ACTTTGAAGCAAAAACACGAACAAGAACAAGAACATCAACAGACAAAACAAAGAGAAGAA     1029
MyBnaC03g71210D      GCTTTGAAGCAAAAACACGAACAAGAACAAGAACATCAAGAGACGAAACAAAAAGAAGAA     1038
                       *** *********** ***      ************    ** *  **** *** ***

MyBnaAnng17660D      CCAAAGTGTCTTGTCTTGTCTCCTCCTACTTTGGTGGATTGCCATTACTGA     1053
MyBnaA10g25770D      CCAAAGTGTCTTCTCATGTCTGCTCCTACTTTGGTGGATTGCCGTTACTGA     1038
MyBnaC09g50520D      CCAAAGTGTCTTCACATGTCTGCTCCTACTTTGGTGGATTGCCATTACTG-     1040
MyBnaA03g01240D      CAAGTGTCTCTTGTCTTGTCTGCTCCTACTTTGGTGGATTGCTATTACTGA     1080
MyBnaC03g71210D      CCAGTGTCTCTTGTCTTGTCTGCTCCTACTTTGGTGGATTGCTATTACTGA     1089
                     * *   ** ****   * ***** ******************   ******
```

19 nucleotide differences between MyBnaA10g25770D and MyBnaC09g50520D.

2i-D

Zat10-like

```
BnaC07g11700D      ATGGCTCTCGAGGCTCTCAGTTCACCGAGACTAGCTTCTCCGATTCCTCCTGTTCCAA       60
BnaA09g27780D      ATGGCGCTCGAGGCTATCAGTTCACCGAGATTAGCTTCACCGGTCCCTCCTCTGTTCGAA       60
BnaC05g21480D      ATGGCGCTCGAGGCTATCAGTTCACCGAGACTAGCTTCTCCGGTCCCTCCTCTGTTCGAA       60
BnaA08g18790D      ATGGCACTCGAGGCTCTCAGTTCACCTAGACTAGCTTCTCCAGTTCCTCCTCTGTTCGAA       60
BnaC03g58080D      ATGGCGCTCGAGGCTCTTAGTTCACCGAGATTAGCTTCGCCGGTTCCTCCTCTGTTCGAA       60
                   ***** ********* * ******** *** ******* **  * ****** ***** **

BnaC07g11700D      GATTCTTCAAGATTCCATGGCGTCGAACAGTGGACCAAAGGTAAAAGATCTAAACGATCA       120
BnaA09g27780D      GATTCTTCAAGATTCCACGGCGTCGATCACTGGACCAAAGGTAAAAGATCTAAACGGTCC       120
BnaC05g21480D      GATTCTTCAAGATTTCACGGCGTCGAACACTGGACCAAGGGTAAAAGATCTAAACGATCC       120
BnaA08g18790D      GATTCTTCAAGATTCCACGGAGTCGAACACTGGACCAAAGGCAAACGTTCCAAAAGATCA       120
BnaC03g58080D      GACTCATCAAGATTCCATGGAGTCGAACACTGGACCAAAGGCAAACGTTCCAAGAGATCA       120
                   ** ** ******** ** ** ***** ** ******** ** *** * ** **  * **

BnaC07g11700D      AGATTCGATTTCAACCACCACAACCTCACTGAAGAAGAGGATCTCGCTTTCTGCCTTATG       180
BnaA09g27780D      AGATCCGACTTCCACCACAAGAACCTCACAGAAGAAGAGTATCTCGCTTTCTGCCTCTTG       180
BnaC05g21480D      AGATCCGACTTCCCCACAAGAACCTCACAGAAGAAGAGTATCTCGCTTTCTGCCTCTTG       180
BnaA08g18790D      AGATCCGATTTTAACCAACAAAACCTCACTGAAGAAGAGTATCTCGCTTTCTGCCTCTTG       180
BnaC03g58080D      AGATCCGATTTCCACCAACAAAACCTCACCGAAGAAGAGTATCTCGCTTTCTGCCTCTTG       180
                   **** *** **   ***  * ******** ********* ****************  **

BnaC07g11700D      CTCCTCGCTCGCGACGGCAA---------TCGTCAGCTTCTTCCTCTTCCTCCGGTGACG       231
BnaA09g27780D      CTCCTCGCTCGTGACGGCGATCGCAGCAACCGTAACCCTCTTCCTCCTCCTCCGGTGACG       240
BnaC05g21480D      CTCCTCGCTCGTGACGGAGATCGCAGCAACCGTAACCCTCTTCCTCCTCCTCCGGTGACG       240
BnaA08g18790D      CTTCTGGCCCGCGACGGCAACCGCAGCAACCGTCAGCCTCTTC------CTCCGGTGACA       234
BnaC03g58080D      CTTCTCGCCCGCGACGGCAACCGCAGCAACCGTCAGCCTCTTC------CTCTCCCTCCG       234
                   ** ** ** ** *****  *          *** * * *****      ***      *

BnaC07g11700D      GTAGTAGCTGAGAAGTCGTCG---TTGCGCTACAAGTGCAGCGTCTGCGACAAGTCTTTC       288
BnaA09g27780D      GTG---GCGGAGAAGTCGT------CAACCTACACGTGCAGCGTCTGCGACAAGTCCTTC       291
BnaC05g21480D      GTG---GGGGAGAAGTCGT------CAACCTACACGTGCAGCGTCTGCGACAAGTCCTTC       291
BnaA08g18790D      GCG---GCTGAGAAGTCGTCGTCGTCGTCCTACAAGTGCAGCGTCTGCGACAAGTCGTTC       291
BnaC03g58080D      GTG---GCTGAGAAGTCGTCGTCGTCGTCCTACAAGTGCAGCGTCTGCGACAAGTCGTTC       291
                   *     *  **********          ***** ********************* ***

BnaC07g11700D      TCTTCTTACCAAGCCCTCGGCGGACACAAAGCCAGCCACCGTAAAAACGTGTCACAGACT       348
BnaA09g27780D      TCGTCTTACCAAGCCCTCGGGGGACACAAGGCGAGCCACCGGAAAAACTTATCACAGACC       351
BnaC05g21480D      TCGTCTTACCAAGCACTCGGCGGACACAAGGCCAGTCACCGGAAAAACTTATCACAGACC       351
BnaA08g18790D      TCGTCTTACCAAGCTCTCGGCGGACACAAGGCGAGCCACAGGAAAAACTCATCACAGACT       351
BnaC03g58080D      TCGTCTTACCAAGCTCTCGGCGGACACAAGGCGAGCCACAGGAAAAACTCATCACAGACT       351
                   ** *********** ***** ******** ** ** *** * ******   ********

BnaC07g11700D      CATTCCGCCGGAGGAGATGACCAGTCGACGTCGTCAGCTACGACTGCATCCGCCGTGACG       408
BnaA09g27780D      CTATCCGGCGGAGGAGACGATCAATCGACGTCG---------ACCACATCCGCCGTGACA       402
BnaC05g21480D      CTATCCGGCGGAGGAGACGATCAATCAACGTCG---------ACCACATCCGCGGTGACA       402
BnaA08g18790D      CAATCCGGCGGAGGAGATGATCAGTCAACGTCGG---CGACCACCACGTCCGCCGTGACA       408
BnaC03g58080D      CAATCCGGCGGAGGAGATGATCAGTCTACGTCGG---CGACCACCACGTCCGCCGTGACA       408
                   *  **** ********* ** ** ** ******         **  * ***** *****

BnaC07g11700D      ACCGGAAGCGGGAAGTCGCACGTTTGCTCGATCTGTCAGAAGTCGTTTCCTTCAGGCCAA       468
BnaA09g27780D      ACCGGAAGTGGGAAGTCTCACGTCTGCTCGATCTGTCACAAGTCGTTTCCTTCCGGCCAA       462
BnaC05g21480D      ACCGGAAGTGGGAAGTCGCATGTTTGCTCGATCTGTCACAAGTCGTTTCCTTCCGGCCAA       462
BnaA08g18790D      ACCGGAAGCGGGAAGTCGCACGTGTGCACGATCTGTCACAAGTCGTTTCCCTCGGGACAA       468
BnaC03g58080D      ACCGGAAGCGGGAAGTCGCACGTTTGCACGATCTGTAACAAGTCGTTTCCTTCCGGACAA       468
                   ******** ******** ** ** *** ******** * *********** ** ** ***

BnaC07g11700D      GCTCTCGGCGGACACAAGCGGTGTCACTACGAAGGGAATAACAA---CACTAGTAGCGTG       525
BnaA09g27780D      GCTCTCGGCGGACACAAGCGGTGCCACTACGAGGGGAATAACAACAGTAGCAGCAGCGTG       522
BnaC05g21480D      GCTCTCGGCGGACACAAGCGGTGCCACTACGAGGGGAATAACAACAGTAGCAGCAGCGTG       522
BnaA08g18790D      GCTCTAGGCGGACACAAGCGGTGCCACTACGAAGGGAATAATAATAACACTAGTAGCGTG       528
BnaC03g58080D      GCTCTCGGCGGACACAAGCGGTGCCACTACGAAGGGAATAATAATAACACTAGTAGCGTG       528
                   ***** ***************** ******** ******** **    *  ** ******
```

```
BnaC07g11700D      TCTAACTCTGAAGGAGCGGGGTCCACCAGCCACGTCAGCAGCAGTCACCGCGGGTTTGAC      585
BnaA09g27780D      GCTAACTCCGAAGGAGCGGGGTCCACCAGCCACGTCAGCAGCGGTCACCGTGGGTTTGAC      582
BnaC05g21480D      GCTAACTCCGAAGGAGCGGGGTCCACCAGCCACGTCAGCAGCGGTCACCGTGGGTTTGAC      582
BnaA08g18790D      TCTAACTCGGAAGGAGCGGGGTCCACCAGCCACGTCAGCATCAGCCATCGTGGGTTTGAC      588
BnaC03g58080D      TCTAACTCCGAAGGAGCGGGGTCCACCAGCCACGTCAGCATCAGCCATCGTGGGTTTGAC      588
                    ******* ******************************* * * ** ** *********

BnaC07g11700D      CTTAATATCCCTCCGATACCGGAGTTCTCTACGGTCAACGGAGACGACGAAGTGATGAGC      645
BnaA09g27780D      CTTAACATCCCTCCTGTACCGGAATTCTCGCTGGTGAACGGAGACGACGAAGTGATGAGC      642
BnaC05g21480D      CTTAACATCCCTCCTGTACCGGAATTCTCGTTGGTGAACGGAGACGACGAAGTGATGAGC      642
BnaA08g18790D      CTTAATATCCCTCCGATACCGGAGTTCTCGCTGGTCAACGGAGACGACGAAGTGATGAGC      648
BnaC03g58080D      CTTAATATCCCTCCGATACCGGAGTTCTCGTTGGTCAACGGAGACGACGAAGTGATGAGC      648
                   ***** ********  ******* *****   *** ************************

BnaC07g11700D      CCTATGCCGGCGAAGAAGCCCCGGCTTGACTTCTTGGAGAAACTTAATTAG---   696
BnaA09g27780D      CCGATGCCGGCGAAGAAGCCCCGTTTTGACTTCTCGGAGAAAGCTTAA------   690
BnaC05g21480D      CCGATGCCGGCGAAGAAGCCCCGTTTTGACTTCTCGGAGAAAGCTTAA------   690
BnaA08g18790D      CCGATGCCGGCGAAGAAGCCCCGTTTTGACTTCTCGGAGAAACTCCAACATTGA   702
BnaC03g58080D      CCGATGCCGGCGAAGAAGCCCCGTTTTGACTTCTCGGAGAAACTCCAACATTGA   702
                   ** ********************  ********* *******    *
```

Comparison of BnaA08g18790D and BnaC03g58080D found 25 nucleotide differences.
Comparison of BnaA09g27780D and BnaC05g21480D found 19 nucleotide differences.
Comparison of BnaC07g11700D and all other transcripts in alignment finds 37 nucleotide differences unique to this sequence and five gaps.

AZF2-like

```
BnaA01g26030D      ATGGCTCTCGAAGCGATGAATTCTC------------CTTCGTTCACAGTCCGTAAAGAT      48
BnaC01g33270D      ATGGCTCTCGAAGCGATGAATTCTC------------CTTCGTTCACAGTCCGTAAAGAT      48
BnaA05g20490D      ATGGCTCTCGAAGCGATGAATTGTCCGACGAATTCTTCTTCGTTCACAGCACGGAAAGAT      60
BnaC05g33170D      ATGGCTCTCGAAGCGATGAATTGTCCGACGAATTCTTCTTCGTTCACAGCACGGGAAGAT      60
BnaA03g35310D      ATGGCTCTCGAAGCGATGAACTCTCCGACAACTACGTCCCCGTTCACAGTCCGAAAAGAT      60
BnaC03g73930D      ATGGCTCTCGAAGCGATGAATTCTCCGACAACTATGTCCTCGTTCACAGTCCGAAAAGAC      60
                   ******************** * **            *  *********  **  ****

BnaA01g26030D      AGAATCGCAGCAACAGACGATGATCTAACGAACGACGCCGTTTTCATGGAGCCTTGGCTG      108
BnaC01g33270D      AGAATCGAAGCAACAGAAGATGATCTGATGAACGACGCCGTTTTCATGGAGCCTTGGCTG      108
BnaA05g20490D      AGAAACGAACCAACAG---ATGATCTTACGCACGACGCCGTTTTCATGGAGCCTTGGCTG      117
BnaC05g33170D      AGAAACGAACCAACAG---ATGATCTTACGAACGACGCCGTTTTCATGGAGCCTTGGCTG      117
BnaA03g35310D      AGGATCGGAGCAACGG---ATGATCTGATGAACGACGCCGTTTTCTTGGAGCCTTGGCTG      117
BnaC03g73930D      AGGATCGGAGCAACGG---ATGATCTGATGAACGACGCCGTTTTCTTGGAGCCTTGGCTG      117
                   ** * ** * **** *   ******* * * ************** **************

BnaA01g26030D      AAACGCAAACGCACGAAACGCCAGCGTTCTCGCAGCCCTTCTCCGTCCACCTCTTCCTCC      168
BnaC01g33270D      AAACGCAAACGCTCGAAACGCCAGCGTTCTCGCAGCCCTTCTCCGTCCACCTCTTC---C      165
BnaA05g20490D      AAACGCAAGCGCTCGAAACGCCCGCGTTCACGCAGCCCTTC------------CTC---C      162
BnaC05g33170D      AAACGCAAGCGCTCGAAACGCCAGCGTTCTGGCAGCCCTTC------------CTC---C      162
BnaA03g35310D      AAACGCAAACGCTCCAAACGCCAGCGTTCTCCCAGCCCTTC------------TTCCTCC      165
BnaC03g73930D      AAACGCAAACGCTCGAAACGCCAGCGTTCTCCCAGCCCTTC------------CT---CC      162
                   ******** *** * ******* ******   *********             *    *

BnaA01g26030D      TCGCCGCCTCGATCTCGCCGCCCTAAATCCGAGAGTCAGGATCTCACTGAGGAAGAGTAT      228
BnaC01g33270D      TCGCCGCCTCGATCTCGTCGCCCTAAATCCGAGAGTCAGGATCTCACGGAGGAAGAGTAT      225
BnaA05g20490D      TCGCCGCCTCGTTCTCG---CCCTAAGTCTCAAATTCAGGATCTTGCGGAGGAGGAGTAT      219
BnaC05g33170D      TCGCCGCCTCGTTCTCG---CCCTAAATCTCAAATTCAGGATCTTGCGGAGGAAGAGTAC      219
BnaA03g35310D      ACGCCGCCTCGACTTCA---CGCTAAATCTCAAAGTCAGGATCCTTCGGAGGAAGAGTAT      222
BnaC03g73930D      TCGCCGCCTCGATCTCA---CGCTAAATCTCAAAGTCAGGATCCTTCGGAGGAAGAATAT      219
                    **********   **    * **** **  * * ********   * ***** ** **

BnaA01g26030D      CTCGCTCTCTGTCTCCTCAAGCTCGCTAAAGACAAACACTCGCC---------GCCGCCG      279
BnaC01g33270D      CTCGCTCTGTGTCTCCTCAAGCTCGCTAAAGACAAACACTCACC---------GCGGCC-      275
BnaA05g20490D      CTCGCTCTCTGTCTCATCATGCTCGCCAACGACCACCACCA------------AC---CC      264
BnaC05g33170D      CTCGCTCTCTGTCTCCTCATGCTCGCCAACAACCACCACCA------------AC---CC      264
BnaA03g35310D      CTCGCTCTCTGTCTCCTCATGCTCGCCAAAGACCAGCCGCGGACGCGATTTCAACCGTCA      282
BnaC03g73930D      CTCGCTCTCTGTCTCCTCATGCTCGCTAAAGACCAGCCGCGGACGCGATTCCAACCCTCA      279
                   ******** ****** *** ****** **  ** * *                 *   *

BnaA01g26030D      CAGCCGC---AGCCGCGAGAC---TCGACGAAGCTCTCGTACAAGTGTAGCGTTTGCGGA      333
BnaC01g33270D      -----GC---AGCCGCAAGAC---TCGACGAAGCTCTCGTACAAGTGTAGCGTTTGCGGA      324
BnaA05g20490D      AAGACGCAACCGCCGCAAGAATCCACCACAAAGCTTTCGCACAAGTGCAGCGTTTGCGGG      324
BnaC05g33170D      AAGAGGC---GGCCGCAAGAATCCACCACAAAGCTTTCGCACAAGTGCAGCGTTTGCGGG      321
BnaA03g35310D      CCGCCGC---CGCCTCAAGAAAGGACGACGAAGCTTTCGTATAACTGTAGCGTTTGCGGA      339
BnaC03g73930D      CTGCCGCCGCCGCCTCAAGCAAG---GACGAAGCTTTCGTATAACTGTAGCGTTTGCGGA      336
                        **    *** * **        ** ***** *** * ** ** ***********

BnaA01g26030D      AAAGCGTTTCCTTCGTATCAGGCGTTAGGCGGACACAAAGCGAGCCACCGAATCAAGCCT      393
BnaC01g33270D      AAAGCGTTTCCTTCGTATCAGGCGTTAGGCGGACACAAAGCGAGCCACCGAATCAAGCCT      384
BnaA05g20490D      AAAGCGTTTCCTTCTTACCAGGCGTTAGGCGGCCACAAAGCCAGCCACCGAATCAAGCCT      384
BnaC05g33170D      AAAGCGTTTCCTTCGTACCAGGCTTTAGGCGGCCACAAAGCCAGCCACCGAATAAAGCCT      381
BnaA03g35310D      AAAGCGTTTGTTTCGTATCAGGCTTTAGGCGGCCACAAAGCCAGCCACCGCATCAAGCCT      399
BnaC03g73930D      AAAGCGTTTCTTTCGTATCAGGCTTTAGGCGGTCACAAAGCCAGCCACCGAATCAAACCT      396
                   *********  *** ** ***** ******** ******** ******** ** ** ***

BnaA01g26030D      CTAACC---------GCCGATAATTCGACGTCTCCGATCATCGCCGGAGAGAAGCATCAC      444
BnaC01g33270D      CTAACC---------GCCGATAATTCAACATCTCCGATCATCGCCGGAGAGAAGCATCAC      435
BnaA05g20490D      CCAACCACAACCGCCGACGACGATTCAACAACTCCAACCATCGCCGTCG---CGCATCCG      441
BnaC05g33170D      CCAACCACAACCGCCGACGACGATTCGACACCTCCAACCATCGCCGTCG---CGCATCCG      438
BnaA03g35310D      CCAACC---------GCCGGTGATTCAACAGCTCCGAGCATCGCCGGAGAAAAGCATCTG      450
BnaC03g73930D      CCAACC---------GCCGGTGATTCAACAGCTCCGAGCATCGCCGGCGAAAAGCATCCG      447
                   * ****         * **   **** **  **** * ********  *    *****
```

```
BnaA01g26030D        AGTTCCGCCACCGTCCCTCCTTCCGGGAAGATTCACGAGTGCTCTATCTGCCGTAAAGTG        504
BnaC01g33270D        AGTTCCGCCACCGTCCCACCTTCCGGGAAGATTCACGAGTGCTCTATCTGCCGTAAAGTG        495
BnaA05g20490D        ACTTCCACCGCCATCGCACCTTCCGGGAAGATCCACAAGTGCTCCATCTGCCATAAAGTG        501
BnaC05g33170D        ACTTCCACCGCCATCGCACCTTCCGGGAAGATCCACGAGTGTTCCATCTGCCATAAAGTG        498
BnaA03g35310D        ACTTCCACAGCCATCGCTCCTTCAGGGAAGATCCACGAGTGTTCTATCTGTCATAAAGTG        510
BnaC03g73930D        ACTTCCACAACCATCGCTCCTTCCGGGAAGATCCACGAGTGTTCCATCTGCCATAAAGTG        507
                     * **** *  ** ** * ***** ******** *** **** ** ***** * *******

BnaA01g26030D        TTTCCGACGGGTCAAGCTCTGGGCGGCCACAAACGGTGCCACTACGAAGGGAACCTCG--        562
BnaC01g33270D        TTTCCGACGGGTCAAGCACTCGGCGGTCACAAACGCTGCCACTACGAAGGAAACCTCG--        553
BnaA05g20490D        TTTCCCACGGGTCAAGCTCTCGGCGGCCACAAACGCTGTCACTACGAAGGAACTATCGGC        561
BnaC05g33170D        TTTCCGACGGGTCAAGCTCTCGGCGGCCACAAACGCTGTCACTACGAAGGAACCATCGGA        558
BnaA03g35310D        TTTCCGACGGGTCAAGCTCTAGGCGGTCACAAACGCTGTCACTACGAAGGGAACCTCGGC        570
BnaC03g73930D        TTTCCGACGGGTCAAGCTCTAGGCGGCCACAAACGCTGTCACTACGAAGGGAACCTCGGC        567
                     ***** *********** ** ***** ******** ** *********** *   ***

BnaA01g26030D        -------GAGGAGGAAGCAAATCGATTAGCCAGAGTGGAAGCGTGTCGAGCACGGTTTCG        615
BnaC01g33270D        -------GAGGAGGAAGCAAAACGATTAGCCAGAGTGGAAGCGTGTCGAGCACGGTTTCG        606
BnaA05g20490D        GGCGGCG---GAGGAAGCAAGTCGGTTAGCCAGAGCGGAAGCGTGACAAGCACGGTTTCG        618
BnaC05g33170D        GGCGGCG---GAGGAAGCAAATCGGTTAGCCAGAGTGGAAGCGTGACGAGTACGGTTTCG        615
BnaA03g35310D        GGCGGCGGAGGAGGAAGCAAGTCGGTGAGCCACAGTGGAAGCGTGTCAAGTACGGTTTCG        630
BnaC03g73930D        GGCGGAGGCGGCGGAAGCAAGTCGGTGAGCCACAGTGGAAGCGTGTCAAGTACGGTTTCG        627
                               * ********   ** * ***** ** ********* * ** *********

BnaA01g26030D        GAAGATCGAAGCAACCGCGTGTTGATCGATCTGAACCTCCCGGCTTTACCGGAGCTCAGC        675
BnaC01g33270D        GAAGATCGAAGCAACCACGTGTTGATCGATCTAAACCTCCCGGCTTTACCGGAGCTCAGC        666
BnaA05g20490D        GAAGAACGAAGCAACCGCGTGTTCATTGATCTAAACCTTCCCGCGTTACCGGAGCTCAGC        678
BnaC05g33170D        GAAGAACGAAGCAACCGTGCGTTCATCGATTTAAACCTCCCGGCGTTACCGGAGCTCAGC        675
BnaA03g35310D        GAAGAACGAAGCAACCGTGTGATTATAGATCTGAATCTCCCGGCGTTACCGGAGCTCAGC        690
BnaC03g73930D        GAAGGACGAAGCAACCGTGTTATTATAGATCTGAATCTCCCGGCGTTACCGGAGCTCAGC        687
                     ****  **********   *    * ** *** * ** ** ** ** ***************

BnaA01g26030D        CTTCAT---CACAACCCAGTCGTCGACGATGAGATACTGAGTCCGTTGACCGGGAAGAAA        732
BnaC01g33270D        CTTCAT---CACAACCCAGTCGTCGACGAGGAGATACTGAGTCCGTTGACCGGGAAGAAA        723
BnaA05g20490D        CTTCATCATCACAACACAGTCGTCGACGAAGAGATTCAAAGTCCGTTGACCGGTAAAAAA        738
BnaC05g33170D        CTTCATCATCACAATCCAGTCGTCGACGAAGAGATTCAAAGTCCGTTGACCGGTAAAAAG        735
BnaA03g35310D        CTTCAT---CACGACCCAGTCGTCGACGATGAGATTCTAAGTCCGTTGACCGGTAAAAAA        747
BnaC03g73930D        CTTCAT---CACGACTCAGTCGTCGACGAAGAGATTCTAAGTCCGTTGACCGGTAAAAAA        744
                     ******    *** *  ************* ***** *  ************** ** **

BnaA01g26030D        CCGCTTTTGTTGACCGATCGTGACCAAGTCAT---CAAGAAAGAAGATTTATCTCTAAGA        789
BnaC01g33270D        CCGCTTTTGTTGACGGATCGTGACCAAGTCAT---CAAGAAAGAAGATTTATCTCTAAGA        780
BnaA05g20490D        CCGCTGTTGTCGACCGATCACGACAAAGTCATCATCAAGAAAGAAGATTTCTCCCTAAGA        798
BnaC05g33170D        CCGCTTTTGTTGACCGATCACGACAAAGCCAGCATCAAGAAAGAAGATTTCTCCCTAAGA        795
BnaA03g35310D        CCGCTTTTGTTGACCGGTCACGACCAAGTCAT---CAAAAAAGAAGATTTATCTCTAAGA        804
BnaC03g73930D        CCGCTTTTGTTGACCGGTCACGACCAAGTCAT---CAAGAAAGAAGATTTATCTCTAAGA        801
                     ***** **** *** * **   *** *** **      *** *********** ** ******

BnaA01g26030D        ATATAA    795
BnaC01g33270D        ATATAA    786
BnaA05g20490D        ATCTAA    804
BnaC05g33170D        ATCTAA    801
BnaA03g35310D        ATATAA    810
BnaC03g73930D        ATCTAA    807
                     ** ***
```

BnaA01g26030D and BnaC01g33270D contain 21 nucleotide differences plus one gap.
BnaC03g73930D and BnaA03g35310D contain 28 nucleotide differences and one 2 base gap.
BnaA05g20490D and BnaC05g33170D contain 34 nucleotide differences and one gap.

Zat13-like

```
BnaA01g21030D      ATGGCTCTCGACACTCTCAATTCTCCGAACTCCACGACCACCGCTCCCTCTCCATTCCTA     60
BnaC01g26360D      ATGGCTCTCGACACTCTCAATTCTCCGAACTCCACCACCACCGCTCCCTCTCCATTCCTA     60
BnaC01g26370D      ATGGCTCTCGACACTCTCAATTCTCCGAACTCCACGACCACCGCTCCCTCTCCATTCCTA     60
MyBnaC07g31600D    ATGGCACTCGACACTCTCAATTCTCCAACCTCCAC---CACCGCTCCTCCTCCTTTCCTA     57
MyBnaA03g40650D    ATGGCTCTCGACACTCTCAATTCTCCAACCTCCAC---CACCGCTCCTCCTCCTTTCCTA     57
                   ***** ******************** * ******   *********  **** ******

BnaA01g21030D      ACCGAGCCGGAGAATCTTGAGCCGTGGACCAAAAGAAAACGCACAAAACGACACCGTATA     120
BnaC01g26360D      ACCGAGCCGGAGAATCTTGAGCCGTGGACCAAAAGAAAACGCACAAAACGACACCGTATA     120
BnaC01g26370D      ACCGAGCCGGAGAATCTTGAGCCGTGGACCAAAAGAAAACGCACAAAACGACACCGTATA     120
MyBnaC07g31600D    ACCAAGCCGGAGAATCTCGAGTCGTGGACCAAAAGAAAGCGCACAAAACGACACCGAACT     117
MyBnaA03g40650D    ACCGAGCCGGAGAATCTTGAGTCATGGACCAAAAGAAAACGCACAAAACGACACCGAACT     117
                   *** ************* *** * ************** ***************** *

BnaA01g21030D      GATGATAAATCCAATCCTCCTTCCGAAGAAGAGTATCTTGCTCTCTGCCTCCTCATGCTT     180
BnaC01g26360D      GATGATCAATCCAATCCTCCCTCCGAAGAAGAGTATTTTGCTCTCTGCCTCCTCATGCTT     180
BnaC01g26370D      GATGATAAATCCAATCCTCCTTCCGAAGAAGAGTATCTTGCTCTCTGCCTCCTCATGCTT     180
MyBnaC07g31600D    GTAGATCAGTCACACCCTCCTTCCGAAGAAGAGTATCTTGCTATCTGCCTCCTCATGCTT     177
MyBnaA03g40650D    GTAGATCAGTCACACCCTCCTTCCGAAGAAGAGTATCTTGCTATCTGCCTCCTCATGCTT     177
                   *  *** * **  * ***** *************** ***** *****************

BnaA01g21030D      GCTCGTGGCTCCTCC---------------GATGATGATCATCACTCTTCTCCTCCTCCT     225
BnaC01g26360D      GCTCGTGGCTCCTCC---------------GATGATGATCAGCACTCTTCTCC---TCCT     222
BnaC01g26370D      GCTCGTGGCTCCTCC------TCCACCAATGATGGTAATGATCATCCCTCTCC---TCCT     231
MyBnaC07g31600D    GCTCGTGGCTCCTCC------TCCACCAATGATGGTAATGATCATCCCTCTCC---TCCT     228
MyBnaA03g40650D    GCTCGTGGCTCCTCCTCCTCCTCCTCCAATGATGGTAATGATCATCACTCTCC---TCCT     234
                   ***************               **** * ** * **    *****   ****

BnaA01g21030D      CCTCCACCGTCTGATCACCACCATCGAGACTATAAGTGCTCAGTCTGTGGCAAATCTTTC     285
BnaC01g26360D      CATCCACCGTCTGATCACCACCATCGAGACTACAAGTGCTCAGTCTGTGGCAAATCTTTC     282
BnaC01g26370D      GCTCCACCGTCCGATCATCACCACCGAGACTACAAGTGCTCCGTCTGTGGAAAATCTTTC     291
MyBnaC07g31600D    GCTCCACCGTCCGATCATCACCACCGAGACTACAAGTGCTCCGTCTGTGGAAAATCTTTC     288
MyBnaA03g40650D    GCTCCACCGTCCGATCATCACCACCGAGACTACAAGTGCTCCGTCTGTGGCAAATCTTTC     294
                     ********* ***** ***** ******** ******** ******** *********

BnaA01g21030D      CCGTCTTACCAAGCGTTAGGTGGACACAAAACCAGTCACCGGAAACCGGTTAGTAATAAT     345
BnaC01g26360D      CCGTCTTACCAAGCGTTAGGTGGACACAAAACCAGTCACCGGAAACCGGTTAGTAATAAT     342
BnaC01g26370D      CCGTCTTATCAAGCGTTAGGTGGACACAAAACCAGTCACCGGAAACCGGTTAGTAATAAT     351
MyBnaC07g31600D    CCGTCTTATCAAGCGTTAGGTGGACACAAAACCAGTCACCGGAAACCGGTTAGTAATACT     348
MyBnaA03g40650D    CCGTCTTATCAAGCGTTAGGTGGACACAAAACCAGTCACCGGAAACCGGTTAGTAATACT     354
                   ******** ************************************************* *

BnaA01g21030D      A---ATTATAACCACGACGATAATAATAACAGTGGTAACGGTTCCATTACTAATAACGGA     402
BnaC01g26360D      AATAATAATAACCACGACGGTCATAATAACAGTGGTAACGGTTCCATTACTAATAACGGA     402
BnaC01g26370D      AATAATAATAACCACGACGGTCATAATAACAGTGGTAACGGTTCCATTACTAATAACGGA     411
MyBnaC07g31600D    AATT---------GCCAAGATACTATTAACACTGGTAACGGTTCCGTTACTAATAACGGA     399
MyBnaA03g40650D    AATT---------GCCAAGATACTAATAACAGTGCTAACGGTTCCGTTACTAATAACGGA     405
                   *             * * * *  ** ***** ** ********** **************

BnaA01g21030D      AATATTAGTAACGGTTTGATTGGTCAAAGTGGAAAGACTCATAAGTGCTCCATCTGTTAT     462
BnaC01g26360D      AATATTAGTAACGGTTTGATTGGTCATAGTGGAAAGACTCATAAGTGCTCCATCTGTTTT     462
BnaC01g26370D      AATATTAGTAACGGTTTGATTGGTCATAGTGGAAAGACTCATAAGTGCTCCATCTGTTTT     471
MyBnaC07g31600D    AATATTA------------------------------CTCATAAGTGCTCTATCTGTTTT     429
MyBnaA03g40650D    AATATTAGTAACGGTTTAATTAGTCAAAGTGGGAAGACTCATAAGTGCTCTATCTGTTTT     465
                   *******                              ************* ******* *

BnaA01g21030D      AAGTCGTTTCCATCTGGTCAAGCGTTGGGCGGTCACAAACGGTGTCATTACGACGGTGGT     522
BnaC01g26360D      AAGTCGTTTCCATCTGGTCAAGCGTTGGGCGGTCACAAACGGTGTCATTACGACGGTGGT     522
BnaC01g26370D      AAGTCGTTTCCATCTGGTCAAGCGTTGGGCGGTCACAAACGGTGTCATTACGACGGTGGT     531
MyBnaC07g31600D    AAGTCGTTTCCCTCTGGTCAAGCACTGGGTGGTCACAAACGGTGTCATTACGACGGTGGT     489
MyBnaA03g40650D    AAGTCGTTTCCATCTGGTCAAGCATTGGGTGGTCACAAACGGTGTCATTACGACGGTGGT     525
                   *********** ***********  **** ******************************

BnaA01g21030D      AACAGTAATAGCAACATTAACGGGAACGGTAGTAACAGCCACGGGTTTGACCTGAACTTA     582
BnaC01g26360D      AACGGTAATAGCAACATTAACGGTAACGGTAGTAACAACCACGGGTTTGACCTGAACTTA     582
BnaC01g26370D      AACGGTAATAGCAACATTAACGGTAACGGTAGTAACAACCACGGGTTTGACCTGAACTTA     591
MyBnaC07g31600D    AACG------------------GTAACGGTAGTAATAATCACGGCTTTGACCTGAATTTA     531
MyBnaA03g40650D    AACG------------------GTAACGGTAGTAATAATCACGGCTTTGACCTGAATTTA     567
                   ***                   * *********** *  ***** *********** ***
```

```
BnaA01g21030D       CCGGCCGATCAAGTATTTGAAGTTTCTTGCGATGAGACACTGGGGAAAAGTCAACTCTCC     642
BnaC01g26360D       CCGGCCGATCAAGTATTTGAAGTTTCTTGCGATGATACACTTGGGAAAAGTCAGCTCTCC     642
BnaC01g26370D       CCGGCCGATCAAGTATTTGAAGTTTCTTGCGATGATACACTTGGGAAAAGTCAGCTCTCC     651
MyBnaC07g31600D     CCGGCTGATCA------------------CGATGAAACACTTGTAAGAG-TCAACTCTCC     572
MyBnaA03g40650D     CCGGCTGATCA------------------CGATGAAACACTTGTAAAATGTTAA------     603
                    ***** *****                  ****** ***** *   * *   * *

BnaA01g21030D       AGTGAAGAAACAAAGTCGCCGTTGTGA----------         669
BnaC01g26360D       GGCGAAGAAACAAAGTCGCCGTTGTGAT---------         670
BnaC01g26370D       GGCGAAGAAACAAAGTCGCCGTTGTGA----------         678
MyBnaC07g31600D     GGTGAAGAATCAAAGTCGGTGTCGTGATTATTATTAA         609
MyBnaA03g40650D     -------------------------------------         603
```

BnaA01g21030D, BnaC01g26360D and BnaC01g26370D have 13 nucleotides differences and two gaps.
Tandem duplications BnaC01g26360D and BnaC01g26370D have 19 nucleotides differences. BnaA01g21030D vs BnaC01g26360D contain 16 nucleotide differences and 1 gap.
BnaA01g21030D vs BnaC01g26370D have 23 nucleotides differences and one gap.
MyBnaC07g31600D and MyBnaA03g40650D have 12 nucleotide differences and 2 gaps.

Zat6-like

```
MyBnaCnng20570D      ATGGCACTTGAAGCTCTTAGTTCGCCAAGATTAGCTTCTCCAGTTCCAACTCTGTTTCAA     60
BnaA10g25850D        ATGGCACTTGAAGCTCTAAGTTCGCCAAGATTAGCTTCTCCGGTTCCAACTCTGTTTCAA     60
                     ***************** *********************** ******************

MyBnaCnng20570D      GATTATGCTGTTGGCTTCCATGGAAGCAAAGGCAAGCGATCTAAGAGGTCAAGATCCGAG     120
BnaA10g25850D        GATTCTGCTGTTGGCTTCCATGGTAGCAAAGGCAAACGATCTAAGCGGTCAAGATCCGAG     120
                     **** ****************** *********** ********* **************

MyBnaCnng20570D      TTCGACCGCAGTCTCACGGAGGATGAGTATATCGCTTTATGTCTCATGCTTCTTGCTCGT     180
BnaA10g25850D        TTCGACCGCAGTCTCACTGAGGATGAGTATATCGCTTTGTGTCTCATGCTTCTTGCTCGC     180
                     ***************** ******************** ******************** 

MyBnaCnng20570D      GACGGTAATCGAACCCGAGACCTGCCTTCTTGTTCTTCCTTGCCGCCTCTGCTTCCTACT     240
BnaA10g25850D        GACGGGAATCGAACCCGCCACCTGCCTTCTTCTTCTTCCTCGCCGCCTCTGCTTCCTACT     240
                     ***** ***********  ************ ******** *******************

MyBnaCnng20570D      CCTACTTCTACACATACCCACAAGTGCAGCGTCTGCGACAAGGCGTTTTCTTCTTACCAG     300
BnaA10g25850D        CTTACTTCTACTCATCTCCACAAGTGCAGCGTCTGCGACAAGGCGTTTTCTTCTTACCAG     300
                     * ********* ***  *******************************************

MyBnaCnng20570D      GCTCTCGGCGGGCACAAGGCGAGTCACCGTAAAAAGTCATCGCAGACTCAGTCTAGCGGA     360
BnaA10g25850D        GCTCTCGGTGGGCACAAGGCGAGTCACCGGAAAAACTCATCGCAGACTCAGTCTAGCGGA     360
                     ******** ******************** ***** ************************

MyBnaCnng20570D      GGAGATGAGAAATCCACCTCGTCGGCTATAACTATCGTGAGACACGGCG---------GA     411
BnaA10g25850D        GGAGATGAGAAATCCACGTCGTCGGCGATAACCATCGCGAGCCACGGCGGCGGCGGAGGA     420
                     ***************** ******** ***** **** *** *******         **

MyBnaCnng20570D      GGAAGTGTGAAACCGCACGTTTGCTCGATCTGCAACAAGTCGTTCGCGACAGGCCAAGCT     471
BnaA10g25850D        GGAAGTGTGAAATCTCACGTTTGCTCGATCTGCAACAAGTCTTTCGCGACAGGTCAAGCG     480
                     ************ * ************************** *********** ***** 

MyBnaCnng20570D      CTCGGTGGCCACAAACGGTGCCACTACGAAGGCAAGAACGGCGGCGGTGGGAGCAGCAGC     531
BnaA10g25850D        CTCGGGGGCCACAAACGGTGCCACTACGAAGGCAAGAACGG---------GAGCAGCAG-     530
                     ***** ***********************************         ********* 

MyBnaCnng20570D      GTGTCTATCTCCGAAGGCGTGGGGTCCACAAGCCACGTCAGCAGCGGCAGCCATCATCAC     591
BnaA10g25850D        -----------CGAAGGTGTGGGGTCCACAAGCCACGTCAGCAGCGGCAGCCATCA----     575
                                ****** **************************************    

MyBnaCnng20570D      CACCACCACCACCACCGTGGGTTTGACCTCAACATCCCGCCGATACCGGCGTTCTGGACG     651
BnaA10g25850D        --------CCACCACCGTGGGTTTGACCTCAACATCCCGCCGATACCGGAATTCTCGACG     627
                             *****************************************  **** ****

MyBnaCnng20570D      GTCAACGGAGAAGAAGAGGTGATGAGCCCCATGCCGACCAAGAAACTGAGGCTCGAGTAG     711
BnaA10g25850D        GTCAACGGAGAAGAAGAGGTGATGAGCCCCATGCCGACCAAGAAAATGAGGCTCGAGTAG     687
                     ********************************************* **************
```

BnaA10g25850D and MyBnaCnng20570D have 33 nucleotide differences and four gaps.

AZF3-like

```
BnaC07g16940D       ATGGCGCTTGAAGCTCTAAATTCACCGAGGATAGCTTCTCCAGTTCCGACTCTGTTCGAG      60
BnaA06g36830D       ATGGCGCTTGAAGCTCTAAATTCACCGAGGATAGCTTCTCCAGTCCCGACTCTGTTCGAG      60
                    ******************************************** ***************

BnaC07g16940D       GAGCACTGGACCAAAGGTAAGCGGTCCAAAAGATCCAGATCTGATCATCCTCACCGTCTC      120
BnaA06g36830D       GAGCACTGGACCAAACGTAAGCGGTCCAAAAGATCCAGATCTGATCATCTCCACCGTCTC      120
                    *************** *********************************  *********

BnaC07g16940D       ACTGAGGAAGAGTACCTCGCTTTCTGCCTCATGCTTCTTGCTCGCGACGGCGATCGCCAT      180
BnaA06g36830D       ACCGAGGAAGAATACCTCGCTTTCTGCCTCATGCTTCTTGCTCGCGACGGCGACCGCCAT      180
                    ** ******** ***************************************** ******

BnaC07g16940D       CACGTGGAGGAGGAAAAGACTGTTTACAAGTGCGGCGTTTGTGACAAGGTGTTTCTTTCT      240
BnaA06g36830D       CACGTGGAGGAGGAAAAGACCGTTTACAAGTGCGGCGTTTGTGACAAGGTGTTTCTCTCT      240
                    ******************** *********************************** ***

BnaC07g16940D       TACCAAGCTCTCGGTGGTCACAAGGCGAGTCACCGGAATTTTTCCAGCGGTGGAGATGTT      300
BnaA06g36830D       TACCAAGCTCTCGGCGGTCACAAGGCGAGTCACCGGAATCTGTCCAGCGGTGGAGATGTT      300
                    ************** ************************ * ******************

BnaC07g16940D       AAGCCGACGACACCGGCCGCCGTGAAGTCACACGTTTGCTCGATATGTCATAAATCATTC      360
BnaA06g36830D       AAGCCGACGACGCCGTCCGCCGTGAAGTCTCACGTTTGCTCGATATGTCATAAATCATTC      360
                    *********** *** ************* ******************************

BnaC07g16940D       ACCACCGGTCAAGCTCTTGGCGGCCACAAGCGGTGCCACTATGATGGAAGTAGTAACGTT      420
BnaA06g36830D       GCCACCGGTCAAGCTCTTGGCGGCCACAAGCGGTGCCACTATGATGGAAGTAGTAGCGTT      420
                     ****************************************************** ****

BnaC07g16940D       GTTTCTAATTCTGAAGGTGTGGGGTCTACTAGCCACGTCAGCGGAAATAGCCGCCGTGGA      480
BnaA06g36830D       GTTTCGAATTCTGAAGGTGTGGGGTCTACTAGCCACGTCAG------------CCGTGGA      468
                    ***** ***********************************            *******

BnaC07g16940D       TTCGACCTTAACATCACGCCCATAAATGAATTATCGCCGGACGATGAAGTGAAGAGCCCG      540
BnaA06g36830D       TTCGACCTTAACATCACCCCCGTAAATGAATTCTCGCCGGACGATGAAGTGTTGAGCCCC      528
                    ***************** *** ********** ******************  ****** 

BnaC07g16940D       TTGCCGTCGAAGAAGCTCCGCCTCACGTAA         570
BnaA06g36830D       TTGCCGTCGAAGAAGCTCCGCCTCAAGTAA         558
                    ************************* ****
```

BnaC07g16940D and BnaA06g36830D have 23 nucleotide differences and one gap.

AZF1-like

```
BnaA06g25060D         ATGGCTCTTGAGACTCTCAATTCCCCGACTTCAGCCACCGCCACCGCTCGGCCTCTTCTC     60
BnaC03g48520D         ATGGCTCTTGAGACCCTCAATTCCCCGACTTCAGCCACCGCCACGGCTCTGCCTCTTCTC     60
MyBnaC02g16730D       ATGGCTCTTGAGACTCTCAATTCTCCGAGTTCAGCCACCGCCTCCGCTCGGCCTCTTCTC     60
MyBnaAnng05120D       ATGGCTCTTGAGACTCTCAATTCTCCGACTTCAGCCACCGCCTCCGCTCGGCCTCTTCTC     60
MyBnaA09g07340D       ATGGCTCTCGAGACTCTCAACTCTCCTACTTCCGCCACCGCCACCGCTCGGCCTTTTCTC     60
MyBnaC09g07240D       ATGGCTCTTGAGACTCTCAACTCTCCTACTTCCGCCACCGCCGCCGCTCGGCCTTTTCTC     60
                      ******** ***** ***** ** ** * *** ********* * **** **** *****

BnaA06g25060D         CGGTATCGTGAAGAAATGGAGCCGGATAATCTCGAGCAATGGGCTAAAAGAAAACGCACC     120
BnaC03g48520D         CGGTATCGTGAAGAAATGGAGCCGGATAATCTCGAGCAGTGGGCTAAAAGAAAACGCACC     120
MyBnaC02g16730D       CGGTATCGCGAAGAAATGGAGCCGGAGAATCTCGAGCAATGGGCTAAAAGAAAACGCACC     120
MyBnaAnng05120D       CGGTATCGCGAAGAAATGGAGCCGGAGAATCTCGAGCAATGGGCTAAAAGAAAACGCACC     120
MyBnaA09g07340D       CGGTATCGCAAAGAAATGGAGCCGGAGAATCTCGAGCAGTGGGCTAAAAGAAAACGTACC     120
MyBnaC09g07240D       CGGTATCGCGAAGAAATGGAGCCGGAGAATCTGGAGCAGTGGGCTAAAAGAAAACGTACC     120
                      ********  **************** ***** ***** ***************** ***

BnaA06g25060D         AAGCGACAACGTCTTGATCAGAACCATCACAATCAAGAAACCACTCCTTCCGAAGAAGAG     180
BnaC03g48520D         AAGCGACAACGTCTTGATCAGAATCATCACAATCAAGAAACCACTCCTTCCGAAGAAGAG     180
MyBnaC02g16730D       AAACGACAACGTTTTGATCAGAGTCGTCTGGATCAAGAAACGGCTCCTTCAGAAGAAGAG     180
MyBnaAnng05120D       AAACGACAACGTTTTGATCAGAGTCGTCTGAATCAAGAAACGGCTCCTTCAGAAGAAGAG     180
MyBnaA09g07340D       AAACGTCAACGTTTTGATCACA---------ACCAAGAAAAGACTCCTTCCGAAGAAGAG     171
MyBnaC09g07240D       AAACGTCAACGTTTTGATCACA---------ATCAAGAAAAGACTCCTTCCGAAGAAGAG     171
                      ** ** ****** ******* *         * *******   ******* *********

BnaA06g25060D         TATCTCGCTCTGTGTCTCCTCATGCTCGCTCGTGGAACCGCCGTACAACCTCCTCCTACT     240
BnaC03g48520D         TATCTCGCTCTTTGCCTCCTCATGCTCGCTCGTGGAACCGCCGTGCAACCGCCTCTTACT     240
MyBnaC02g16730D       TATCTCGCTCTTTGTCTCCTCATGCTCGCTCGTGGCTCCGCCGTGAAATCTCCTCTCCGT     240
MyBnaAnng05120D       TATCTCGCTCTTTGTCTCCTCATGCTCGCTCGTGGCTCCGCCGTGCAATCTCCTCTCCCT     240
MyBnaA09g07340D       TATCTAGCTCTTTGTCTCCTCATGCTCGCTCGTGGCTCCACTGTACAATCTCTTCCTCCG     231
MyBnaC09g07240D       TATCTAGCTCTTTGTCTCCTCATGCTTGCTCGTGGCTCCACCGTAAAATCTCCTCCTCCG     231
                      ***** ***** ** *********** ********    ** * **   ** * * **

BnaA06g25060D         CCGCCTCCGCCGTCACGTCGGTTGTCTTCCGATCACCGTGATTTCAAGTGTACGGTCTGT     300
BnaC03g48520D         CCGCCTCCGCCGTCACATCGGTCTCGTTCCGATCACCGTGATTTCAAGTGCACGGTCTGT     300
MyBnaC02g16730D       CCGTC---ACCG------------TCCTCCGACCACCGTGGTTACAAGTGTACGGTCTGC     285
MyBnaAnng05120D       CCGTC---TTCG------------TCCTCCGACCACCGTGGTTACAAGTGTACGGTCTGC     285
MyBnaA09g07340D       CCGTCTCTCCCG------------TCATCGGATCACCGTGGCTACAAGTGTACGGTCTGT     279
MyBnaC09g07240D       CCGTCTCTCCCG------------TCGTCGGATCACCGTGGTTACAAGTGCACGGTCTGT     279
                      *** *     **               ** ** *******  * ****** ********

BnaA06g25060D         GGAAAATCGTTTAACTCTTACCAAGCCTTGGGTGGACACAAGACGAGTCACCGAAAACCG     360
BnaC03g48520D         GGAAAGTCGTTTAACTCTTACCAAGCCTTGGGTGGTCACAAGACGAGTCACCGGAAACCG     360
MyBnaC02g16730D       GGAAAGTCGTTTTCCTCTTACCAAGCCTTAGGTGGACACAAGACCAGTCACCGGAAACCG     345
MyBnaAnng05120D       GGAAAGTCGTTTTCCTCTTACCAAGCCTTAGGTGGACACAAGACGAGTCACCGGAAACCG     345
MyBnaA09g07340D       GGAAAGTCCTTTTCCTCTTACCAAGCCTTAGGTGGACACAAGACCAGCCACCGGAAACCG     339
MyBnaC09g07240D       GGAAAGTCTTTTTCCTCTTACCAAGCCTTAGGTGGACACAAGACCAGTCACCGGAAACCG     339
                      ***** ** ***  *************** ***** ******** ** ***** ******

BnaA06g25060D         CCAGCGAATAACGCTAACGTTCCAAGCGGCCAAGAGCCATCTAATAACA---AGAGTCAC     417
BnaC03g48520D         CCGGCGAATAACGTTAACGTTCCGAGCAGCCAAGAGCCATCTAATAACA---ACAGTCAC     417
MyBnaC02g16730D       ---GCGAGCAACGTTAACGTTCCCATCAACCAAGAGCCGTCTAATAACAGTCATAGTAAC     402
MyBnaAnng05120D       ---GCGAGCAACGTTAACGTTCCCATCAACCAAGAGCAGTCTAATAACAGTCATAGTAAC     402
MyBnaA09g07340D       ---GTGAATAACACTGACGTTCCTAGTAACCAAGAGCCGTTTAATAATACTCACAGAAAC     396
MyBnaC09g07240D       ---GTAAACACTAATAACGGTCCCAGTGCCCAAGAGCCGTCTAATAAGACTCACGGTAAC     396
                         *  *  *    * *** *** *    ********  * ****** *    *  *  **

BnaA06g25060D         AGTAACGGTGGTTCCGTTATTTTTAACGGTAACGGTACTGTTAGTAACGGTGTTAATCAG     477
BnaC03g48520D         GGTAACGGTGGTTCCGTTGTTTTTAGCGGTAACGGTACTGCCAGTAACGGTGTTAATCTA     477
MyBnaC02g16730D       AGCAACGGTGGTTCCGTCGTTATCAACGGTAA---------------CGGCTTAGTCAA     447
MyBnaAnng05120D       AGCAACGGTGGTTCCGTCGCTATCAACGGTAA---------------CGGCGTTAGTCAA     447
MyBnaA09g07340D       AGTAACGGCGGTTCCGTTGTTATTAATGGTAA---------------CGGTGTTAGTCAG     441
MyBnaC09g07240D       AGTAACGGCGGTTCCATTGTTATTAACGGTAA---------------CGGTGTAAGTCAG     441
                       * ***** ****** *    * * *  *****               ***  * * **
```

```
BnaA06g25060D        AGCGGAAAGATTCACACTTGTTCAATCTGTTTCAAGTCGTTTTCGTCTGGTCAAGCATTA     537
BnaC03g48520D        AGCGGAAAGATTCACACTTGTTCAATCTGTTTCAAGTCGTTTTCGTCTGGTCAAGCATTA     537
MyBnaC02g16730D      AGCGGGAAGATTCATACTTGCTCGATATGTTTCAAGTCGTTTTCGTCAGGTCAAGCGTTG     507
MyBnaAnng05120D      AGCGGGAAGATTCATACTTGCTCGATATGTTTCAAGTCGTTTTCGTCAGGTCAGGCTTTG     507
MyBnaA09g07340D      AGCGGAAAGACTCATACATGTTCTATATGTTTCAAGTCGTTTTCGTCTGGTCAAGCCTTA     501
MyBnaC09g07240D      AGCGGAAAGACTCATACATGTTCTATATGTTTCAAGTCGTTTTCGTCTGGTCAAGCCTTA     501
                     ***** **** *** ** ** ** ** ******************** ***** ** **

BnaA06g25060D        GGTGGACACAAACGGTGTCACTATGACGGTGGTAATAACGGAAACGGTAACGGC------     591
BnaC03g48520D        GGTGGACACAAACGGTCTCACTATGACGGTGGTAATAACGGAAACGGTAACGGC------     591
MyBnaC02g16730D      GGTGGACACAAACGGTGTCACTATGACGGTGGTAATAACGGAAACGGTAACGGCAGTAGC     567
MyBnaAnng05120       GGTGGACACAAACGGTGTCACTATGACGCTGGTAATAACGGAAACGGTAACGGCAGTAGC     567
MyBnaA09g07340D      GGTGGACACAAACGATGTCACTATGACGGTGGTAATAAC------GGTAACGGCAGTAGC     555
MyBnaC09g07240D      GGTGGACACAAACGATGTCACTATGACGGTGGCAATAAC------GGTAACGGCAGTAGC     555
                     ************** * *********** *** ******      *********

BnaA06g25060D        ------AGCGTGGAAGTCATGGGTGGCAGTGACGTCAGCGACGTGGATGACGAAAGATCG     645
BnaC03g48520D        ------AGCGTGGAAGTCATGGGTGGCAGTGACGTCAGCGACGTGGATGACGAAAGATCG     645
MyBnaC02g16730D      AGCAACAGCGTGGAGGTCGTCGGTGGCAGTGACGGCAGCTATGTGGATGATGAAAGATCG     627
MyBnaAnng05120D      AGCAACAGCGTGGAGGTCGTCGGTGGCAGTGACGGCAGCTATGTGGATGATGAAAGATCG     627
MyBnaA09g07340D      AGCAACAGCGTTGAAGTCATCGGGGGTAGTGACGTCAGCGATGTAGACGATGAAAGATCA     615
MyBnaC09g07240D      AGCAACAGCGTTGAAGTCATCGGTGGTAGTGACGTCAGCGATGTAGACGATGAAAGATCT     615
                           ***** ** *** * ** ** ******* **** * ** ** ** ********

BnaA06g25060D        TCGGAACAAAGCGCGATCGGCGGCCACCGTGGATTTGACTTAAACTTACCGGCTGATCAA     705
BnaC03g48520D        TCGGAACAAACCGCGTTCGGGGGCCACCGTGGATTTGACTTAAACTTACCGGCTGATCAA     705
MyBnaC02g16730D      TCAGAACAGAGCGCGACCGGCGACAACCGGGGGTTTGACTTGAATTTACCGGCTGATCAA     687
MyBnaAnng05120D      TCAGAACAGAGCGCGACCGGCGACAACCGGGGGTTTGACTTGAATTTACCGGCTGATCAA     687
MyBnaA09g07340D      TCGGAACAGAGCGGTATAGGCGGCCACCGTGGATTTGACTTAAATTTACCGGCTGATCAA     675
MyBnaC09g07240D      TCGGAACAAAGCGCTATAGGCGGCCACCGTGGATTTGACTTAAATTTACCGGCTGATCAA     675
                     ** ***** * **      ** * * **** ** ******** ** ***************

BnaA06g25060D        GTCTCGGTGGTTATTTCTTAA      726
BnaC03g48520D        GTCTCGGTGGTTATTGCTTAA      726
MyBnaC02g16730D      GTCGCAGTTGTGATATCTTAA      708
MyBnaAnng05120D      GTCGCAGTTGTGATATCTTAA      708
MyBnaA09g07340D      GTCACGGTGGTGATTTCTTAA      696
MyBnaC09g07240D      GTCACGGTGGTGATTTCTTAA      696
                     *** * ** ** **  *****
```

BnaA06g25060D and BnaC03g48520D have 31 nucleotide differences.
MyBnaA09g07340D and MyBnaC09g07240D have 29 nucleotide differences.
MyBnaC02g16730D and BnaAnng05120D have 10 nucleotide differences.

Table 1 SRA data used to verify transcription of gene and retention of intron sequence

Group	Arabidopsis Gene name	Gene ID	B napus ortholog	SRA* tissue	transcript (bp)**	100% ID (bp)**
**2i-A**	Zat11	BnaAZFP9	BnaA03g17210D	root rep1	546	8-524
		BnaCZFP19	BnaC04g45160D	root rep 1	543	9-534
		BnaAZFP16	BnaA04g21410D	root rep 1	543	9-534
		BnaCZFP36	BnaCnng48130D	root rep 1	540	1-532
		BnaAZFP17	BnaA05g07070D	root rep 1	543	6-535
		BnaCZFP14	BnaC04g07880D	root rep 1	543	5-536
	Zat18	BnaAZFP13	BnaA04g04630D	root rep 1	525	1-483
		BnaCZFP17	BnaC04g27120D	none	504	*
		BnaAZFP25	BnaA07g37620D	root rep 1	507	2-507
		BnaCZFP25	BnaC06g14860D	root rep 1	507	12-483
**2i-B**	Zat17	BnaAZFP15	BnaA04g16610D	root rep 1	447	2-446
		BnaAZFP27	BnaA07g14080D	root rep 1	453	7-447
		BnaCZFP18	BnaC04g39930D	root rep 1	447	2-446
		BnaCZFP15	BnaC04g15490D	root rep 1	453	1-452
	Zat12	BnaAZFP32	BnaA10g12780D	root rep 1	483	1-478
		BnaCZFP30	BnaC09g35160D	root rep 1	486	1-481
		BnaCZFP10	BnaC03g11570D	root rep 1	477	3-473
		BnaAZFP8	BnaA03g09250D	root rep 1	480	16-468
		BnaAZFP5	BnaA02g06790D	root rep 1	483	3-481
		BnaAZFP4	BnaA02g06780D	root rep 1	474	7-472
	Zat16	BnaAZFP21	BnaA06g19880D	flower bud	510	1-499
		BnaAZFP20	BnaA06g18210D	root rep 1	579	1-579
		BnaCZFP12	BnaC03g55590D	none	615	1-615
		BnaAZFP21	BnaA06g19890D	root rep 3	513	5-497
		BnaCZFP1	BnaC01g24440D	root rep 1	534	1-534
**2i-C**	Zat5	BnaCZFP16	MyBnaC04g16100D	flower bud	882	5-882
		BnaCZFP32	MyBnaCnng66450D	root rep 3	864	7-850
		BnaAZFP14	MyBnaA04g16320D	leaf rep	864	29-862

				1		
		BnaAZFP26	MyBnaA07g13700D	root rep 1	867	3-860
	Zat15	BnaCZFP20	MyBnaC05g42550D	root rep 3	1188	2-1181
		BnaAZFP19	MyBnaA05g28380D	root rep 1	1155	4-1125
		BnaAZFP3	MyBnaA01g31690D	root rep 3	1155	45-1128
	Zat14	BnaAZFP6	MyBnaA03g00910D	root rep 1	831	18-824
		BnaAZFP12	MyBnaA03g46860D	leaf rep 1	798	1-791
		BnaCZFP8	MyBnaC03g01240D	root rep 1	831	2-825
		BnaCZFP5	BnaC02g03060D	root rep 3	807	5-800
		BnaCZFP35	BnaCnng02770D	root rep 3	798	5-779
		BnaAZFP36	BnaAnng00680D	root rep 1	807	7-801
		BnaAZFP35	BnaA10g26510D	root rep 1	798	5-791
	At5g04390	BnaAZFP7	MyBnaA03g01240D	none		*
		BnaAZFP37	MyBnaAnng17660D	flower bud	1053	1-1053
		BnaCZFP7	MyBnaC03g71210D	flower bud	1089	3-1089
		BnaAZFP33	MyBnaA10g25770D	root rep 1	1038	5-1031
		BnaCZFP31	MyBnaC09g50520D	root rep 3	1040	12-1034
**2i-D**	Zat10	BnaCZFP26	BnaC07g11700D	root rep 1	696	6-687
		BnaAZFP30	BnaA09g27780D	root rep 1	690	25-680
		BnaCZFP22	BnaC05g21480D	root rep 1	690	25-686
		BnaAZFP28	BnaA08g18790D	root rep 1	702	2-684
		BnaCZFP13	BnaC03g58080D	root rep 1	701	10-694
	AZF2	BnaAZFP2	BnaA01g26030D	root rep 1	795	15-768
		BnaCZFP4	BnaC01g33270D	root rep 1	786	7-778
		BnaAZFP18	BnaA05g20490D	root rep 1	804	2-789
		BnaCZFP23	BnaC05g33170D	root rep 1	801	26-800
		BnaAZFP10	BnaA03g35310D	root rep 1	810	18-784
		BnaCZFP9	BnaC03g73930D	root rep 1	807	2-800
	Zat13	BnaAZFP1	BnaA01g21030D	root rep	669	9-669

				3		
		BnaCZFP2	BnaC01g26360D	root rep 3	670	1-664
		BnaCZFP3	BnaC01g26370D	root rep 3	678	9-673
		BnaCZFP28	MyBnaC07g31600D	root rep 3	609	2-609
		BnaAZFP11	MyBnaA03g40650D	root rep 3	603	26-599
	Zat6	BnaCZFP34	MyBnaCnng20570D	root rep 3	711	14-693
		BnaAZFP34	BnaA10g25850D	root rep 3	687	3-646
	AZF3	BnaCZFP27	BnaC07g16940D	root rep 3	570	3-566
		BnaAZFP24	BnaA06g36830D	root rep 3	558	7-552
	AZF1	BnaAZFP23	BnaA06g25060D	root rep 3	726	7-107
		BnaCZFP11	BnaC03g48520D	root rep 3	726	9-712
		BnaAZFP29	MyBnaA09g07340D	root rep 3	696	10-691
		BnaCZFP29	MyBnaC09g07240D	root rep 3	696	3-691
		BnaCZFP6	MyBnaC02g16730D	root rep 3	708	7-705
		BnaAZFP38	MyBnaAnng05120D	root rep 3	708	9-705
**2i-X**	At1g02040	BnaCZFP7	BnaCnng09030D	flower bud	777	36-772

*SRA (sequence read archive) root rep 1 ERX397800, flower bud ERX397796, Root rep 3 ERX397799 leaf rep 1 ERX397788
**(bp) base pair
